# Circulating free testosterone and risk of aggressive prostate cancer: Prospective and Mendelian randomisation analyses in international consortia

**DOI:** 10.1002/ijc.34116

**Published:** 2022-06-07

**Authors:** Eleanor L. Watts, Aurora Perez-Cornago, Georgina K. Fensom, Karl Smith-Byrne, Urwah Noor, Colm D. Andrews, Marc J. Gunter, Michael V. Holmes, Richard M. Martin, Konstantinos K. Tsilidis, Demetrius Albanes, Aurelio Barricarte, Bas Bueno-de-Mesquita, Chu Chen, Barbara A. Cohn, Niki L. Dimou, Luigi Ferrucci, Leon Flicker, Neal D. Freedman, Graham G. Giles, Edward L. Giovannucci, Gary E. Goodman, Christopher A. Haiman, Graeme J. Hankey, Jiaqi Huang, Wen-Yi Huang, Lauren M. Hurwitz, Rudolf Kaaks, Paul Knekt, Tatsuhiko Kubo, Hilde Langseth, Gail Laughlin, Loic Le Marchand, Tapio Luostarinen, Robert J. MacInnis, Hanna O. Mäenpää, Satu Männistö, Jeffrey E. Metter, Kazuya Mikami, Lorelei A. Mucci, Anja W. Olsen, Kotaro Ozasa, Domenico Palli, Kathryn L. Penney, Elizabeth A. Platz, Harri Rissanen, Norie Sawada, Jeannette M. Schenk, Pär Stattin, Akiko Tamakoshi, Elin Thysell, Chiaojung Jillian Tsai, Shoichiro Tsugane, Lars Vatten, Elisabete Weiderpass, Stephanie J. Weinstein, Lynne R. Wilkens, Bu B. Yeap, The PRACTICAL Consortium, CRUK, BPC3, CAPS, PEGASUS, Rosalind A. Eeles, Christopher A. Haiman, Zsofia Kote-Jarai, Fredrick R. Schumacher, Sara Benlloch, Ali Amin Al Olama, Kenneth R. Muir, Sonja I. Berndt, David V. Conti, Fredrik Wiklund, Stephen Chanock, Ying Wang, Catherine M. Tangen, Jyotsna Batra, Judith A. Clements, APCB BioResource (Australian Prostate Cancer BioResource), Henrik Grönberg, Nora Pashayan, Johanna Schleutker, Demetrius Albanes, Stephanie Weinstein, Alicja Wolk, Catharine M. L. West, Lorelei A. Mucci, Géraldine Cancel-Tassin, Stella Koutros, Karina Dalsgaard Sørensen, Eli Marie Grindedal, David E. Neal, Freddie C. Hamdy, Jenny L. Donovan, Ruth C. Travis, Robert J. Hamilton, Sue Ann Ingles, Barry S. Rosenstein, Yong-Jie Lu, Graham G. Giles, Robert J. MacInnis, Adam S. Kibel, Ana Vega, Manolis Kogevinas, Kathryn L. Penney, Jong Y. Park, Janet L. Stanford, Cezary Cybulski, Børge G. Nordestgaard, Sune F. Nielsen, Hermann Brenner, Christiane Maier, Jeri Kim, Esther M. John, Manuel R. Teixeira, Susan L. Neuhausen, Kim De Ruyck, Azad Razack, Lisa F. Newcomb, Davor Lessel, Radka Kaneva, Nawaid Usmani, Frank Claessens, Paul A. Townsend, Jose Esteban Castelao, Monique J. Roobol, Florence Menegaux, Kay-Tee Khaw, Lisa Cannon-Albright, Hardev Pandha, Stephen N. Thibodeau, David J. Hunter, Peter Kraft, William J. Blot, Elio Riboli, Naomi E. Allen, Timothy J. Key, Ruth C. Travis

**Affiliations:** 1 The Institute of Cancer Research, London, SM2 5NG, UK; 2 Royal Marsden NHS Foundation Trust, London, SW3 6JJ, UK; 3 Center for Genetic Epidemiology, Department of Preventive Medicine, Keck School of Medicine, University of Southern California/Norris Comprehensive Cancer Center, Los Angeles, CA 90015, USA; 4 Department of Population and Quantitative Health Sciences, Case Western Reserve University, Cleveland, OH 44106-7219, USA; 5 Seidman Cancer Center, University Hospitals, Cleveland, OH 44106, USA; 6 Centre for Cancer Genetic Epidemiology, Department of Public Health and Primary Care, University of Cambridge, Strangeways Research Laboratory, Cambridge CB1 8RN, UK; 7 University of Cambridge, Department of Clinical Neurosciences, Stroke Research Group, R3, Box 83, Cambridge Biomedical Campus, Cambridge CB2 0QQ, UK; 8 Division of Population Health, Health Services Research and Primary Care, University of Manchester, Oxford Road, Manchester, M13 9PL, UK; 9 Division of Cancer Epidemiology and Genetics, National Cancer Institute, NIH, Bethesda, Maryland, 20892, USA; 10 Department of Medical Epidemiology and Biostatistics, Karolinska Institute, SE-171 77 Stockholm, Sweden; 11 ‘Department of Population Science, American Cancer Society, 250 Williams Street, Atlanta, GA 30303, USA; 12 SWOG Statistical Center, Fred Hutchinson Cancer Research Center, Seattle, WA 98109, USA; 13 Australian Prostate Cancer Research Centre-Qld, Institute of Health and Biomedical Innovation and School of Biomedical Sciences, Queensland University of Technology, Brisbane QLD 4059, Australia; 14 Translational Research Institute, Brisbane, Queensland 4102, Australia; 15 Australian Prostate Cancer Research Centre-Qld, Queensland University of Technology, Brisbane; Prostate Cancer Research Program, Monash University, Melbourne; Dame Roma Mitchell Cancer Centre, University of Adelaide, Adelaide; Chris O’Brien Lifehouse and; 16 Department of Applied Health Research, University College London, London, WC1E 7HB, UK; 17 Centre for Cancer Genetic Epidemiology, Department of Oncology, University of Cambridge, Strangeways Laboratory, Worts Causeway, Cambridge, CB1 8RN, UK; 18 Institute of Biomedicine, University of Turku, Finland; 19 Department of Medical Genetics, Genomics, Laboratory Division, Turku University Hospital, PO Box 52, 20521 Turku, Finland; 20 Department of Surgical Sciences, Uppsala University, 75185 Uppsala, Sweden; 21 Division of Cancer Sciences, University of Manchester, Manchester Academic Health Science Centre, Radiotherapy Related Research, The Christie Hospital NHS Foundation Trust, Manchester, M13 9PL UK; 22 Department of Epidemiology, Harvard T. H. Chan School of Public Health, Boston, MA 02115, USA; 23 CeRePP, Tenon Hospital, F-75020 Paris, France; 24 Sorbonne Universite, GRC n°5, AP-HP, Tenon Hospital, 4 rue de la Chine, F-75020 Paris, France; 25 Department of Molecular Medicine, Aarhus University Hospital, Palle Juul-Jensen Boulevard 99, 8200 Aarhus N, Denmark; 26 Department of Clinical Medicine, Aarhus University, DK-8200 Aarhus N; 27 Department of Medical Genetics, Oslo University Hospital, 0424 Oslo, Norway; 28 Nuffield Department of Surgical Sciences, University of Oxford, Room 6603, Level 6, John Radcliffe Hospital, Headley Way, Headington, Oxford, OX3 9DU, UK; 29 University of Cambridge, Department of Oncology, Box 279, Addenbrooke’s Hospital, Hills Road, Cambridge CB2 0QQ, UK; 30 Cancer Research UK, Cambridge Research Institute, Li Ka Shing Centre, Cambridge, CB2 0RE, UK; 31 Nuffield Department of Surgical Sciences, University of Oxford, Oxford, OX1 2JD, UK; 32 Faculty of Medical Science, University of Oxford, John Radcliffe Hospital, Oxford, UK; 33 Population Health Sciences, Bristol Medical School, University of Bristol, BS8 2PS, UK; 34 Cancer Epidemiology Unit, Nuffield Department of Population Health, University of Oxford, Oxford, OX3 7LF, UK; 35 Dept. of Surgical Oncology, Princess Margaret Cancer Centre, Toronto ON M5G 2M9, Canada; 36 Dept. of Surgery (Urology), University of Toronto, Canada; 37 Department of Preventive Medicine, Keck School of Medicine, University of Southern California/Norris Comprehensive Cancer Center, Los Angeles, CA 90015, USA; 38 Department of Radiation Oncology and Department of Genetics and Genomic Sciences, Box 1236, Icahn School of Medicine at Mount Sinai, One Gustave L. Levy Place, New York, NY 10029, USA; 39 Centre for Cancer Biomarker and Biotherapeutics, Barts Cancer Institute, Queen Mary University of London, John Vane Science Centre, Charterhouse Square, London, EC1M 6BQ, UK; 40 Cancer Epidemiology Division, Cancer Council Victoria, 615 St Kilda Road, Melbourne, VIC 3004, Australia; 41 Centre for Epidemiology and Biostatistics, Melbourne School of Population and Global Health, The University of Melbourne, Grattan Street, Parkville, VIC 3010, Australia; 42 Precision Medicine, School of Clinical Sciences at Monash Health, Monash University, Clayton, Victoria 3168, Australia; 43 Division of Urologic Surgery, Brigham and Womens Hospital, 75 Francis Street, Boston, MA 02115, USA; 44 Fundación Pública Galega Medicina Xenómica, Santiago de Compostela, 15706, Spain; 45 Instituto de Investigación Sanitaria de Santiago de Compostela, Santiago De Compostela, 15706, Spain; 46 Centro de Investigación en Red de Enfermedades Raras (CIBERER), Spain; 47 ISGlobal, Barcelona, Spain; 48 IMIM (Hospital del Mar Medical Research Institute), Barcelona, Spain; 49 Universitat Pompeu Fabra (UPF), Barcelona, Spain; 50 CIBER Epidemiología y Salud Pública (CIBERESP), 28029 Madrid, Spain; 51 Channing Division of Network Medicine, Department of Medicine, Brigham and Women’s Hospital/Harvard Medical School, Boston, MA 02115, USA; 52 Department of Cancer Epidemiology, Moffitt Cancer Center, 12902 Magnolia Drive, Tampa, FL 33612, USA; 53 Division of Public Health Sciences, Fred Hutchinson Cancer Research Center, Seattle, Washington, 98109-1024, USA; 54 Department of Epidemiology, School of Public Health, University of Washington, Seattle, Washington 98195, USA; 55 International Hereditary Cancer Center, Department of Genetics and Pathology, Pomeranian Medical University, 70-115 Szczecin, Poland; 56 Faculty of Health and Medical Sciences, University of Copenhagen, 2200 Copenhagen, Denmark; 57 Department of Clinical Biochemistry, Herlev and Gentofte Hospital, Copenhagen University Hospital, Herlev, 2200 Copenhagen, Denmark; 58 Division of Clinical Epidemiology and Aging Research, German Cancer Research Center (DKFZ), D-69120, Heidelberg, Germany; 59 German Cancer Consortium (DKTK), German Cancer Research Center (DKFZ), D-69120 Heidelberg, Germany; 60 Division of Preventive Oncology, German Cancer Research Center (DKFZ) and National Center for Tumor Diseases (NCT), Im Neuenheimer Feld 460, 69120 Heidelberg, Germany; 61 Humangenetik Tuebingen, Paul-Ehrlich-Str 23, D-72076 Tuebingen, Germany; 62 The University of Texas M. D. Anderson Cancer Center, Department of Genitourinary Medical Oncology, 1515 Holcombe Blvd., Houston, TX 77030, USA; 63 Departments of Epidemiology & Population Health and of Medicine, Division of Oncology, Stanford Cancer Institute, Stanford University School of Medicine, Stanford, CA 94304 USA; 64 Department of Genetics, Portuguese Oncology Institute of Porto (IPO-Porto), 4200-072 Porto, Portugal; 65 Biomedical Sciences Institute (ICBAS), University of Porto, 4050-313 Porto, Portugal; 66 Cancer Genetics Group, IPO-Porto Research Center (CI-IPOP), Portuguese Oncology Institute of Porto (IPO-Porto), 4200-072 Porto, Portugal; 67 Department of Population Sciences, Beckman Research Institute of City of Hope, 1500 East Duarte Road, Duarte, CA 91010, USA; 68 Ghent University, Faculty of Medicine and Health Sciences, Basic Medical Sciences, Proeftuinstraat 86, B-9000 Gent; 69 Department of Surgery, Faculty of Medicine, University of Malaya, 50603 Kuala Lumpur, Malaysia; 70 Department of Urology, University of Washington, 1959 NE Pacific Street, Box 356510, Seattle, WA 98195, USA; 71 Institute of Human Genetics, University Medical Center Hamburg-Eppendorf, D-20246 Hamburg, Germany; 72 Molecular Medicine Center, Department of Medical Chemistry and Biochemistry, Medical University of Sofia, Sofia, 2 Zdrave Str., 1431 Sofia, Bulgaria; 73 Department of Oncology, Cross Cancer Institute, University of Alberta, 11560 University Avenue, Edmonton, Alberta, Canada T6G 1Z2; 74 Division of Radiation Oncology, Cross Cancer Institute, 11560 University Avenue, Edmonton, Alberta, Canada T6G 1Z2; 75 Molecular Endocrinology Laboratory, Department of Cellular and Molecular Medicine, KU Leuven, BE-3000, Belgium; 76 Division of Cancer Sciences, Manchester Cancer Research Centre, Faculty of Biology, Medicine and Health, Manchester Academic Health Science Centre, NIHR Manchester Biomedical Research Centre, Health Innovation Manchester, Univeristy of Manchester, M13 9WL; 77 The University of Surrey, Guildford, Surrey, GU2 7XH, UK; 78 Genetic Oncology Unit, CHUVI Hospital, Complexo Hospitalario Universitario de Vigo, Instituto de Investigación Biomédica Galicia Sur (IISGS), 36204, Vigo (Pontevedra), Spain; 79 Department of Urology, Erasmus University Medical Center, 3015 CE Rotterdam, The Netherlands; 80 ”Exposome and Heredity”, CESP (UMR 1018), Faculté de Médecine, Université Paris-Saclay, Inserm, Gustave Roussy, Villejuif; 81 Clinical Gerontology Unit, University of Cambridge, Cambridge, CB2 2QQ, UK; 82 Division of Epidemiology, Department of Internal Medicine, University of Utah School of Medicine, Salt Lake City, Utah 84132, USA; 83 George E. Wahlen Department of Veterans Affairs Medical Center, Salt Lake City, Utah 84148, USA; 84 Department of Laboratory Medicine and Pathology, Mayo Clinic, Rochester, MN 55905, USA; 85 Nuffield Department of Population Health, University of Oxford, United Kingdom; 86 Program in Genetic Epidemiology and Statistical Genetics, Department of Epidemiology, Harvard School of Public Health, Boston, MA, USA; 87 Division of Epidemiology, Department of Medicine, Vanderbilt University Medical Center, 2525 West End Avenue, Suite 800, Nashville, TN 37232 USA; 88 International Epidemiology Institute, Rockville, MD 20850, USA; 89 Department of Epidemiology and Biostatistics, School of Public Health, Imperial College London, SW7 2AZ, UK; 1 Cancer Epidemiology Unit, Nuffield Department of Population Health, University of Oxford, Oxford, UK; 2 Genomic Epidemiology Branch, International Agency for Research on Cancer, Lyon, France; 3 Section of Nutrition and Metabolism, International Agency for Research on Cancer, Lyon, France; 4 Clinical Trial Service Unit and Epidemiological Studies Unit (CTSU), Nuffield Department of Population Health, University of Oxford, Oxford, UK; 5 Medical Research Council Population Health Research Unit at the University of Oxford, Oxford, UK; 6 Department of Population Health Sciences, Population Health Sciences, Bristol Medical School, University of Bristol, Bristol, UK; 7 MRC Integrative Epidemiology Unit (IEU), Population Health Sciences, Bristol Medical School, University of Bristol, Bristol, UK; 8 National Institute for Health Research (NIHR) Bristol Biomedical Research Centre, University Hospitals Bristol NHS Foundation Trust and Weston NHS Foundation Trust and the University of Bristol, Bristol, UK; 9 Department of Epidemiology and Biostatistics, School of Public Health, Imperial College London, London, UK; 10 Department of Hygiene and Epidemiology, University of Ioannina School of Medicine, Ioannina, Greece; 11 Division of Cancer Epidemiology and Genetics, National Cancer Institute, National Institutes of Health, Bethesda, Maryland, USA; 12 Navarra Public Health Institute, Pamplona, Spain; 13 Navarra Institute for Health Research (IdiSNA), Pamplona, Spain; 14 CIBER Epidemiology and Public Health CIBERESP, Madrid, Spain; 15 Centre for Nutrition, Prevention and Health Services, National Institute for Public Health and the Environment (RIVM), The Netherlands; 16 Program in Epidemiology, Division of Public Health Sciences, Fred Hutchinson Cancer Research Center, Seattle, Washington, USA; 17 Department of Epidemiology, School of Public Health, University of Washington, Seattle, Washington, USA; 18 Department of Otolaryngology: Head and Neck Surgery, School of Medicine, University of Washington, Seattle, Washington, USA; 19 Child Health and Development Studies, Public Health Institute, Berkeley, California, USA; 20 National Institute on Aging, Baltimore, Maryland, USA; 21 Medical School, University of Western Australia, Perth, Western Australia, Australia; 22 Western Australian Centre for Health and Ageing, University of Western Australia, Perth, Western Australia, Australia; 23 Cancer Epidemiology Division, Cancer Council Victoria, Melbourne, Victoria, Australia; 24 Centre for Epidemiology and Biostatistics, Melbourne School of Population and Global Health, The University of Melbourne, Melbourne, Victoria, Australia; 25 Precision Medicine, School of Clinical Sciences at Monash Health, Monash University, Melbourne, Victoria, Australia; 26 Department of Epidemiology, Harvard T.H. Chan School of Public Health, Boston, Massachusetts, USA; 27 Channing Division of Network Medicine, Brigham and Women's Hospital and Harvard Medical School, Boston, Massachusetts, USA; 28 Department of Nutrition, Harvard T.H. Chan School of Public Health, Boston, Massachusetts, USA; 29 Center for Genetic Epidemiology, Department of Preventive Medicine, Keck School of Medicine, University of Southern California/Norris Comprehensive Cancer Center, Los Angeles, California, USA; 30 National Clinical Research Center for Metabolic Diseases, Key Laboratory of Diabetes Immunology, Ministry of Education, and Department of Metabolism and Endocrinology, The Second Xiangya Hospital of Central South University, Changsha, Hunan, China; 31 Division of Cancer Epidemiology, German Cancer Research Center (DKFZ), Heidelberg, Germany; 32 Department of Public Health and Welfare, National Institute for Health and Welfare, Helsinki, Finland; 33 Department of Public Health and Health Policy, Graduate School of Biomedical and Health Sciences, Hiroshima University, Hiroshima, Japan; 34 Department of Research, Cancer Registry of Norway, Oslo, Norway; 35 Herbert Wertheim School of Public Health and Human Longevity Science, University of California San Diego, San Diego, California, USA; 36 University of Hawaii Cancer Center, Honolulu, Hawaii, USA; 37 Finnish Cancer Registry, Institute for Statistical and Epidemiological Cancer Research, Helsinki, Finland; 38 Department of Oncology, Helsinki University Central Hospital, Helsinki, Finland; 39 Department of Public Health and Welfare, Finnish Institute for Health and Welfare, Helsinki, Finland; 40 Department of Neurology, The University of Tennessee Health Science Center, College of Medicine, Memphis, Tennessee, USA; 41 Departmemt of Urology, Japanese Red Cross Kyoto Daiichi Hospital, Kyoto, Japan; 42 Department of Public Health, Aarhus University, Aarhus, Denmark; 43 Danish Cancer Society, Research Center, Copenhagen, Denmark; 44 Departmemt of Epidemiology, Radiation Effects Research Foundation, Hiroshima, Japan; 45 Cancer Risk Factors and Life-Style Epidemiology Unit, Institute for Cancer Research, Prevention and Clinical Network – ISPRO, Florence, Italy; 46 Department of Epidemiology, Johns Hopkins Bloomberg School of Public Health, Baltimore, Maryland, USA; 47 Epidemiology and Prevention Group, Center for Public Health Sciences, National Cancer Center, Tokyo, Japan; 48 Cancer Prevention Program, Public Health Sciences Division, Fred Hutchinson Cancer Research Center, Seattle, Washington, USA; 49 Department of Surgical Sciences, Uppsala University, Uppsala, Sweden; 50 Hokkaido University Faculty of Medicine, Sapporo, Japan; 51 Department of Medical Biosciences, Umeå University, Umeå, Sweden; 52 Department of Radiation Oncology, Memorial Sloan Kettering Cancer Center, New York, New York, USA; 53 Department of Public Health and Nursing, Faculty of Medicine, Norwegian University of Science and Technology, Trondheim, Norway; 54 Director Office, International Agency for Research on Cancer, World Health Organization, Lyon, France; 55 Department of Endocrinology and Diabetes, Fiona Stanley Hospital, Perth, Western Australia, Australia; 56 UK Biobank Ltd, Stockport, UK

**Keywords:** aggressive prostate cancer, Mendelian randomisation, prostate cancer, SHBG, testosterone

## Abstract

Previous studies had limited power to assess the associations of testosterone with aggressive disease as a primary endpoint. Further, the association of genetically predicted testosterone with aggressive disease is not known. We investigated the associations of calculated free and measured total testosterone and sex hormone-binding globulin (SHBG) with aggressive, overall and early-onset prostate cancer. In blood-based analyses, odds ratios (OR) and 95% confidence intervals (CI) for prostate cancer were estimated using conditional logistic regression from prospective analysis of biomarker concentrations in the Endogenous Hormones, Nutritional Biomarkers and Prostate Cancer Collaborative Group (up to 25 studies, 14 944 cases and 36 752 controls, including 1870 aggressive prostate cancers). In Mendelian randomisation (MR) analyses, using instruments identified using UK Biobank (up to 194 453 men) and outcome data from PRACTICAL (up to 79 148 cases and 61 106 controls, including 15 167 aggressive cancers), ORs were estimated using the inverse-variance weighted method. Free testosterone was associated with aggressive disease in MR analyses (OR per 1 SD = 1.23, 95% CI = 1.08-1.40). In blood-based analyses there was no association with aggressive disease overall, but there was heterogeneity by age at blood collection (OR for men aged <60 years 1.14, CI = 1.02-1.28; *P*_het_ = .0003: inverse association for older ages). Associations for free testosterone were positive for overall prostate cancer (MR: 1.20, 1.08-1.34; blood-based: 1.03, 1.01-1.05) and early-onset prostate cancer (MR: 1.37, 1.09-1.73; blood-based: 1.08, 0.98-1.19). SHBG and total testosterone were inversely associated with overall prostate cancer in blood-based analyses, with null associations in MR analysis. Our results support free testosterone, rather than total testosterone, in the development of prostate cancer, including aggressive subgroups.

## 1 Introduction

Prostate cancer is the second most common cancer in men worldwide and a leading cause of cancer death.^1^ Blood-based and genetic epidemiological studies show evidence of an association between circulating concentrations of calculated free testosterone and risk of overall prostate cancer.^2–6^ The association is biologically plausible because androgens are integral to the maintenance of prostate function.^7^ In the circulation, testosterone is bound to sex hormone-binding globulin (SHBG) and albumin. Approximately 2% of total testosterone circulates unbound or ‘free’, and according to the free hormone hypothesis is more biologically active.^8^ Prostate cancer varies in aggressiveness and tumours also vary by age of onset, and risk factors for these subgroups may be different from those for overall prostate cancer,^9^ but previous studies have lacked statistical power to assess associations of testosterone with prostate cancer subgroups.

The Endogenous Hormones, Nutritional Biomarkers and Prostate Cancer Collaborative Group (EHNBPCCG) is a pooled individual participant case-control dataset of prospective studies of risk of prostate cancer and associated risk factors. Previous analyses of the associations of circulating testosterone concentrations using the EHNBPCCG dataset were based on up to 6900 cases and 12 100 controls.2 We observed that men with very low free testosterone had a lower risk of overall prostate cancer, but we had limited power to investigate the associations with aggressive disease as a primary endpoint. This dataset has since been expanded to include more than double the number of prostate cancer cases, including 1900 aggressive and 600 early-onset cases.

Mendelian randomisation (MR) analyses, which use genetic instruments to predict average adult exposures, are less likely than blood-based studies to be affected by confounding factors or reverse causation, and are often considered to be a more reliable method for causal inference.^10^ Therefore, we carried-out two-sample MR analyses, using instruments identified from UK Biobank (up to 194 500 men) and genetic data from the PRACTICAL consortium (up to 79 000 prostate cancer cases [15 000 aggressive and 7000 early-onset subgroups] and 61 000 controls).^11,12^

Using these two international consortia, we aimed to extend our prior study in the EHNBPCCG to assess the associations of circulating concentrations of calculated free testosterone, as well as total testosterone and SHBG which are used to calculate free testosterone, with overall, aggressive and early-onset prostate cancer risk using blood-based and genetic methods; using these complementary approaches can provide more robust evidence for causal inference.

## 2 Materials and Methods

### 2.1 Endogenous Hormones, Nutritional Biomarkers and Prostate Cancer Collaborative Group

#### 2.1.1 Data collection and study designs

Individual participant data were available from up to 25 prospective studies with total testosterone and SHBG measurements. Participating studies are listed in Supplementary Table 1 and further details of data collection and processing are provided in the Supplementary Material (Appendix S2). Matching criteria are shown in Supplementary Table 2. Assay details and hormone measurement data are listed in Supplementary Table 3.

#### 2.1.2 Data processing and outcomes

Free testosterone concentrations were estimated using a formula based on the law of mass action from measured total testosterone and SHBG concentrations,^13,14^ assuming a constant albumin concentration of 43 g/L.

Disease definitions were as defined by the PRACTICAL consortium.^11,12^ Aggressive prostate cancer was categorised as ‘yes’ for any of the following: disease metastases at diagnosis (M1), Gleason score 8+ (or equivalent), prostate cancer death (defined as death from prostate cancer) or prostate-specific antigen (PSA) >100 ng/mL. Early-onset prostate cancer was defined as a diagnosis aged ≤55 years. Further details can be found in the Supplementary Methods (Appendix S2).

#### 2.1.3 Statistical analysis

Conditional logistic regression was used to estimate prostate cancer risk by free and total testosterone and SHBG concentrations. Analyses were conditioned on the study-specific matching variables and adjusted for age at blood collection, body mass index (BMI), height, smoking status, alcohol consumption, racial/ethnic group, education, married/cohabiting and diabetes status. Biomarkers were standardised by study and entered into the model as continuous variables, so each increment represents a 1 study-specific SD increase in biomarker concentration. For categorical analyses, biomarkers were categorised into study-specific fifths with cut-points determined in controls.^15^ Further details are available in the Supplementary Methods (Appendix S2).

#### 2.1.4 Further analyses

We examined heterogeneity in the associations of each biomarker with prostate cancer by participant characteristics and study (Supplementary Methods, Appendix S2). Subgroups were defined a priori based on the availability of data and previous analyses using this dataset.^2,5^ To further investigate the apparent heterogeneity by age at blood collection, we examined associations of free testosterone with overall and aggressive prostate cancer in fifths, stratified by age at blood collection (<60; 60+ years).

We also investigated associations in models conditioned on the matching variables but not further adjusted, associations in tenths, and estimates per 80th percentile increase. Associations were also examined following mutual adjustment for other biomarkers (including insulin-like growth factors [IGF-I, II] and IGF binding proteins [IGFBP-1,2,3]), and we tested for interactions between these biomarkers. Stratified analyses and associations in tenths were not investigated for early-onset disease due to the limited number of cases.

### 2.2 Mendelian randomisation analyses

#### 2.2.1 Genetic instruments for hormone concentrations

Summary GWAS results for circulating calculated free and total testosterone and SHBG for men in UK Biobank were extracted from a published analysis (based on up to 194 453 men of white European ancestry)^4^ (Supplementary Methods, Appendix S2). UK Biobank participants were aged 40-69 years at blood collection (mean age = 56.5 years). We pruned single nucleotide polymorphisms (SNPs) by a linkage disequilibrium threshold of r^2^ < .001.

#### 2.2.2 Genetic associations with prostate cancer

For each of the SNPs included as an instrument for free testosterone, total testosterone and SHBG, we obtained the association with prostate cancer risk from the PRACTICAL consortium (including GAME-ON/ELLIPSE).^11,12^ Individual studies included in these consortia are available from Schumacher et al.^11^ Associations with overall prostate cancer risk were generated from 79 148 prostate cancer cases and 61 106 controls, aggressive from 15 167 cases and 58 308 controls, and early-onset disease from 6988 cases and 44 256 controls.^11,12^ Genetic data for UK Biobank participants were not included in this dataset.

#### 2.2.3 Statistical analysis

The MR estimation for hormones was conducted using the inverse-variance weighted (IVW) method.^16^ We additionally calculated the I^2^ statistic to assess measurement error in SNP-exposure associations^17^ and Cochran’s Q statistic for heterogeneity between the MR estimates for each SNP.^18^ PhenoScanner was used to assess pleiotropy of the genetic instruments.^19^ As sensitivity analyses, we used the MR residual sum and outlier (MR-PRESSO) and MR robust adjusted profile score (MR-RAPS) to investigate the role of SNP outliers,^20^ and the weighted median, MR-Egger and the contamination mixture method to investigate horizontal pleiotropy.^21–23^

For SHBG, we additionally investigated associations of the *cis-*SNP with prostate cancer risk, as this cis-SNP may be less likely to be affected by horizontal pleiotropy than trans-SNPs.^24^ Associations of the cis-SNP with prostate cancer were assessed using the Wald ratio.

Details of statistical software and packages used are available in the Supplementary Methods (Appendix S2). All tests of significance were two-sided, and *P*-values <.05 were considered statistically significant.

## 3 Results

### 3.1 Study and participant characteristics in the blood-based analyses

A total of 25 studies, contributing up to 14 944 cases and 36 752 controls, were included in these analyses. Prostate cancer was classified as aggressive in 1870 cases and early-onset in 611 cases. Study participants were predominantly of white European ancestry (90%) (Table 1).

Prostate cancer characteristics by study are displayed in Supplementary Table 4. Mean age at blood collection for each study ranged from 33.8 to 76.8 years (overall mean = 61.0 years, SD = 8.5 years). Cases were diagnosed a mean of 6.5 years (SD = 5.9) after blood collection, and the mean age at diagnosis was 67.3 years (SD = 6.7) (Table 1). Partial correlations between biomarkers ranged from *r* = −0.04 (SHBG and PSA) to *r* = 0.77 (calculated free and total testosterone) (Supplementary Table 5).

### 3.2 Free testosterone

**Figure 1:**
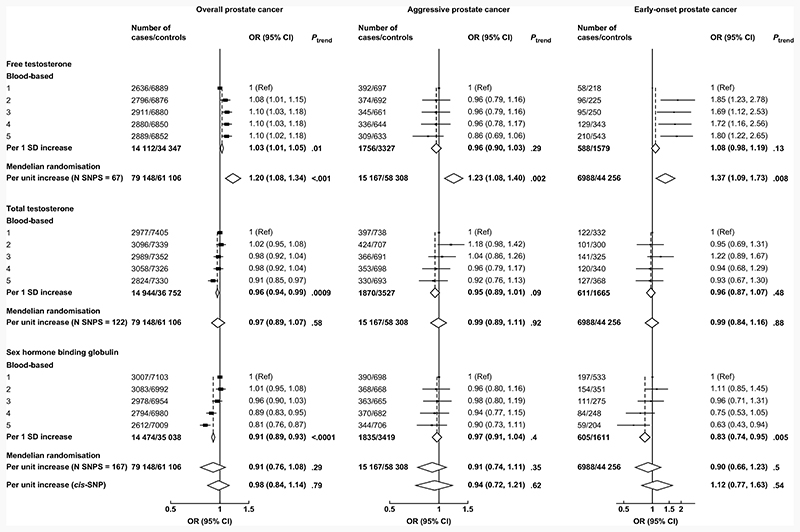
Risks of overall, aggressive* and early-onset^†^ prostate cancer in by study-specific fifths of hormone concentrations (blood-based only) and unit increment (blood-based and MR). Blood-based estimates are from logistic regression conditioned on the matching variables and adjusted for age, BMI, height, alcohol intake, smoking status, marital status, education status, racial/ethnic group and diabetes status. The position of each square indicates the magnitude of the odds ratio, and the area of the square is proportional to the inverse of the variance of the logarithm of the OR. The length of the horizontal line through the square indicates the 95% CI. MR risk estimates are estimated using the inverse variance weighted method for the full instrument methods and the Wald ratio in the cis-SNP analyses (where applicable). In MR analyses, biomarker transformations are outlined in the Supplementary Methods (Appendix S2). *Aggressive cancer defined as Gleason grade 8+, or prostate cancer death or metastases or PSA >100 ng/mL. ^†^Early-onset defined as diagnosed ≤55 years. BMI, body mass index; CI, confidence interval; OR, odds ratio; PSA, prostate-specific antigen; SNP, single nucleotide polymorphism

The association of calculated free testosterone with overall prostate cancer risk was significant in both blood-based (OR per 1 SD increment = 1.03, 95% CI 1.01-1.05) and MR analyses (OR per genetically predicted 1 SD increment = 1.20, 1.08-1.34) (Figure 1). Higher free testosterone was associated with a higher risk of aggressive prostate cancer in the MR analysis (1.23, 1.08-1.40), but there was no evidence of an association in the blood-based analysis (0.96, 0.90-1.03) (Figure 1). MR sensitivity analyses generally supported the associations of free testosterone with overall and aggressive prostate cancer, except for MR-Egger,although the MR-Egger intercepts did not indicate directional pleiotropy (Table 2).

In the MR analysis, predicted free testosterone was associated with an increased risk of early-onset disease (1.37, 1.09-1.73), and the relationship was directionally consistent in blood-based analyses (1.08, 0.98-1.19) (Figure 1). The associations with early-onset disease were less robust in the MR sensitivity analyses but were directionally consistent (Table 2).

### 3.3 Total testosterone

The OR for total testosterone in relation to overall prostate cancer was 0.96 (0.94-0.99) in blood-based analysis and 0.97 (0.89-1.07) in MR analysis (Figure 1 and Table 2). Total testosterone was not associated with aggressive or early-onset disease in blood-based or in the MR analyses (Figure 1 and Table 2).

### 3.4 Sex hormone-binding globulin

SHBG was inversely associated with overall and early-onset prostate cancer in blood-based analyses (0.91, 0.89-0.93 and 0.83, 0.74-0.95, respectively), but was not associated with aggressive disease risk (0.97, 0.91-1.04) (Figure 1). In the MR analyses, SHBG was not associated with prostate cancer risk using the full instrument or the *cis*-SNP instrument (Figure 1 and Table 2).

#### 3.4.1 Further analyses – blood-based analysis

**Figure 2:**
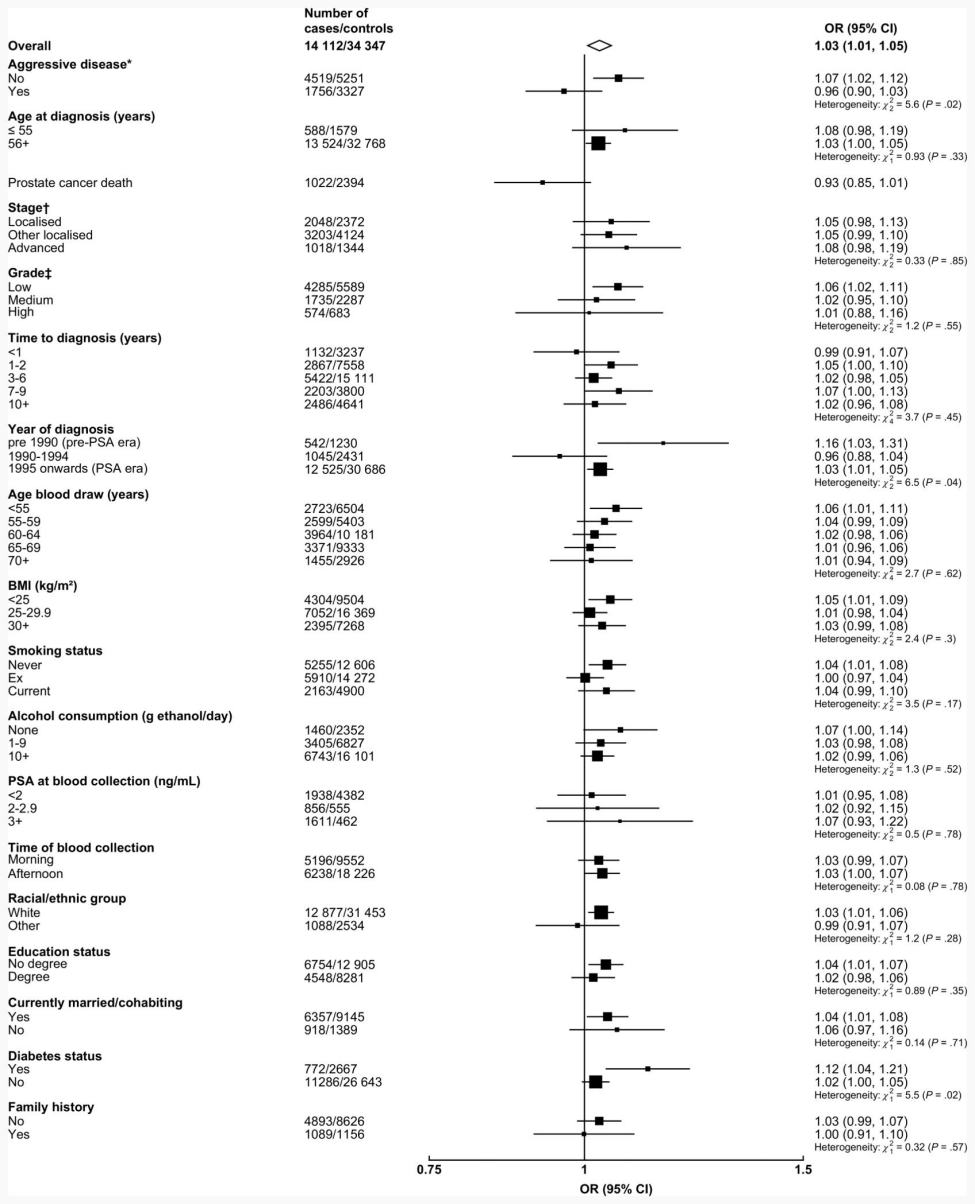
Odds ratio (95% CIs) for overall prostate cancer per study-specific 1 SD increment of free testosterone concentration by subgroup. Estimates are from logistic regression conditioned on the matching variables and adjusted for age, BMI, height, alcohol intake, smoking status, marital status, education status, racial/ethnic group and diabetes status. The position of each square indicates the magnitude of the OR, and the area of the square is proportional to the inverse of the variance of the logarithm of the OR). The length of the horizontal line through the square indicates the 95% CI. Tests for heterogeneity for case-defined factors were obtained by fitting separate models for each subgroup and assuming independence of the ORs using a method analogous to a metaanalysis. Tests for heterogeneity for non-case-defined factors were assessed with a *χ*^2^ test of interaction between subgroup and the binary variable. *Aggressive cancer defined as Gleason grade 8+, or prostate cancer death or metastases or PSA >100 ng/mL. ^†^Localised defined as TNM stage <T2 with no reported lymph node involvement or metastases or stage I; other localised stage if TNM stage T2 with no reported lymph node involvement or metastases, stage II or equivalent; advanced stage if they were TNM stage T3 or T4 and/or N1+ and/or M1, stage III-IV or equivalent. ^‡^Low grade defined as Gleason score was <7 or equivalent (ie, extent of differentiation good, moderate); medium grade if Gleason score was 7 (ie, poorly differentiated); high grade if the Gleason score was ≥8 or equivalent (ie, undifferentiated). BMI, body mass index; CI, confidence interval; OR, odds ratio; PSA, prostate-specific antigen

There was significant heterogeneity in the associations of free testosterone with risk according to the prostate cancer aggressiveness; higher free testosterone concentration was associated with an increased risk of nonaggressive (1.07, 1.02-1.12), but not aggressive disease (0.96, 0.90-1.03; *P*_het_ = .02) (Figure 2). Men with diabetes also had a larger magnitude of association of free testosterone with overall prostate cancer than men without diabetes (1.12, 1.04-1.21 and 1.02, 1.00-1.05, respectively; *P*_het_ = .02) (Figure 2).

**Figure 3:**
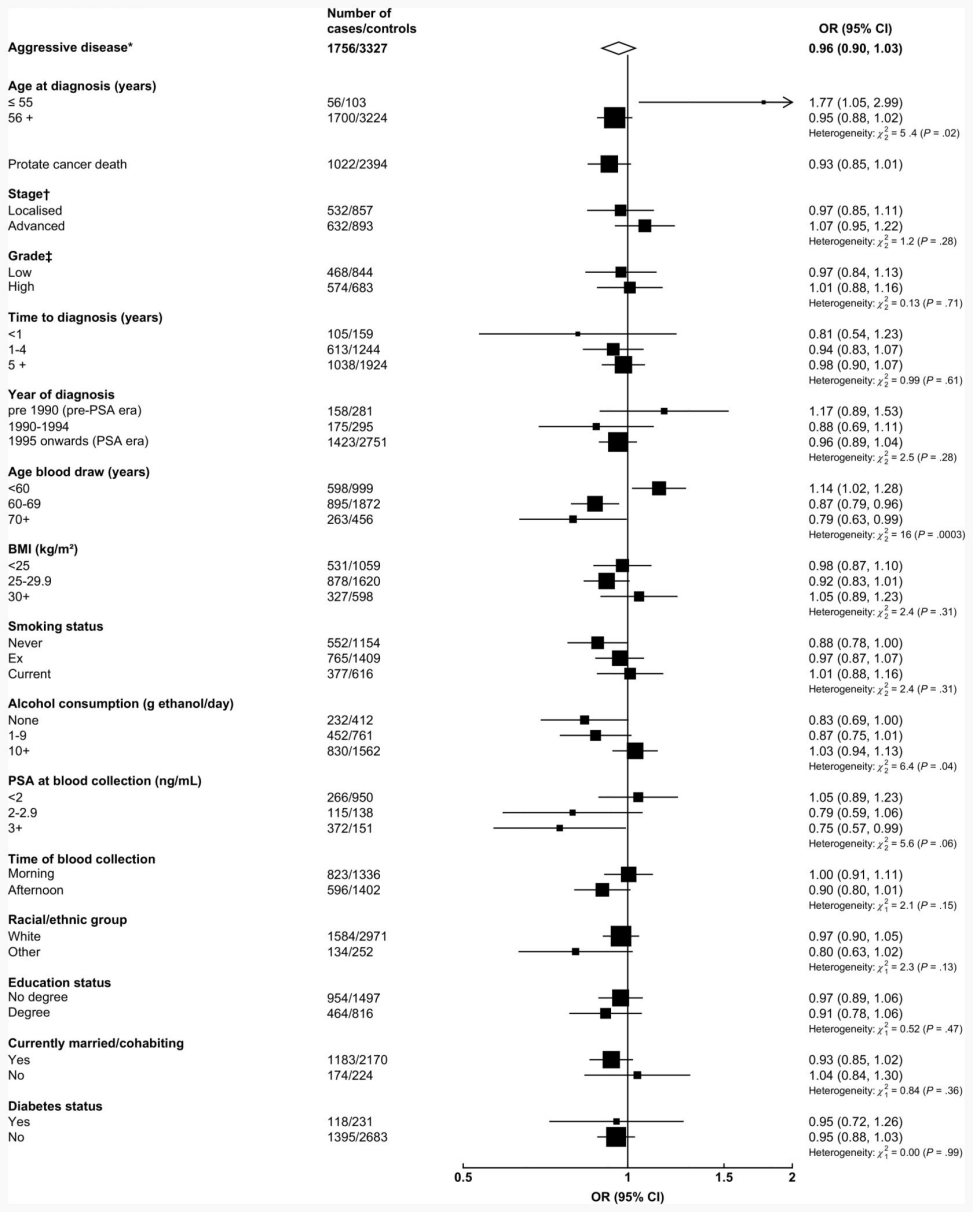
Odds ratio (95% CIs) for aggressive* prostate cancer per study-specific 1 SD increment of free testosterone concentration by subgroup. Estimates are from logistic regression conditioned on the matching variables and adjusted for age, BMI, height, alcohol intake, smoking status, marital status, education status, racial/ethnic group and diabetes status. The position of each square indicates the magnitude of the OR, and the area of the square is proportional to theinverse of the variance of the logarithm of the OR). The length of the horizontal line through the square indicates the 95% CI. Tests for heterogeneity for case-defined factors were obtained by fitting separate models for each subgroup and assuming independence of the ORs using a method analogous to a metaanalysis. Tests for heterogeneity for non-case-defined factors were assessed with a*χ^2^* test of interaction between subgroup and the binary variable. *Aggressive cancer defined as Gleason grade 8+, or prostate cancer death, or metastases or PSA >100 ng/mL. ^†^ Localised defined as TNM stage <T2 with no reported lymph node involvement or metastases or stage I, or TNM stage T2 with no reported lymph node involvement or metastases, stage II, or equivalent; advanced stage if they were TNM stage T3 or T4 and/or N1+ and/or M1, stage III-IV or equivalent. ^‡^Low grade defined as Gleason score was <8 or equivalent (ie, extent of differentiation good, moderate, poor); high grade if the Gleason score was ≥8 or equivalent (ie, undifferentiated). BMI, body mass index; CI, confidence interval; OR, odds ratio; PSA, prostate-specific antigen

For aggressive disease risk, there was significant heterogeneity in the associations by age at blood collection; free testosterone was positively associated with aggressive prostate cancer (1.14, 1.02-1.28) for men whose blood was collected at ages <60 years, but the relationship was inverse for men whose blood was collected at older ages (0.87, 0.79-0.96 and 0.79, 0.63-0.99 for men whose blood was collected aged 60-69 and 70+ years, respectively) (P_het_ = .0003) (Figure 3). In analyses based on fifths of free testosterone, there was a positive dose-response relationship of free testosterone with overall and aggressive prostate cancer for men whose blood was collected at <60 years, while for men whose blood was collected at an older age, the relationship was null with overall prostate cancer and inverse with aggressive prostate cancer (Supplementary Figure 1). Higher free testosterone was also associated with an elevated risk of early-onset aggressive disease (1.77, 1.05-2.99) but was not associated with aggressive disease for men diagnosed later in life (0.95, 0.88-1.02; *P*_het_ = .02) (Figure 3), although there was a small number of cases of early-onset aggressive disease (n = 56).

The associations of total testosterone and SHBG with overall and aggressive prostate cancer were generally consistent by subgroups (Supplementary Figures 2-5). Total testosterone was inversely associated with prostate cancer death (0.90, 0.82-0.97) and positively associated with early-onset aggressive prostate cancer (2.40, 1.28-4.52), while for men diagnosed with aggressive disease aged >55 years the OR was 0.94 (0.88-1.00; *P*_het_ = .0004) (Supplementary Figure 3).

There was no statistically significant heterogeneity in the associations with overall and aggressive prostate cancer by study (Supplementary Figures 6-11), except for free testosterone and aggressive prostate cancer (*P*_het_ = .02) (Supplementary Figure 7). Associations were broadly similar in unadjusted matched analyses (Supplementary Figure 12), study-specific tenths (Supplementary Figure 13), per 80%tile increase (Supplementary Table 6) and following mutual adjustment for other biomarkers (Supplementary Table 7).

There were significant interactions in the associations of total testosterone with overall and aggressive prostate cancer by SHBG concentrations (Supplementary Tables 8 and 9). SHBG was positively associated with aggressive disease risk for men with lower IGFBP-1 concentrations (1.23, 1.00-1.51), and the relationship was inverse for men with higher IGFBP-1 concentrations (0.85, 0.71-1.02; *P*_het_ = .01) (Supplementary Table 9).

#### 3.4.2 Further analyses – Mendelian randomisation

There was no strong evidence of measurement error in the genetic instruments for the biomarkers (*I*^2^ > 0.96). There was significant heterogeneity in the MR estimates for the SNPs with overall disease, and for aggressive and early-onset disease (Cochran’s Q *P* < .001), except for the association of free testosterone with aggressive disease (*P* = .12). Using PhenoScanner, 175, 355 and 358 traits were linked to SNPs for free testosterone, SHBG and total testosterone concentrations, respectively, particularly adiposity and height, and SNPs associated with free testosterone were frequently related to age at puberty (*P* < 5 x 10^−8^) (Supplementary Figures 14-16). Traits linked to the SHBG *cis*-SNP (rs1799941) are shown in Supplementary Table 10. MR scatterplots and tables are found in Supplementary Figures 17-19 and Supplementary Tables 11-13.

## 4 Discussion

In this first comprehensive analysis with both blood-based and genetic data, our results suggest that higher calculated free testosterone is associated with an elevated risk for prostate cancer, including aggressive disease. Neither circulating total testosterone nor SHBG was associated with elevated risks for prostate cancer.

The strong genetic evidence in our MR analyses (which are less likely to be affected by biases such as confounding, reverse causation and detection bias) for a role of free testosterone, alongside the well-characterised lower risk of prostate cancer in men diagnosed with Klinefelter’s syndrome^25^ (a genetic abnormality which is characterised by life-long clinically low total and free testosterone concentrations^26^), indicates a probable causal relationship of free testosterone with prostate cancer, including with aggressive disease. While in our blood-based analyses the overall association of free testosterone with aggressive prostate cancer was null, there was evidence of a positive association with aggressive disease for men whose blood who was collected at a younger age. However, we observed inverse associations of free testosterone with aggressive disease for men whose blood was collected at an older age, which warrants further consideration. Differences between the associations of genetically predicted free testosterone and measured blood concentrations with prostate cancer risk may implicate the importance of free testosterone concentrations in younger adulthood. Free testosterone concentrations decline with older age, partly due to cumulative environmental influences, therefore free testosterone concentrations in middle and older age may not be representative of life-long exposure to free testosterone concentrations, which will attenuate risk estimates.^27–32^ There was also some evidence of heterogeneity in the blood-based association of free testosterone with aggressive disease by study, which may relate to differences in participant and tumour characteristics.

As well as the blood-based and genetic evidence that we describe here, two randomised controlled trials using 5**α**-reductase inhibitors, which aimed to reduce intraprostatic androgen signalling by reducing dihydrotestosterone concentrations by 80-90%,^33^ have reported 23-25% lower risks of overall prostate cancer. However, these trials also reported 27-58% increased risks of high-grade tumours,^34,35^ possibly due to changes in prostate morphology, function biasing tumour diagnostic grading, and/or the early development of partial androgen insensitivity in more aggressive tumours (in comparison with low-grade tumours)^36,37^; long-term follow-up of these trials does not support an effect on risk of prostate cancer mortality.^38^

For total testosterone and SHBG the MR results were null suggesting no direct effect, whereas the blood-based analyses were inverse for both; it is possible that the inverse results for testosterone are due to reverse causation, but results did not suggest this for SHBG and the explanation for the blood-based results remains unclear.

These analyses have several strengths. This is the largest collection of prospective blood-based and genetic data on sex hormones and prostate cancer risk available, representing almost all the available data worldwide. This large sample size maximised power to assess associations robustly and enabled us to investigate associations across subgroups. Further, by incorporating blood-based and MR methods we were able to use different lines of evidence to inform causal inference.^39^

Limitations include that we used calculated rather than directly measured free testosterone concentrations using equilibrium dialysis,^40^ we have used a validated formula to estimate concentrations and these are well correlated.^41,42^ It has also been suggested that the bioavailable fraction of testosterone is the sum of free and albumin-bound testosterone rather than solely the free fraction,^43^ but it is not possible in our data to distinguish between these hypotheses because estimates of these fractions from the formula are perfectly correlated. Furthermore, the predictive value of peripheral free testosterone as an indicator of intraprostatic signalling remains under debate.^44^ Our analyses relied on single biomarker measurements, and although these biomarkers have good reproducibility over a 4-to-5-year period (intraclass correlation coefficients 0.54-0.82),2 longitudinal studies have shown that free testosterone declines continually throughout adulthood^45^; this may lead to underestimates of risk.^46^ Participants in the EHNBPCCG dataset were predominantly white and therefore we were underpowered to investigate associations for other racial/ethnic groups. Prospective epidemiological studies were generally based on older men, therefore we had more limited power to investigate associations in younger participants.

In the MR analyses of free testosterone, we observed weaker relationships using MR-Egger. MR-Egger is less susceptible to confounding from possibly pleiotropic variants that have stronger effects on the outcome than the exposure. However, this approach is also subject to reduced power and therefore does not necessarily imply the absence of a causal effect in the context of consistent sensitivity analyses and balanced pleiotropy.^21,47^ Further, testosterone is a steroid and therefore no cis-genetic instruments are available. These limitations mean that we cannot exclude the possibility that the MR results for free testosterone may be influenced by some horizontal pleiotropy. It is also not known whether the performance of the genetic predictors of free testosterone change with age.^48^ Future genetic and blood-based research including younger men with repeat measurements and linkage to detailed medical records will help to clarify associations.

The blood-based results we report here are an extension of our previous paper,2 and includes more than double the number of cases, with the incorporation studies including UK Biobank and extended follow-up from some other studies. Our blood-based results indicated possible nonlinear relationships with overall prostate cancer (as reported previously)^2^ and with early-onset prostate cancer, with lower risks of overall and early-onset prostate cancer for men with low free testosterone concentrations. For MR analyses, genetic instruments were based on summary GWAS results, and we were therefore unable to investigate possible nonlinear associations. For overall prostate cancer we also previously reported a possible increased risk of high-grade disease; however, we have limited additional data for grade, and therefore we do not include an updated detailed grade analysis as reported in the previous paper.

In conclusion, the findings from these blood-based and genetic analyses implicate free testosterone in the development of prostate cancer, including aggressive and early-onset disease.

## Supplementary Material

Appendix 1

Supplementary information

## Figures and Tables

**Table 1:** Characteristics of prostate cancer cases and controls in the EHNBPCCG participants

	Controls	Cases		
		Overall	Aggressive^a^	Early-onset^b^
N	36 752	14 944	1870	611
Age at blood collection (yr), mean (SD)	61.0 (8.4)	60.8 (8.6)	62.1 (8.4)	46.7 (5.7)
Height (cm), mean (SD)	174.6 (7.2)	174.7 (7.3)	173.7 (7.8)	177.1 (6.9)
BMI (kg/m^2^), mean (SD)	27.4 (4.1)	26.9 (3.7)	27.1 (4.0)	26.5 (3.7)
PSA at blood collection (ng/mL), med (IQR)	0.9 (1.2)	2.3 (3.2)	3.0 (5.6)	1.6 (2.5)
Time from blood collection to diagnosis, mean (SD)	-	6.5 (5.9)	7.5 (7.1)	5.8 (5.3)
Age at diagnosis, mean (SD)	-	67.3 (6.7)	67.0 (6.2)	52.5 (2.5)
Racial/ethnic group, N (%)				
White	33 645 (91.5)	13 586 (90.9)	1676 (89.6)	559 (91.5)
Black	1222 (3.3)	524 (3.5)	57 (3.0)	31(5.1)
East Asian	875 (2.4)	484 (3.2)	89 (4.8)	3 (0.5)
Other	678 (1.8)	236 (1.6)	17 (0.9)	10 (1.6)
Not known	332 (0.9)	114 (0.8)	31 (1.7)	8(1.3)
Smoking status, N (%)				
Never	13 868 (37.7)	5681 (38.0)	599 (32.0)	273 (44.7)
Ex	15 548 (42.3)	6329 (42.4)	815 (43.6)	160 (26.2)
Current	5674 (15.4)	2351 (15.7)	407 (21.8)	140 (22.9)
Not known	1662 (4.5)	583 (3.9)	49 (2.6)	38 (6.2)
Alcohol consumption (g ethanol/day), N (%)				
Nondrinker	2673 (7.3)	1615 (10.8)	250 (13.4)	46 (7.5)
<10	8189 (22.3)	3752 (25.1)	484 (25.9)	140 (22.9)
10+	19 198 (52.2)	7309 (48.9)	883 (47.2)	300 (49.1)
Not known	6692 (18.2)	2268 (15.2)	253 (13.5)	125 (20.5)
Diabetes status, N (%)				
Yes	2887 (7.9)	819 (5.5)	122 (6.5)	13 (2.1)
No	28 745 (78.2)	11 913 (79.7)	1487 (79.5)	467 (76.4)
Not known	5120 (13.9)	2212 (14.8)	261 (14.0)	131 (21.4)
Married/cohabiting, N (%)				
Yes	9767 (26.6)	6790 (45.4)	1295 (69.3)	222 (36.3)
No	1461 (4.0)	958 (6.4)	183 (9.8)	39 (6.4)
Not known	25 524 (69.4)	7196 (48.2)	392 (21.0)	350 (57.3)

*Note:* Some aggressive disease characterisation data were available from 88% of included studies.

Abbreviations: BMI, body mass index; IQR, interquartile range; PSA, prostate-specific antigen.

a Aggressive disease was defined as Gleason Score 8+, death from prostate cancer, metastatic disease or PSA >100 ng/mL.

b Early-onset defined as diagnosed aged ≤55 years.

**Table 2:** Mendelian randomisation estimates between genetically predicted circulating biomarker concentrations and prostate cancer risk

	Variance explained	NSNPs	Overall prostate cancer (79 148 cases, 61 106 controls)		Aggressive prostate cancer^a^ (15 167 cases, 58 308 controls)		Early-onset prostate cancer^b^ (6988 cases, 44 256 controls)	
			OR per unit increment in biomarker (95% CI)	*P*	OR per unit increment in biomarker (95% CI)	P	OR per unit increment in biomarker (95% CI)	P
Free testosterone (SD = 59.5 pmol/L)								
Inverse-variance weighted	3.8%	67	1.20 (1.08, 1.34)	0.0006	1.23 (1.08, 1.40)	0.002	1.37 (1.09, 1.73)	0.008
Weighted median			1.12 (1.01, 1.25)	0.04	1.19 (0.99, 1.43)	0.07	1.16 (0.89, 1.52)	0.27
MR-Egger			1.07 (0.87, 1.31)	0.53	1.03 (0.80, 1.32)	0.84	1.09 (0.69, 1.72)	0.71
MR-Egger intercept				0.20		0.11		0.26
MR-RAPS			1.16 (1.05, 1.28)	0.002	1.20 (1.05, 1.36)	0.01	1.33 (1.05, 1.67)	0.02
MR-PRESSO			1.13 (1.05, 1.22)	0.002	1.23 (1.08, 1.40)^c^	0.002	1.33 (1.07, 1.65)	0.01
Contamination mixture			1.12 (1.04, 1.22)	0.007	1.20 (1.00, 1.39)	0.05	1.22 (0.94, 1.97)	0.14
Total testosterone (SD = 3.8 nmol/L)								
Inverse-variance weighted	7.5%	122	0.97 (0.89, 1.07)	0.58	0.99 (0.89, 1.11)	0.92	0.99 (0.84, 1.16)	0.88
Weighted median			0.99 (0.91, 1.08)	0.89	0.99 (0.86, 1.14)	0.84	1.07 (0.86, 1.32)	0.53
MR-Egger			0.99 (0.85, 1.15)	0.92	1.06 (0.89, 1.26)	0.53	0.95 (0.73, 1.25)	0.73
MR-Egger intercept				0.77		0.39		0.76
MR-RAPS			1.04 (0.94, 1.14)	0.45	1.03 (0.93, 1.14)	0.62	1.00 (0.85, 1.18)	0.99
MR-PRESSO			1.02 (0.95, 1.09)	0.60	0.92 (0.79, 1.08)	0.30	1.01 (0.88, 1.17)	0.88
Contamination mixture			1.06 (0.99, 1.17)	0.09	1.02 (0.94, 1.14)	0.54	1.05 (0.86, 1.21)	0.72
SHBG (SD = 16.5 nmol/L)								
Inverse-variance weighted	15.0%	168	0.91 (0.76, 1.08)	0.29	0.91 (0.74, 1.11)	0.35	0.90 (0.66, 1.23)	0.50
Weighted median			0.98 (0.85, 1.13)	0.79	0.94 (0.74, 1.18)	0.58	1.12 (0.79, 1.58)	0.52
MR-Egger			0.99 (0.76, 1.27)	0.92	1.05 (0.78, 1.40)	0.76	0.97 (0.62, 1.52)	0.89
MR-Egger intercept				0.38		0.18		0.64
MR-RAPS			1.01 (0.86, 1.19)	0.87	0.95 (0.79, 1.15)	0.60	0.93 (0.70, 1.25)	0.63
MR-PRESSO			0.98 (0.88, 1.10)	0.76	0.93 (0.79, 1.09)	0.36	0.98 (0.77, 1.24)	0.86
Contamination mixture			0.96 (0.86, 1.07)	0.55	0.90 (0.77, 1.05)	0.21	1.01 (0.70, 1.30)	0.92
*cis*-SNP (rs1799941)	4.2%	1	0.98 (0.84, 1.14)	0.79	0.94 (0.72, 1.21)	0.62	1.12 (0.77, 1.63)	0.54

*Note*: Biomarker transformations are outlined in the Supplementary Methods (Appendix S2).

Abbreviations: CI, confidence interval; MR, Mendelian randomisation; OR, odds ratio; PRESSO, pleiotropy residual sum and outlier; RAPS, robust adjusted profile score; SHBG, sex hormone-binding globulin.

a Aggressive disease was defined as Gleason Score 8+, death from prostate cancer, metastatic disease or PSA >100 ng/mL.

b Early-onset defined as diagnosed aged ≤55 years.

c No statistically significant outliers detected.

## Data Availability

For prospective analysis, the authors do not own the rights for the data contained in the EHNBPCCG dataset and therefore cannot redistribute the data. However, researchers can contact individual studies for access requests. UK Biobank individual-level data are available upon request, while summary genetic data are publicly available (www.ukbiobank.ac.uk). PRACTICAL genetic data for overall prostate cancer are publicly available, while genetic subgroup data are available upon request (http://practical.icr.ac.uk/). Further details and other data that support the findings of our study are available from the corresponding author upon request.

## What’s new?

The Endogenous Hormones, Nutritional Biomarkers and Prostate Cancer Collaborative Group (EHNBPCCG) is a pooled dataset of prospective studies of prostate cancer risk. Using this data, the authors conducted blood-based analysis and Mendelian randomisation analysis to determine the association between circulating testosterone and overall risk of prostate cancer, as well as looking at risk of aggressive disease and early-onset cancer separately. They found strong evidence that higher concentrations of circulating testosterone increases the risk of prostate cancer, including aggressive subtypes. This is the largest collection of prospective blood-based observational and genetic data on sex hormones and prostate cancer risk to date.

## Abbreviations

BMI: body mass index
CI: confidence interval
EHNBPCCG: The Endogenous Hormones, Nutritional Biomarkers and Prostate Cancer Collaborative Group
IGF: insulin-like growth factor
IGFBP: insulin-like growth factor binding protein
MR: Mendelian randomisation
MR-PRESSO: MR residual sum and outlier
RAPS: MR-MR robustadjusted profile score
OR: odds ratio
PRACTICAL: Prostate Cancer Association Group to Investigate CancerAssociated Alterations in the Genome
PSA: prostate-specific antigen
SHBG: sex hormone binding globulin.
