# Circulating free testosterone and risk of aggressive prostate cancer: Prospective and Mendelian randomisation analyses in international consortia

**DOI:** 10.1002/ijc.34116

**Published:** 2022-06-07

**Authors:** Eleanor L. Watts, Aurora Perez‐Cornago, Georgina K. Fensom, Karl Smith‐Byrne, Urwah Noor, Colm D. Andrews, Marc J. Gunter, Michael V. Holmes, Richard M. Martin, Konstantinos K. Tsilidis, Demetrius Albanes, Aurelio Barricarte, Bas Bueno‐de‐Mesquita, Chu Chen, Barbara A. Cohn, Niki L. Dimou, Luigi Ferrucci, Leon Flicker, Neal D. Freedman, Graham G. Giles, Edward L. Giovannucci, Gary E. Goodman, Christopher A. Haiman, Graeme J. Hankey, Jiaqi Huang, Wen‐Yi Huang, Lauren M. Hurwitz, Rudolf Kaaks, Paul Knekt, Tatsuhiko Kubo, Hilde Langseth, Gail Laughlin, Loic Le Marchand, Tapio Luostarinen, Robert J. MacInnis, Hanna O. Mäenpää, Satu Männistö, E. Jeffrey Metter, Kazuya Mikami, Lorelei A. Mucci, Anja W. Olsen, Kotaro Ozasa, Domenico Palli, Kathryn L. Penney, Elizabeth A. Platz, Harri Rissanen, Norie Sawada, Jeannette M. Schenk, Pär Stattin, Akiko Tamakoshi, Elin Thysell, Chiaojung Jillian Tsai, Shoichiro Tsugane, Lars Vatten, Elisabete Weiderpass, Stephanie J. Weinstein, Lynne R. Wilkens, Bu B. Yeap, The PRACTICAL Consortium, CRUK, BPC3, CAPS, PEGASUS, Naomi E. Allen, Timothy J. Key, Ruth C. Travis

**Affiliations:** ^1^ Cancer Epidemiology Unit, Nuffield Department of Population Health University of Oxford Oxford UK; ^2^ Genomic Epidemiology Branch International Agency for Research on Cancer Lyon France; ^3^ Section of Nutrition and Metabolism International Agency for Research on Cancer Lyon France; ^4^ Clinical Trial Service Unit and Epidemiological Studies Unit (CTSU), Nuffield Department of Population Health University of Oxford Oxford UK; ^5^ Medical Research Council Population Health Research Unit at the University of Oxford Oxford UK; ^6^ Department of Population Health Sciences, Population Health Sciences, Bristol Medical School University of Bristol Bristol UK; ^7^ MRC Integrative Epidemiology Unit (IEU), Population Health Sciences, Bristol Medical School University of Bristol Bristol UK; ^8^ National Institute for Health Research (NIHR) Bristol Biomedical Research Centre University Hospitals Bristol NHS Foundation Trust and Weston NHS Foundation Trust and the University of Bristol Bristol UK; ^9^ Department of Epidemiology and Biostatistics, School of Public Health Imperial College London London UK; ^10^ Department of Hygiene and Epidemiology University of Ioannina School of Medicine Ioannina Greece; ^11^ Division of Cancer Epidemiology and Genetics, National Cancer Institute National Institutes of Health Bethesda Maryland USA; ^12^ Navarra Public Health Institute Pamplona Spain; ^13^ Navarra Institute for Health Research (IdiSNA) Pamplona Spain; ^14^ CIBER Epidemiology and Public Health CIBERESP Madrid Spain; ^15^ Centre for Nutrition, Prevention and Health Services National Institute for Public Health and the Environment (RIVM) The Netherlands; ^16^ Program in Epidemiology, Division of Public Health Sciences Fred Hutchinson Cancer Research Center Seattle Washington USA; ^17^ Department of Epidemiology, School of Public Health University of Washington Seattle Washington USA; ^18^ Department of Otolaryngology: Head and Neck Surgery, School of Medicine University of Washington Seattle Washington USA; ^19^ Child Health and Development Studies Public Health Institute Berkeley California USA; ^20^ National Institute on Aging Baltimore Maryland USA; ^21^ Medical School University of Western Australia Perth Western Australia Australia; ^22^ Western Australian Centre for Health and Ageing University of Western Australia Perth Western Australia Australia; ^23^ Cancer Epidemiology Division Cancer Council Victoria Melbourne Victoria Australia; ^24^ Centre for Epidemiology and Biostatistics, Melbourne School of Population and Global Health The University of Melbourne Melbourne Victoria Australia; ^25^ Precision Medicine, School of Clinical Sciences at Monash Health Monash University Melbourne Victoria Australia; ^26^ Department of Epidemiology Harvard T.H. Chan School of Public Health Boston Massachusetts USA; ^27^ Channing Division of Network Medicine Brigham and Women's Hospital and Harvard Medical School Boston Massachusetts USA; ^28^ Department of Nutrition Harvard T.H. Chan School of Public Health Boston Massachusetts USA; ^29^ Center for Genetic Epidemiology, Department of Preventive Medicine, Keck School of Medicine University of Southern California/Norris Comprehensive Cancer Center Los Angeles California USA; ^30^ National Clinical Research Center for Metabolic Diseases, Key Laboratory of Diabetes Immunology, Ministry of Education, and Department of Metabolism and Endocrinology The Second Xiangya Hospital of Central South University Changsha Hunan China; ^31^ Division of Cancer Epidemiology German Cancer Research Center (DKFZ) Heidelberg Germany; ^32^ Department of Public Health and Welfare National Institute for Health and Welfare Helsinki Finland; ^33^ Department of Public Health and Health Policy, Graduate School of Biomedical and Health Sciences Hiroshima University Hiroshima Japan; ^34^ Department of Research Cancer Registry of Norway Oslo Norway; ^35^ Herbert Wertheim School of Public Health and Human Longevity Science University of California San Diego San Diego California USA; ^36^ University of Hawaii Cancer Center Honolulu Hawaii USA; ^37^ Finnish Cancer Registry Institute for Statistical and Epidemiological Cancer Research Helsinki Finland; ^38^ Department of Oncology Helsinki University Central Hospital Helsinki Finland; ^39^ Department of Public Health and Welfare Finnish Institute for Health and Welfare Helsinki Finland; ^40^ Department of Neurology The University of Tennessee Health Science Center, College of Medicine Memphis Tennessee USA; ^41^ Departmemt of Urology Japanese Red Cross Kyoto Daiichi Hospital Kyoto Japan; ^42^ Department of Public Health Aarhus University Aarhus Denmark; ^43^ Danish Cancer Society Research Center Copenhagen Denmark; ^44^ Departmemt of Epidemiology Radiation Effects Research Foundation Hiroshima Japan; ^45^ Cancer Risk Factors and Life‐Style Epidemiology Unit, Institute for Cancer Research Prevention and Clinical Network – ISPRO Florence Italy; ^46^ Department of Epidemiology Johns Hopkins Bloomberg School of Public Health Baltimore Maryland USA; ^47^ Epidemiology and Prevention Group, Center for Public Health Sciences National Cancer Center Tokyo Japan; ^48^ Cancer Prevention Program, Public Health Sciences Division Fred Hutchinson Cancer Research Center Seattle Washington USA; ^49^ Department of Surgical Sciences Uppsala University Uppsala Sweden; ^50^ Hokkaido University Faculty of Medicine Sapporo Japan; ^51^ Department of Medical Biosciences Umeå University Umeå Sweden; ^52^ Department of Radiation Oncology Memorial Sloan Kettering Cancer Center New York New York USA; ^53^ Department of Public Health and Nursing, Faculty of Medicine Norwegian University of Science and Technology Trondheim Norway; ^54^ Director Office, International Agency for Research on Cancer World Health Organization Lyon France; ^55^ Department of Endocrinology and Diabetes Fiona Stanley Hospital Perth Western Australia Australia; ^56^ UK Biobank Ltd Stockport UK

**Keywords:** aggressive prostate cancer, Mendelian randomisation, prostate cancer, SHBG, testosterone

## Abstract

Previous studies had limited power to assess the associations of testosterone with aggressive disease as a primary endpoint. Further, the association of genetically predicted testosterone with aggressive disease is not known. We investigated the associations of calculated free and measured total testosterone and sex hormone‐binding globulin (SHBG) with aggressive, overall and early‐onset prostate cancer. In blood‐based analyses, odds ratios (OR) and 95% confidence intervals (CI) for prostate cancer were estimated using conditional logistic regression from prospective analysis of biomarker concentrations in the Endogenous Hormones, Nutritional Biomarkers and Prostate Cancer Collaborative Group (up to 25 studies, 14 944 cases and 36 752 controls, including 1870 aggressive prostate cancers). In Mendelian randomisation (MR) analyses, using instruments identified using UK Biobank (up to 194 453 men) and outcome data from PRACTICAL (up to 79 148 cases and 61 106 controls, including 15 167 aggressive cancers), ORs were estimated using the inverse‐variance weighted method. Free testosterone was associated with aggressive disease in MR analyses (OR per 1 SD = 1.23, 95% CI = 1.08‐1.40). In blood‐based analyses there was no association with aggressive disease overall, but there was heterogeneity by age at blood collection (OR for men aged <60 years 1.14, CI = 1.02‐1.28; *P*_het_ = .0003: inverse association for older ages). Associations for free testosterone were positive for overall prostate cancer (MR: 1.20, 1.08‐1.34; blood‐based: 1.03, 1.01‐1.05) and early‐onset prostate cancer (MR: 1.37, 1.09‐1.73; blood‐based: 1.08, 0.98‐1.19). SHBG and total testosterone were inversely associated with overall prostate cancer in blood‐based analyses, with null associations in MR analysis. Our results support free testosterone, rather than total testosterone, in the development of prostate cancer, including aggressive subgroups.

## Graphical abstract

What's new?

The Endogenous Hormones, Nutritional Biomarkers and Prostate Cancer Collaborative Group (EHNBPCCG) is a pooled dataset of prospective studies of prostate cancer risk. Using this data, the authors conducted blood‐based analysis and Mendelian randomisation analysis to determine the association between circulating testosterone and overall risk of prostate cancer, as well as looking at risk of aggressive disease and early‐onset cancer separately. They found strong evidence that higher concentrations of circulating testosterone increases the risk of prostate cancer, including aggressive subtypes. This is the largest collection of prospective blood‐based observational and genetic data on sex hormones and prostate cancer risk to date.

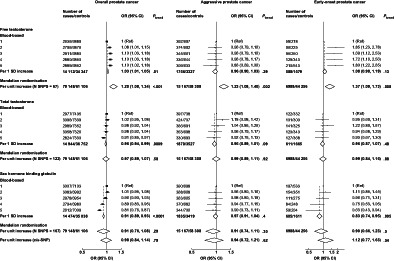

**Abbreviations**
BMI: body mass index
CI: confidence interval
EHNBPCCG: The Endogenous Hormones, Nutritional Biomarkers and Prostate Cancer Collaborative Group
IGF: insulin‐like growth factor
IGFBP: insulin‐like growth factor binding protein
MR: Mendelian randomisation
MR‐PRESSO: MR residual sum and outlier
MR‐RAPS: MR robust adjusted profile score
OR: odds ratio
PRACTICAL: Prostate Cancer Association Group to Investigate Cancer Associated Alterations in the Genome
PSA: prostate‐specific antigen
SHBG: sex hormone binding globulin

## 1 INTRODUCTION

Prostate cancer is the second most common cancer in men worldwide and a leading cause of cancer death.^1^ Blood‐based and genetic epidemiological studies show evidence of an association between circulating concentrations of calculated free testosterone and risk of overall prostate cancer.^2^, ^3^, ^4^, ^5^, ^6^ The association is biologically plausible because androgens are integral to the maintenance of prostate function.^7^ In the circulation, testosterone is bound to sex hormone‐binding globulin (SHBG) and albumin. Approximately 2% of total testosterone circulates unbound or ‘free’, and according to the free hormone hypothesis is more biologically active.^8^ Prostate cancer varies in aggressiveness and tumours also vary by age of onset, and risk factors for these subgroups may be different from those for overall prostate cancer,^9^ but previous studies have lacked statistical power to assess associations of testosterone with prostate cancer subgroups.

The Endogenous Hormones, Nutritional Biomarkers and Prostate Cancer Collaborative Group (EHNBPCCG) is a pooled individual participant case‐control dataset of prospective studies of risk of prostate cancer and associated risk factors. Previous analyses of the associations of circulating testosterone concentrations using the EHNBPCCG dataset were based on up to 6900 cases and 12 100 controls.^2^ We observed that men with very low free testosterone had a lower risk of overall prostate cancer, but we had limited power to investigate the associations with aggressive disease as a primary endpoint. This dataset has since been expanded to include more than double the number of prostate cancer cases, including 1900 aggressive and 600 early‐onset cases.

Mendelian randomisation (MR) analyses, which use genetic instruments to predict average adult exposures, are less likely than blood‐based studies to be affected by confounding factors or reverse causation, and are often considered to be a more reliable method for causal inference.^10^ Therefore, we carried‐out two‐sample MR analyses, using instruments identified from UK Biobank (up to 194 500 men) and genetic data from the PRACTICAL consortium (up to 79 000 prostate cancer cases [15 000 aggressive and 7000 early‐onset subgroups] and 61 000 controls).^11^, ^12^

Using these two international consortia, we aimed to extend our prior study in the EHNBPCCG to assess the associations of circulating concentrations of calculated free testosterone, as well as total testosterone and SHBG which are used to calculate free testosterone, with overall, aggressive and early‐onset prostate cancer risk using blood‐based and genetic methods; using these complementary approaches can provide more robust evidence for causal inference.

## 2 MATERIALS AND METHODS

### 2.1 Endogenous Hormones, Nutritional Biomarkers and Prostate Cancer Collaborative Group

#### 2.1.1 Data collection and study designs

Individual participant data were available from up to 25 prospective studies with total testosterone and SHBG measurements. Participating studies are listed in Supplementary Table 1 and further details of data collection and processing are provided in the Supplementary Material (Appendix S2). Matching criteria are shown in Supplementary Table 2. Assay details and hormone measurement data are listed in Supplementary Table 3.

#### 2.1.2 Data processing and outcomes

Free testosterone concentrations were estimated using a formula based on the law of mass action from measured total testosterone and SHBG concentrations,^13^, ^14^ assuming a constant albumin concentration of 43 g/L.

Disease definitions were as defined by the PRACTICAL consortium.^11^, ^12^ Aggressive prostate cancer was categorised as ‘yes’ for any of the following: disease metastases at diagnosis (M1), Gleason score 8+ (or equivalent), prostate cancer death (defined as death from prostate cancer) or prostate‐specific antigen (PSA) >100 ng/mL. Early‐onset prostate cancer was defined as a diagnosis aged ≤55 years. Further details can be found in the Supplementary Methods (Appendix S2).

#### 2.1.3 Statistical analysis

Conditional logistic regression was used to estimate prostate cancer risk by free and total testosterone and SHBG concentrations. Analyses were conditioned on the study‐specific matching variables and adjusted for age at blood collection, body mass index (BMI), height, smoking status, alcohol consumption, racial/ethnic group, education, married/cohabiting and diabetes status. Biomarkers were standardised by study and entered into the model as continuous variables, so each increment represents a 1 study‐specific SD increase in biomarker concentration. For categorical analyses, biomarkers were categorised into study‐specific fifths with cut‐points determined in controls.^15^ Further details are available in the Supplementary Methods (Appendix S2).

#### 2.1.4 Further analyses

We examined heterogeneity in the associations of each biomarker with prostate cancer by participant characteristics and study (Supplementary Methods, Appendix S2). Subgroups were defined a priori based on the availability of data and previous analyses using this dataset.^2^, ^5^ To further investigate the apparent heterogeneity by age at blood collection, we examined associations of free testosterone with overall and aggressive prostate cancer in fifths, stratified by age at blood collection (<60; 60+ years).

We also investigated associations in models conditioned on the matching variables but not further adjusted, associations in tenths, and estimates per 80th percentile increase. Associations were also examined following mutual adjustment for other biomarkers (including insulin‐like growth factors [IGF‐I, II] and IGF binding proteins [IGFBP‐1,2,3]), and we tested for interactions between these biomarkers. Stratified analyses and associations in tenths were not investigated for early‐onset disease due to the limited number of cases.

### 2.2 Mendelian randomisation analyses

#### 2.2.1 Genetic instruments for hormone concentrations

Summary GWAS results for circulating calculated free and total testosterone and SHBG for men in UK Biobank were extracted from a published analysis (based on up to 194 453 men of white European ancestry)^4^ (Supplementary Methods, Appendix S2). UK Biobank participants were aged 40‐69 years at blood collection (mean age = 56.5 years). We pruned single nucleotide polymorphisms (SNPs) by a linkage disequilibrium threshold of *r* ^2^ < .001.

#### 2.2.2 Genetic associations with prostate cancer

For each of the SNPs included as an instrument for free testosterone, total testosterone and SHBG, we obtained the association with prostate cancer risk from the PRACTICAL consortium (including GAME‐ON/ELLIPSE).^11^, ^12^ Individual studies included in these consortia are available from Schumacher et al.^11^ Associations with overall prostate cancer risk were generated from 79 148 prostate cancer cases and 61 106 controls, aggressive from 15 167 cases and 58 308 controls, and early‐onset disease from 6988 cases and 44 256 controls.^11^, ^12^ Genetic data for UK Biobank participants were not included in this dataset.

#### 2.2.3 Statistical analysis

The MR estimation for hormones was conducted using the inverse‐variance weighted (IVW) method.^16^ We additionally calculated the *I* ^2^ statistic to assess measurement error in SNP‐exposure associations^17^ and Cochran's Q statistic for heterogeneity between the MR estimates for each SNP.^18^ PhenoScanner was used to assess pleiotropy of the genetic instruments.^19^ As sensitivity analyses, we used the MR residual sum and outlier (MR‐PRESSO) and MR robust adjusted profile score (MR‐RAPS) to investigate the role of SNP outliers,^20^ and the weighted median, MR‐Egger and the contamination mixture method to investigate horizontal pleiotropy.^21^, ^22^, ^23^

For SHBG, we additionally investigated associations of the *cis*‐SNP with prostate cancer risk, as this *cis*‐SNP may be less likely to be affected by horizontal pleiotropy than *trans*‐SNPs.^24^ Associations of the *cis*‐SNP with prostate cancer were assessed using the Wald ratio.

Details of statistical software and packages used are available in the Supplementary Methods (Appendix S2). All tests of significance were two‐sided, and *P*‐values <.05 were considered statistically significant.

## 3 RESULTS

### 3.1 Study and participant characteristics in the blood‐based analyses

A total of 25 studies, contributing up to 14 944 cases and 36 752 controls, were included in these analyses. Prostate cancer was classified as aggressive in 1870 cases and early‐onset in 611 cases. Study participants were predominantly of white European ancestry (90%) (Table 1).

**TABLE 1:** Characteristics of prostate cancer cases and controls in the EHNBPCCG participants

		Cases		
	Controls	Overall	Aggressive^a^	Early‐onset^b^
N	36 752	14 944	1870	611
Age at blood collection (yr), mean (SD)	61.0 (8.4)	60.8 (8.6)	62.1 (8.4)	46.7 (5.7)
Height (cm), mean (SD)	174.6 (7.2)	174.7 (7.3)	173.7 (7.8)	177.1 (6.9)
BMI (kg/m^2^), mean (SD)	27.4 (4.1)	26.9 (3.7)	27.1 (4.0)	26.5 (3.7)
PSA at blood collection (ng/mL), med (IQR)	0.9 (1.2)	2.3 (3.2)	3.0 (5.6)	1.6 (2.5)
Time from blood collection to diagnosis, mean (SD)	–	6.5 (5.9)	7.5 (7.1)	5.8 (5.3)
Age at diagnosis, mean (SD)	–	67.3 (6.7)	67.0 (6.2)	52.5 (2.5)
Racial/ethnic group, N (%)				
White	33 645 (91.5)	13 586 (90.9)	1676 (89.6)	559 (91.5)
Black	1222 (3.3)	524 (3.5)	57 (3.0)	31 (5.1)
East Asian	875 (2.4)	484 (3.2)	89 (4.8)	3 (0.5)
Other	678 (1.8)	236 (1.6)	17 (0.9)	10 (1.6)
Not known	332 (0.9)	114 (0.8)	31 (1.7)	8 (1.3)
Smoking status, N (%)				
Never	13 868 (37.7)	5681 (38.0)	599 (32.0)	273 (44.7)
Ex	15 548 (42.3)	6329 (42.4)	815 (43.6)	160 (26.2)
Current	5674 (15.4)	2351 (15.7)	407 (21.8)	140 (22.9)
Not known	1662 (4.5)	583 (3.9)	49 (2.6)	38 (6.2)
Alcohol consumption (g ethanol/day), N (%)				
Nondrinker	2673 (7.3)	1615 (10.8)	250 (13.4)	46 (7.5)
<10	8189 (22.3)	3752 (25.1)	484 (25.9)	140 (22.9)
10+	19 198 (52.2)	7309 (48.9)	883 (47.2)	300 (49.1)
Not known	6692 (18.2)	2268 (15.2)	253 (13.5)	125 (20.5)
Diabetes status, N (%)				
Yes	2887 (7.9)	819 (5.5)	122 (6.5)	13 (2.1)
No	28 745 (78.2)	11 913 (79.7)	1487 (79.5)	467 (76.4)
Not known	5120 (13.9)	2212 (14.8)	261 (14.0)	131 (21.4)
Married/cohabiting, N (%)				
Yes	9767 (26.6)	6790 (45.4)	1295 (69.3)	222 (36.3)
No	1461 (4.0)	958 (6.4)	183 (9.8)	39 (6.4)
Not known	25 524 (69.4)	7196 (48.2)	392 (21.0)	350 (57.3)

*Note*: Some aggressive disease characterisation data were available from 88% of included studies.

Abbreviations: BMI, body mass index; IQR, interquartile range; PSA, prostate‐specific antigen.

^a^ Aggressive disease was defined as Gleason Score 8+, death from prostate cancer, metastatic disease or PSA >100 ng/mL.

^b^ Early‐onset defined as diagnosed aged ≤55 years.

Prostate cancer characteristics by study are displayed in Supplementary Table 4. Mean age at blood collection for each study ranged from 33.8 to 76.8 years (overall mean = 61.0 years, SD = 8.5 years). Cases were diagnosed a mean of 6.5 years (SD = 5.9) after blood collection, and the mean age at diagnosis was 67.3 years (SD = 6.7) (Table 1). Partial correlations between biomarkers ranged from *r* = −0.04 (SHBG and PSA) to *r* = 0.77 (calculated free and total testosterone) (Supplementary Table 5).

### 3.2 Free testosterone

**FIGURE 1:**
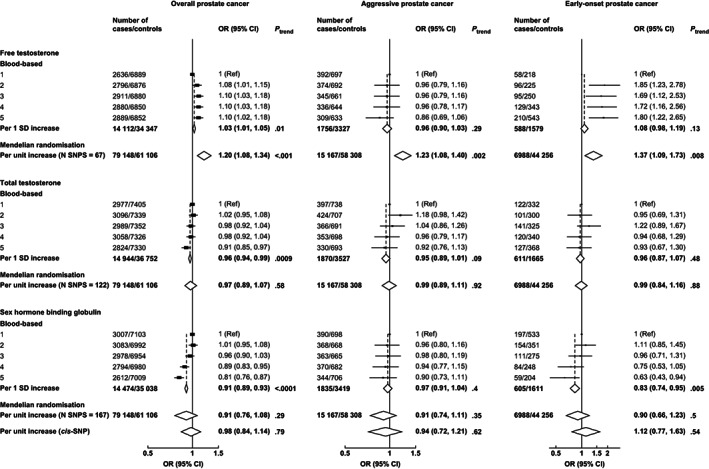
Risks of overall, aggressive* and early‐onset^†^ prostate cancer in by study‐specific fifths of hormone concentrations (blood‐based only) and unit increment (blood‐based and MR). Blood‐based estimates are from logistic regression conditioned on the matching variables and adjusted for age, BMI, height, alcohol intake, smoking status, marital status, education status, racial/ethnic group and diabetes status. The position of each square indicates the magnitude of the odds ratio, and the area of the square is proportional to the inverse of the variance of the logarithm of the OR. The length of the horizontal line through the square indicates the 95% CI. MR risk estimates are estimated using the inverse variance weighted method for the full instrument methods and the Wald ratio in the *cis*‐SNP analyses (where applicable). In MR analyses, biomarker transformations are outlined in the Supplementary Methods (Appendix S2). *Aggressive cancer defined as Gleason grade 8+, or prostate cancer death or metastases or PSA >100 ng/mL. ^†^Early‐onset defined as diagnosed ≤55 years. BMI, body mass index; CI, confidence interval; OR, odds ratio; PSA, prostate‐specific antigen; SNP, single nucleotide polymorphism

The association of calculated free testosterone with overall prostate cancer risk was significant in both blood‐based (OR per 1 SD increment = 1.03, 95% CI 1.01‐1.05) and MR analyses (OR per genetically predicted 1 SD increment = 1.20, 1.08‐1.34) (Figure 1). Higher free testosterone was associated with a higher risk of aggressive prostate cancer in the MR analysis (1.23, 1.08‐1.40), but there was no evidence of an association in the blood‐based analysis (0.96, 0.90‐1.03) (Figure 1). MR sensitivity analyses generally supported the associations of free testosterone with overall and aggressive prostate cancer, except for MR‐Egger, although the MR‐Egger intercepts did not indicate directional pleiotropy (Table 2).

**TABLE 2:** Mendelian randomisation estimates between genetically predicted circulating biomarker concentrations and prostate cancer risk

			Overall prostate cancer (79 148 cases, 61 106 controls)		Aggressive prostate cancer^a^ (15 167 cases, 58 308 controls)		Early‐onset prostate cancer^b^ (6988 cases, 44 256 controls)	
	Variance explained	N SNPs	OR per unit increment in biomarker (95% CI)	*P*	OR per unit increment in biomarker (95% CI)	*P*	OR per unit increment in biomarker (95% CI)	*P*
Free testosterone (SD = 59.5 pmol/L)								
Inverse‐variance weighted	3.8%	67	1.20 (1.08, 1.34)	0.0006	1.23 (1.08, 1.40)	0.002	1.37 (1.09, 1.73)	0.008
Weighted median			1.12 (1.01, 1.25)	0.04	1.19 (0.99, 1.43)	0.07	1.16 (0.89, 1.52)	0.27
MR‐Egger			1.07 (0.87, 1.31)	0.53	1.03 (0.80, 1.32)	0.84	1.09 (0.69, 1.72)	0.71
MR‐Egger intercept				0.20		0.11		0.26
MR‐RAPS			1.16 (1.05, 1.28)	0.002	1.20 (1.05, 1.36)	0.01	1.33 (1.05, 1.67)	0.02
MR‐PRESSO			1.13 (1.05, 1.22)	0.002	1.23 (1.08, 1.40)^c^	0.002	1.33 (1.07, 1.65)	0.01
Contamination mixture			1.12 (1.04, 1.22)	0.007	1.20 (1.00, 1.39)	0.05	1.22 (0.94, 1.97)	0.14
Total testosterone (SD = 3.8 nmol/L)								
Inverse‐variance weighted	7.5%	122	0.97 (0.89, 1.07)	0.58	0.99 (0.89, 1.11)	0.92	0.99 (0.84, 1.16)	0.88
Weighted median			0.99 (0.91, 1.08)	0.89	0.99 (0.86, 1.14)	0.84	1.07 (0.86, 1.32)	0.53
MR‐Egger			0.99 (0.85, 1.15)	0.92	1.06 (0.89, 1.26)	0.53	0.95 (0.73, 1.25)	0.73
MR‐Egger intercept				0.77		0.39		0.76
MR‐RAPS			1.04 (0.94, 1.14)	0.45	1.03 (0.93, 1.14)	0.62	1.00 (0.85, 1.18)	0.99
MR‐PRESSO			1.02 (0.95, 1.09)	0.60	0.92 (0.79, 1.08)	0.30	1.01 (0.88, 1.17)	0.88
Contamination mixture			1.06 (0.99, 1.17)	0.09	1.02 (0.94, 1.14)	0.54	1.05 (0.86, 1.21)	0.72
SHBG (SD = 16.5 nmol/L)								
Inverse‐variance weighted	15.0%	168	0.91 (0.76, 1.08)	0.29	0.91 (0.74, 1.11)	0.35	0.90 (0.66, 1.23)	0.50
Weighted median			0.98 (0.85, 1.13)	0.79	0.94 (0.74, 1.18)	0.58	1.12 (0.79, 1.58)	0.52
MR‐Egger			0.99 (0.76, 1.27)	0.92	1.05 (0.78, 1.40)	0.76	0.97 (0.62, 1.52)	0.89
MR‐Egger intercept				0.38		0.18		0.64
MR‐RAPS			1.01 (0.86, 1.19)	0.87	0.95 (0.79, 1.15)	0.60	0.93 (0.70, 1.25)	0.63
MR‐PRESSO			0.98 (0.88, 1.10)	0.76	0.93 (0.79, 1.09)	0.36	0.98 (0.77, 1.24)	0.86
Contamination mixture			0.96 (0.86, 1.07)	0.55	0.90 (0.77, 1.05)	0.21	1.01 (0.70, 1.30)	0.92
*cis*‐SNP (rs1799941)	4.2%	1	0.98 (0.84, 1.14)	0.79	0.94 (0.72, 1.21)	0.62	1.12 (0.77, 1.63)	0.54

*Note*: Biomarker transformations are outlined in the Supplementary Methods (Appendix S2).

Abbreviations: CI, confidence interval; MR, Mendelian randomisation; OR, odds ratio; PRESSO, pleiotropy residual sum and outlier; RAPS, robust adjusted profile score; SHBG, sex hormone‐binding globulin.

^a^ Aggressive disease was defined as Gleason Score 8+, death from prostate cancer, metastatic disease or PSA >100 ng/mL.

^b^ Early‐onset defined as diagnosed aged ≤55 years.

^c^ No statistically significant outliers detected.

In the MR analysis, predicted free testosterone was associated with an increased risk of early‐onset disease (1.37, 1.09‐1.73), and the relationship was directionally consistent in blood‐based analyses (1.08, 0.98‐1.19) (Figure 1). The associations with early‐onset disease were less robust in the MR sensitivity analyses but were directionally consistent (Table 2).

### 3.3 Total testosterone

The OR for total testosterone in relation to overall prostate cancer was 0.96 (0.94‐0.99) in blood‐based analysis and 0.97 (0.89‐1.07) in MR analysis (Figure 1 and Table 2). Total testosterone was not associated with aggressive or early‐onset disease in blood‐based or in the MR analyses (Figure 1 and Table 2).

### 3.4 Sex hormone‐binding globulin

SHBG was inversely associated with overall and early‐onset prostate cancer in blood‐based analyses (0.91, 0.89‐0.93 and 0.83, 0.74‐0.95, respectively), but was not associated with aggressive disease risk (0.97, 0.91‐1.04) (Figure 1). In the MR analyses, SHBG was not associated with prostate cancer risk using the full instrument or the *cis*‐SNP instrument (Figure 1 and Table 2).

#### 3.4.1 Further analyses – blood‐based analysis

**FIGURE 2:**
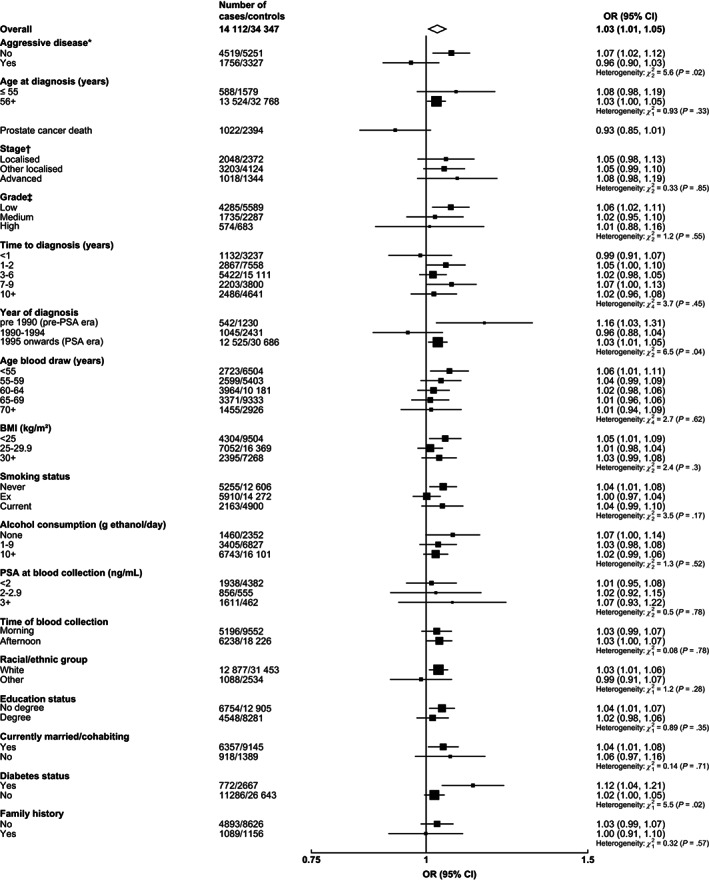
Odds ratio (95% CIs) for overall prostate cancer per study‐specific 1 SD increment of free testosterone concentration by subgroup. Estimates are from logistic regression conditioned on the matching variables and adjusted for age, BMI, height, alcohol intake, smoking status, marital status, education status, racial/ethnic group and diabetes status. The position of each square indicates the magnitude of the OR, and the area of the square is proportional to the inverse of the variance of the logarithm of the OR). The length of the horizontal line through the square indicates the 95% CI. Tests for heterogeneity for case‐defined factors were obtained by fitting separate models for each subgroup and assuming independence of the ORs using a method analogous to a metaanalysis. Tests for heterogeneity for non‐case‐defined factors were assessed with a *χ* ^2^ test of interaction between subgroup and the binary variable. *Aggressive cancer defined as Gleason grade 8+, or prostate cancer death or metastases or PSA >100 ng/mL. ^†^Localised defined as TNM stage <T2 with no reported lymph node involvement or metastases or stage I; other localised stage if TNM stage T2 with no reported lymph node involvement or metastases, stage II or equivalent; advanced stage if they were TNM stage T3 or T4 and/or N1+ and/or M1, stage III‐IV or equivalent. ^‡^Low grade defined as Gleason score was <7 or equivalent (ie, extent of differentiation good, moderate); medium grade if Gleason score was 7 (ie, poorly differentiated); high grade if the Gleason score was ≥8 or equivalent (ie, undifferentiated). BMI, body mass index; CI, confidence interval; OR, odds ratio; PSA, prostate‐specific antigen

There was significant heterogeneity in the associations of free testosterone with risk according to the prostate cancer aggressiveness; higher free testosterone concentration was associated with an increased risk of nonaggressive (1.07, 1.02‐1.12), but not aggressive disease (0.96, 0.90‐1.03; *P*_het_ = .02) (Figure 2). Men with diabetes also had a larger magnitude of association of free testosterone with overall prostate cancer than men without diabetes (1.12, 1.04‐1.21 and 1.02, 1.00‐1.05, respectively; *P*_het_ = .02) (Figure 2).

**FIGURE 3:**
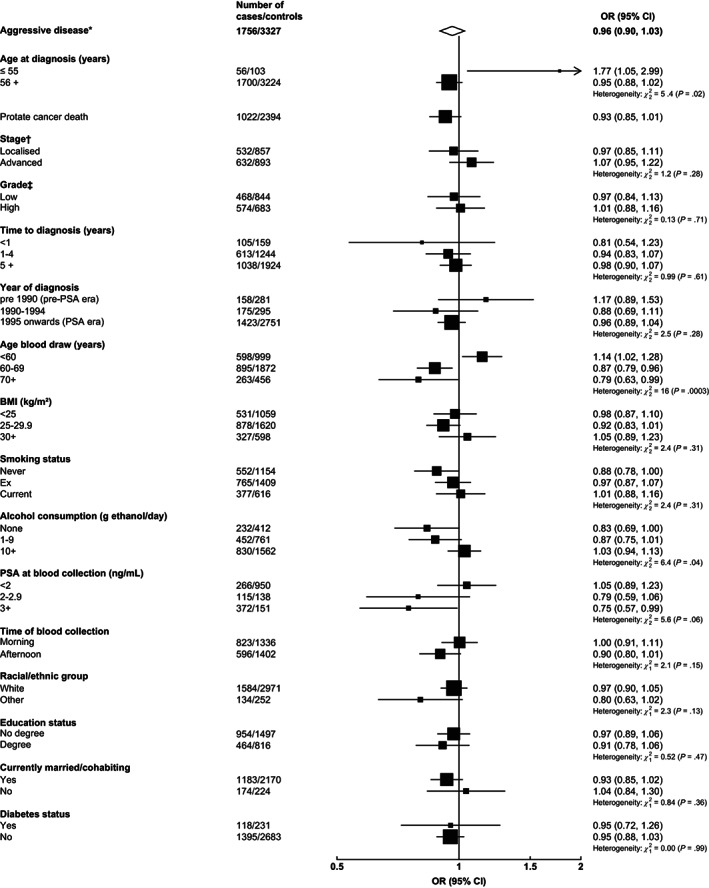
Odds ratio (95% CIs) for aggressive* prostate cancer per study‐specific 1 SD increment of free testosterone concentration by subgroup. Estimates are from logistic regression conditioned on the matching variables and adjusted for age, BMI, height, alcohol intake, smoking status, marital status, education status, racial/ethnic group and diabetes status. The position of each square indicates the magnitude of the OR, and the area of the square is proportional to theinverse of the variance of the logarithm of the OR). The length of the horizontal line through the square indicates the 95% CI. Tests for heterogeneity for case‐defined factors were obtained by fitting separate models for each subgroup and assuming independence of the ORs using a method analogous to a metaanalysis. Tests for heterogeneity for non‐case‐defined factors were assessed with a *χ* ^2^ test of interaction between subgroup and the binary variable. *Aggressive cancer defined as Gleason grade 8+, or prostate cancer death, or metastases or PSA >100 ng/mL. ^†^Localised defined as TNM stage <T2 with no reported lymph node involvement or metastases or stage I, or TNM stage T2 with no reported lymph node involvement or metastases, stage II, or equivalent; advanced stage if they were TNM stage T3 or T4 and/or N1+ and/or M1, stage III‐IV or equivalent. ^‡^Low grade defined as Gleason score was <8 or equivalent (ie, extent of differentiation good, moderate, poor); high grade if the Gleason score was ≥8 or equivalent (ie, undifferentiated). BMI, body mass index; CI, confidence interval; OR, odds ratio; PSA, prostate‐specific antigen

For aggressive disease risk, there was significant heterogeneity in the associations by age at blood collection; free testosterone was positively associated with aggressive prostate cancer (1.14, 1.02‐1.28) for men whose blood was collected at ages <60 years, but the relationship was inverse for men whose blood was collected at older ages (0.87, 0.79‐0.96 and 0.79, 0.63‐0.99 for men whose blood was collected aged 60‐69 and 70+ years, respectively) (*P*_het_ = .0003) (Figure 3). In analyses based on fifths of free testosterone, there was a positive dose‐response relationship of free testosterone with overall and aggressive prostate cancer for men whose blood was collected at <60 years, while for men whose blood was collected at an older age, the relationship was null with overall prostate cancer and inverse with aggressive prostate cancer (Supplementary Figure 1). Higher free testosterone was also associated with an elevated risk of early‐onset aggressive disease (1.77, 1.05‐2.99) but was not associated with aggressive disease for men diagnosed later in life (0.95, 0.88‐1.02; *P*_het_ = .02) (Figure 3), although there was a small number of cases of early‐onset aggressive disease (n = 56).

The associations of total testosterone and SHBG with overall and aggressive prostate cancer were generally consistent by subgroups (Supplementary Figures 2‐5). Total testosterone was inversely associated with prostate cancer death (0.90, 0.82‐0.97) and positively associated with early‐onset aggressive prostate cancer (2.40, 1.28‐4.52), while for men diagnosed with aggressive disease aged >55 years the OR was 0.94 (0.88‐1.00; *P*_het_ = .0004) (Supplementary Figure 3).

There was no statistically significant heterogeneity in the associations with overall and aggressive prostate cancer by study (Supplementary Figures 6‐11), except for free testosterone and aggressive prostate cancer (*P*_het_ = .02) (Supplementary Figure 7). Associations were broadly similar in unadjusted matched analyses (Supplementary Figure 12), study‐specific tenths (Supplementary Figure 13), per 80%tile increase (Supplementary Table 6) and following mutual adjustment for other biomarkers (Supplementary Table 7).

There were significant interactions in the associations of total testosterone with overall and aggressive prostate cancer by SHBG concentrations (Supplementary Tables 8 and 9). SHBG was positively associated with aggressive disease risk for men with lower IGFBP‐1 concentrations (1.23, 1.00‐1.51), and the relationship was inverse for men with higher IGFBP‐1 concentrations (0.85, 0.71‐1.02; *P*_het_ = .01) (Supplementary Table 9).

#### 3.4.2 Further analyses – Mendelian randomisation

There was no strong evidence of measurement error in the genetic instruments for the biomarkers (*I* ^2^ > 0.96). There was significant heterogeneity in the MR estimates for the SNPs with overall disease, and for aggressive and early‐onset disease (Cochran's Q *P* < .001), except for the association of free testosterone with aggressive disease (*P* = .12). Using PhenoScanner, 175, 355 and 358 traits were linked to SNPs for free testosterone, SHBG and total testosterone concentrations, respectively, particularly adiposity and height, and SNPs associated with free testosterone were frequently related to age at puberty (*P* < 5 × 10^−8^) (Supplementary Figures 14‐16). Traits linked to the SHBG *cis*‐SNP (rs1799941) are shown in Supplementary Table 10. MR scatterplots and tables are found in Supplementary Figures 17‐19 and Supplementary Tables 11‐13.

## 4 DISCUSSION

In this first comprehensive analysis with both blood‐based and genetic data, our results suggest that higher calculated free testosterone is associated with an elevated risk for prostate cancer, including aggressive disease. Neither circulating total testosterone nor SHBG was associated with elevated risks for prostate cancer.

The strong genetic evidence in our MR analyses (which are less likely to be affected by biases such as confounding, reverse causation and detection bias) for a role of free testosterone, alongside the well‐characterised lower risk of prostate cancer in men diagnosed with Klinefelter's syndrome^25^ (a genetic abnormality which is characterised by life‐long clinically low total and free testosterone concentrations^26^), indicates a probable causal relationship of free testosterone with prostate cancer, including with aggressive disease. While in our blood‐based analyses the overall association of free testosterone with aggressive prostate cancer was null, there was evidence of a positive association with aggressive disease for men whose blood who was collected at a younger age. However, we observed inverse associations of free testosterone with aggressive disease for men whose blood was collected at an older age, which warrants further consideration. Differences between the associations of genetically predicted free testosterone and measured blood concentrations with prostate cancer risk may implicate the importance of free testosterone concentrations in younger adulthood. Free testosterone concentrations decline with older age, partly due to cumulative environmental influences, therefore free testosterone concentrations in middle and older age may not be representative of life‐long exposure to free testosterone concentrations, which will attenuate risk estimates.^27^, ^28^, ^29^, ^30^, ^31^, ^32^ There was also some evidence of heterogeneity in the blood‐based association of free testosterone with aggressive disease by study, which may relate to differences in participant and tumour characteristics.

As well as the blood‐based and genetic evidence that we describe here, two randomised controlled trials using 5α‐reductase inhibitors, which aimed to reduce intraprostatic androgen signalling by reducing dihydrotestosterone concentrations by 80‐90%,^33^ have reported 23‐25% lower risks of overall prostate cancer. However, these trials also reported 27‐58% increased risks of high‐grade tumours,^34^, ^35^ possibly due to changes in prostate morphology, function biasing tumour diagnostic grading, and/or the early development of partial androgen insensitivity in more aggressive tumours (in comparison with low‐grade tumours)^36^, ^37^; long‐term follow‐up of these trials does not support an effect on risk of prostate cancer mortality.^38^

For total testosterone and SHBG the MR results were null suggesting no direct effect, whereas the blood‐based analyses were inverse for both; it is possible that the inverse results for testosterone are due to reverse causation, but results did not suggest this for SHBG and the explanation for the blood‐based results remains unclear.

These analyses have several strengths. This is the largest collection of prospective blood‐based and genetic data on sex hormones and prostate cancer risk available, representing almost all the available data worldwide. This large sample size maximised power to assess associations robustly and enabled us to investigate associations across subgroups. Further, by incorporating blood‐based and MR methods we were able to use different lines of evidence to inform causal inference.^39^

Limitations include that we used calculated rather than directly measured free testosterone concentrations using equilibrium dialysis,^40^ we have used a validated formula to estimate concentrations and these are well correlated.^41^, ^42^ It has also been suggested that the bioavailable fraction of testosterone is the sum of free and albumin‐bound testosterone rather than solely the free fraction,^43^ but it is not possible in our data to distinguish between these hypotheses because estimates of these fractions from the formula are perfectly correlated. Furthermore, the predictive value of peripheral free testosterone as an indicator of intraprostatic signalling remains under debate.^44^ Our analyses relied on single biomarker measurements, and although these biomarkers have good reproducibility over a 4‐to‐5‐year period (intraclass correlation coefficients 0.54‐0.82),^2^ longitudinal studies have shown that free testosterone declines continually throughout adulthood^45^; this may lead to underestimates of risk.^46^ Participants in the EHNBPCCG dataset were predominantly white and therefore we were underpowered to investigate associations for other racial/ethnic groups. Prospective epidemiological studies were generally based on older men, therefore we had more limited power to investigate associations in younger participants.

In the MR analyses of free testosterone, we observed weaker relationships using MR‐Egger. MR‐Egger is less susceptible to confounding from possibly pleiotropic variants that have stronger effects on the outcome than the exposure. However, this approach is also subject to reduced power and therefore does not necessarily imply the absence of a causal effect in the context of consistent sensitivity analyses and balanced pleiotropy.^21^, ^47^ Further, testosterone is a steroid and therefore no *cis‐*genetic instruments are available. These limitations mean that we cannot exclude the possibility that the MR results for free testosterone may be influenced by some horizontal pleiotropy. It is also not known whether the performance of the genetic predictors of free testosterone change with age.^48^ Future genetic and blood‐based research including younger men with repeat measurements and linkage to detailed medical records will help to clarify associations.

The blood‐based results we report here are an extension of our previous paper,^2^ and includes more than double the number of cases, with the incorporation studies including UK Biobank and extended follow‐up from some other studies. Our blood‐based results indicated possible nonlinear relationships with overall prostate cancer (as reported previously)^2^ and with early‐onset prostate cancer, with lower risks of overall and early‐onset prostate cancer for men with low free testosterone concentrations. For MR analyses, genetic instruments were based on summary GWAS results, and we were therefore unable to investigate possible nonlinear associations. For overall prostate cancer we also previously reported a possible increased risk of high‐grade disease; however, we have limited additional data for grade, and therefore we do not include an updated detailed grade analysis as reported in the previous paper.

In conclusion, the findings from these blood‐based and genetic analyses implicate free testosterone in the development of prostate cancer, including aggressive and early‐onset disease.

## AUTHOR CONTRIBUTIONS

Eleanor L. Watts wrote the original manuscript draft, analysed the data, created the visualisations, acquired funding, and led the conceptualisation of the analysis. Georgina K. Fensom, Urwah Noor and Colm D. Andrews contributed to the data curation, pooling and administration. Karl Smith‐Byrne, Marc J. Gunter, Michael V. Holmes, Richard M. Martin and Konstantinos K. Tsilidis contributed to the conceptualisation of the analysis, methodology and review and drafting of the manuscript. Demetrius Albanes, Aurelio Barricarte, H. Bas Bueno‐de‐Mesquita, Chu Chen, Barbara A. Cohn, Niki L. Dimou, Luigi Ferrucci, Leon Flicker, Neal D. Freedman, Graham G. Giles, Edward L. Giovannucci, Gary E. Goodman, Christopher A. Haiman, Graeme J. Hankey, Jiaqi Huang, Wen‐Yi Huang, Lauren M. Hurwitz, Rudolf Kaaks, Paul Knekt, Tatsuhiko Kubo, Hilde Langseth, Gail Laughlin, Loic Le Marchand, Tapio Luostarinen, Robert J. MacInnis, Hanna O. Mäenpää, Satu Männistö, E. Jeffrey Metter, Kazuya Mikami, Lorelei A. Mucci, Anja W. Olsen, Kotaro Ozasa, Domenico Palli, Kathryn L. Penney, Elizabeth A. Platz, Harri Rissanen, Norie Sawada, Jeannette M. Schenk, Pär Stattin, Akiko Tamakoshi, Elin Thysell, Chiaojung Jillian Tsai, Shoichiro Tsugane, Lars Vatten, Elisabete Weiderpass, Stephanie J. Weinstein, Lynne R. Wilkens, Bu B. Yeap, The PRACTICAL consortium, CRUK, BPC3, CAPS, PEGASUS contributed data resources and reviewed and edited the manuscript. Naomi E. Allen contributed data resources and administration and reviewed and edited the manuscript. Aurora Perez‐Cornago, Ruth C. Travis, Timothy J. Key supervised the project, acquired funding, led the conceptualisation of the analysis, and reviewed and edited the manuscript. The work reported in the paper has been performed by the authors, unless clearly specified in the text.

## FUNDING INFORMATION

Centralised pooling, checking and data analysis were supported by Cancer Research UK (grant numbers C8221/A19170 and C8221/A29017). Eleanor L. Watts is supported by the Nuffield Department of Population Health Early Career Research Fellowship. Aurora Perez‐Cornago is supported by a Cancer Research UK Population Research Fellowship (C60192/A28516) and by the World Cancer Research Fund (WCRF UK), as part of the World Cancer Research Fund International grant programme (2019/1953). ATBC was supported in part by the Intramural Research Program of the National Institutes of Health and the National Cancer Institute. CARET is funded by the National Cancer Institute, National Institutes of Health through grants U01‐CA063673, UM1‐CA167462, R01‐CA 78812 and U01‐CA167462. CLUE is funded by the American Institute for Cancer Research, NIH Grants IU01AG18033 and IU01CA86308. HIMS was supported by research grants from the National Health and Medical Research Council of Australia. MEC is supported by the US National Institutes of Health grant U01 CA164973. JACC study was supported by Grants‐in‐Aid for Scientific Research from the Ministry of Education, Culture, Sports, Science and Technology (MEXT) of Japan. RMM was supported by the NIHR Biomedical Research Centre at University Hospitals Bristol and Weston NHS Foundation Trust and the University of Bristol. RMM was supported by a Cancer Research UK (C18281/A29019) programme grant (the Integrative Cancer Epidemiology Programme). The views expressed are those of the author(s) and not necessarily those of the NIHR or the Department of Health and Social Care.

## CONFLICT OF INTEREST

Dr Michael V. Holmes declares unpaid consultancy for Boehringer Ingelheim. The other authors have no conflicts to disclose.

## ETHICS STATEMENT

This analysis reanalysed data and therefore new ethical approval was not required. Each individual study obtained ethical approval and informed consent from participants.

## Supporting information


**Appendix S1** PIs from the PRACTICAL (http://practical.icr.ac.uk/), CRUK, BPC3, CAPS, PEGASUS consortia

**Appendix S2** Supporting Information

## Data Availability

For prospective analysis, the authors do not own the rights for the data contained in the EHNBPCCG dataset and therefore cannot redistribute the data. However, researchers can contact individual studies for access requests. UK Biobank individual‐level data are available upon request, while summary genetic data are publicly available (www.ukbiobank.ac.uk). PRACTICAL genetic data for overall prostate cancer are publicly available, while genetic subgroup data are available upon request (http://practical.icr.ac.uk/). Further details and other data that support the findings of our study are available from the corresponding author upon request.
