# Supplementary material for: Circulating free testosterone and risk of aggressive prostate cancer: Prospective and Mendelian randomisation analyses in international consortia
Source: Int J Cancer. 2022 Jun 7;151(7):1033–46. doi: 10.1002/ijc.34116 (PMC7613289; doi:10.1002/ijc.34116)
Supplement: Supplementary file 2 — Appendix S2 Supporting Information [file IJC-151-1033-s001.pdf]

Circulating free testosterone and risk of aggressive prostate cancer: prospective and Mendelian randomization analyses in international consortia- Supplementary Tables and Figures

Authors

Eleanor L. Watts, Aurora Perez-Cornago, Georgina K. Fensom, Karl Smith-Byrne, Urwah Noor, Colm D. Andrews, Marc J. Gunter, Michael V. Holmes, Richard M. Martin, Konstantinos K. Tsilidis, Demetrius Albanes, Aurelio Barricarte, H. Bas Bueno-de-Mesquita, Chu Chen, Barbara A. Cohn, Niki L. Dimou, Luigi Ferrucci, Leon Flicker, Neal D. Freedman, Graham G. Giles, Edward L. Giovannucci, Gary E. Goodman, Christopher A. Haiman, Graeme J. Hankey, Jiaqi Huang, Wen-Yi Huang, Lauren M. Hurwitz, Rudolf Kaaks, Paul Knekt, Tatsuhiko Kubo, Hilde Langseth, Gail Laughlin, Loic Le Marchand, Tapio Luostarinen, Robert J. MacInnis, Hanna O. Mäenpää, Satu Männistö, E. Jeffrey Metter, Kazuya Mikami, Lorelei A. Mucci, Anja W. Olsen, Kotaro Ozasa, Domenico Palli, Kathryn L. Penney, Elizabeth A. Platz, Harri Rissanen, Norie Sawada, Jeannette M. Schenk, Pär Stattin, Akiko Tamakoshi, Elin Thysell, Chiaojung Jillian Tsai, Shoichiro Tsugane, Lars Vatten, Elisabete Weiderpass, Stephanie J. Weinstein, Lynne R. Wilkens, Bu B. Yeap, The PRACTICAL consortium, CRUK, BPC3, CAPS, PEGASUS, Naomi E. Allen, Timothy J. Key, Ruth C. Travis.

**Table of contents**	**Page**
**Supplementary Methods**	3-6
**Supplementary Tables**	
Supplementary Table 1: Sample populations, recruitment and case ascertainment methods	7-8
Supplementary Table 2: Criteria used by individual studies to match case patients and control subjects	9-10
Supplementary Table 3: Assay methods and geometric mean hormone concentrations by study	11
Supplementary Table 4: Prostate cancer characteristics in participating EHNBPCCG studies	12
Supplementary Table 5: Partial correlation coefficients of circulating biomarkers in prostate cancer controls	13
Supplementary Table 6: Risks of overall, aggressive and early-onset prostate cancer per 80% tile increment in biomarker concentrations in the EHNBPCCG studies	14
Supplementary Table 7: Risks of overall, aggressive and early-onset prostate cancer per study-specific 1 SD increment in biomarker concentrations with mutual adjustment, among cases and their matched controls in EHNBPCCG studies	15
Supplementary Table 8: Risks of overall prostate cancer in relation to biomarkers, stratified by other study-specific median biomarker concentrations in EHNBPCCG studies	16
Supplementary Table 9: Risks of aggressive prostate cancer in relation to biomarkers, stratified by other study-specific median biomarker concentrations in EHNBPCCG studies	17
Supplementary Table 10: Trait associations with the SHBG cis-SNP*	18
Supplementary Table 11: Association of free testosterone SNPs used in 2-sample Mendelian randomization analyses with prostate cancer	19-20
Supplementary Table 12: Association of total testosterone SNPs used in 2-sample Mendelian randomization analyses with prostate cancer	21-23
Supplementary Table 13: Association of SHBG SNPs used in 2-sample Mendelian randomization analyses with prostate cancer	24-27
**Supplementary Figures**	
Supplementary Figure 1: Risks of overall and aggressive* prostate cancer in by study-specific fifths of free testosterone concentrations and per 1 SD increment, stratified by age at blood collection	28

Supplementary Figure 2: Odds ratio (95% CIs) for overall prostate cancer per study-specific 1 SD increment of total testosterone concentration by subgroup 29
Supplementary Figure 3: Odds ratio (95% CIs) for aggressive* prostate cancer per study-specific 1 SD increment of total testosterone concentration by subgroup 30
Supplementary Figure 4: Odds ratio (95% CIs) for overall prostate cancer per study-specific 1 SD increment of SHBG concentration by subgroup 31
Supplementary Figure 5: Odds ratio (95% CIs) for aggressive* prostate cancer per study-specific 1 SD increment of SHBG concentration by subgroup 32
Supplementary Figure 6: Odds ratios (95% confidence intervals) for overall prostate cancer associated with a 1 SD increment in free testosterone by study 33
Supplementary Figure 7: Odds ratios (95% confidence intervals) for aggressive prostate cancer associated with a 1 SD increment in free testosterone by study 34
Supplementary Figure 8: Odds ratios (95% confidence intervals) for prostate cancer associated with a 1 SD increment in total testosterone by study 35
Supplementary Figure 9: Odds ratios (95% confidence intervals) for aggressive prostate cancer associated with a 1 SD increment in total testosterone by study 36
Supplementary Figure 10: Odds ratios (95% confidence intervals) for prostate cancer associated with a 1 SD increment in sex hormone-binding globulin by study 37
Supplementary Figure 11: Odds ratios (95% confidence intervals) for aggressive prostate cancer associated with a 1 SD increment in sex hormone-binding globulin by study 38
Supplementary Figure 12: Risks of overall, aggressive and early-onset prostate cancer* by study-specific fifths of biomarker concentrations and 1 SD increment in the unadjusted model. 39
Supplementary Figure 13: Risks of overall and aggressive* prostate cancer by study-specific tenths of biomarker concentrations and 90%tile increment. 40
Supplementary Figure 14: Traits associated with each free testosterone associated SNP used in the Mendelian randomization analyses 41
Supplementary Figure 15: Traits associated with each total testosterone associated SNP used in the Mendelian randomization analyses 42
Supplementary Figure 16: Traits associated with each SHBG associated SNP used in the Mendelian randomization analyses 43
Supplementary Figure 17: Scatterplot of genetic associations with free testosterone against genetic associations with: A) Overall prostate cancer B) Aggressive prostate cancer C) Early-onset prostate cancer 44
Supplementary Figure 18: Scatterplot of genetic associations with total testosterone against genetic associations with: A) Overall prostate cancer B) Aggressive prostate cancer C) Early-onset prostate cancer 45
Supplementary Figure 19: Scatterplot of genetic associations with SHBG against genetic associations with: A) Overall prostate cancer B) Aggressive prostate cancer C) Early-onset prostate cancer 46

References 47-49

Supplementary methods

i. Endogenous Hormones, Nutritional Biomarkers and Prostate Cancer Collaborative Group

Data collection

Principal investigators were invited to join this collaborative group if they had published or unpublished studies on prostate cancer risk and endogenous sex hormone and/or nutritional biomarker concentrations that had been determined from blood samples collected before diagnosis. Studies were identified by literature searches of computerised bibliographic systems, including PubMed, Web of Science, Cochrane Library, and CancerLit, and through discussions with colleagues[3].

Individual participant data were available from 25 prospective studies by dataset closure on 1st December 2019. We included all prospective studies with total testosterone and sex hormone-binding globulin (SHBG) measurements. In total, 14,944 cases and 36,752 controls, including 1,870 aggressive, and 611 early-onset prostate cancer cases were analysed from the following studies: Alpha-Tocopherol, Beta-Carotene Cancer Prevention Study (ATBC)[4], Baltimore Longitudinal Study of Aging (BLSA)[5, 6], Carotene and Retinol Efficacy Trial (CARET)[7], Child Health and Development Studies (CHDS)[8, 9], CLUE I[10], European Prospective Investigation into Cancer and Nutrition (EPIC)[11], EPIC Norfolk[12], Finnish Mobile Clinic (FMC)[13], Helsinki Heart Study Nordic Biological Specimen Biobank Working Group (HHS NBSBWG)[14, 15], Health In Men Study (HIMS)[16-18], Health Professionals Follow-up Study (HPFS)[19], Japan Collaborative Cohort Study (JACC)[20], Japan–Hawaii Cancer Study (JHCS)[21, 22], Japan Public Health Center-based prospective study (JPHC)[23], Janus[24], Janus NBSBWG[14], Melbourne Collaborative Cohort Study (MCCS)[25], Multiethnic Cohort (MEC)[26], Massachusetts Male Aging Study (MMAS)[27], Northern Sweden Health and Disease Cohort (NSHDC)[14], Prostate Cancer Prevention Trial (PCPT)[28], Physicians' Health Study (PHS)[29], Prostate, Lung, Colorectal and Ovarian Cancer Screening Trial (PLCO)[30, 31], Rancho Bernardo Study (RBS)[32], and UK Biobank[33-35].

The characteristics of these studies in the collaborative analyses are found in their original publications and are summarised in Supplementary Table 1. Most of the studies are case-control studies nested within traditional prospective cohort studies, with some variation in the case mix of these studies according to the prevalence of prostate-specific antigen (PSA) testing within that population during follow-up. For example, there is a generally higher proportion of early stage and low-grade cases in studies from the USA, where there have been relatively high levels of PSA-testing since the mid-1990s, than in studies in European populations where PSA-testing has only more recently started to become common. Two studies (PCPT and PLCO) are observational investigations using data from trials that included organised screening for prostate cancer. In these trials, men with a raised PSA or abnormal digital rectal examination at recruitment-screening were excluded, and the eligible cases were diagnosed during subsequent follow-up (for PCPT the majority being diagnosed at 4 and 7 years after recruitment), with most cases being detected either through PSA-screening (PLCO) or by routine end of study biopsy (PCPT).

Of the randomised trials, data were available for participants in both the intervention and placebo arm in ATBC, CARET, HHS, and PHS. Assay data were only available in the screening arm of the PLCO trial, and the placebo arm in PCPT (as the intervention arm was designed to alter prostate hormone concentrations)[28, 36]. Five studies were cohort or case-cohort analyses (BLSA, HIMS, MCCS, MMAS and UK Biobank), in which hormone concentrations had been measured from stored serum from all, or a subset, of the cohort. To apply a consistent statistical approach across all studies, the cases from the case-cohort studies were matched to up to four participants who were free of prostate cancer at the age at diagnosis of the case, based on our minimal matching criteria (Supplementary Table 2). Some studies used density sample, meaning that an individual could appear more than once in a data file.

Principal investigators were asked to provide data on prostate cancer case or noncase status, and if applicable, a matched-set identifier, as well as participant and tumour characteristics, and prostate cancer mortality. Individual participant data were also contributed for participant characteristics including age, height, weight, smoking status, alcohol consumption, marital status, education achievement, racial/ethnic group, diabetes status, and PSA and other biomarker concentrations at blood collection (where available). Endogenous hormone concentrations requested included testosterone, sex hormone-binding globulin (SHBG), insulin-like growth factor-I (IGF-I), IGF-II, insulin-like growth factor binding proteins (IGFBP-1,-2,-3) and a number of other selected hormones and nutritional biomarkers. Collaborators also provided information on assay and time and date of blood collection. Information was requested about prostate cancer included date of diagnosis and stage and grade of disease, as well as prostate cancer mortality. Men were excluded from the analyses if data were missing for date of birth, blood collection, or diagnosis (for cases) or if they were known to be receiving androgen therapy at blood collection.

Data Processing

Aggressive prostate cancer was categorised as "no" for non-metastatic and Gleason grade <8, and "yes" for any of the following: disease metastases (M1), Gleason score 8+ (stage and grade as recorded at diagnosis), prostate cancer death (defined as death from prostate cancer), or prostate-specific antigen (PSA) >100 ng/mL. In blood-based analyses, non-differentiated tumours were additionally classified as aggressive when Gleason score were not available, and PSA >100 ng/mL either at diagnosis or at blood collection. Prostate cancer was defined as low grade if the Gleason score was <7 or equivalent (i.e. extent of differentiation good, moderate), medium grade if Gleason score was 7 (i.e. poorly differentiated), and high grade if the Gleason score was ≥8 or equivalent (i.e. undifferentiated), or grade "unknown" otherwise.

Cases were defined as being early stage if they were tumor–node–metastasis (TNM) stage <T2 with no reported lymph node involvement or metastases or stage I; other localized stage if they were TNM stage T2 with no reported lymph node involvement or metastases, stage II, or equivalent (i.e. a tumor that does not extend beyond the prostate capsule); advanced stage if they were TNM stage T3 or T4 and/or N1+ and/or M1, stage III–IV, or equivalent (i.e. a tumor extending beyond the prostate capsule and/or lymph node involvement and/or distant metastases); or stage unknown. Early-onset prostate cancer was defined as aged ≤55 years at diagnosis. Disease subgroup definitions were the same for blood-based and genetic analyses.

Statistical analyses

Conditional logistic regression was used to calculate the odds of prostate cancer diagnosis by hormone concentration. Analyses were conditioned on the matching variables and adjusted for age at blood collection (continuous), BMI (<25, 25-27.4, 27.5-29.9, 30+ kg/m^2, unknown (3.0%)), height (<171, 171-175, 176-180, 180+ cm, unknown (3.0%)), smoking status (never, current, previous, unknown (4.3%)), usual alcohol consumption (none, 1-9, 10-19, 20-39, 40+ g/day, unknown (17.3%)), racial/ethnic group (white, black, East Asian, other, unknown (0.8%)), education status (<secondary/high school, secondary/high school, university+, unknown (24.3%)), married/cohabiting (yes, no, unknown (62.2%)), diabetes status (yes, no, unknown (14.5%)).

To account for any systematic differences between the studies in assay methods and blood sample types, biomarkers were standardised by study and entered into the model as a continuous variable. In categorical analyses, biomarkers were categorised into study-specific fifths with cut-points determined in the controls[37].

Further analyses

Tests for heterogeneity for case-defined factors were obtained by fitting separate models for each subgroup and assuming independence of the ORs using a method analogous to a meta-analysis. Tests for heterogeneity for non-case defined factors were assessed with a χ^2-test of interaction between subgroup and the continuous exposure variable. For associations with overall prostate cancer diagnosis, subgroup categories were defined as follows: aggressive disease (yes, no), age at diagnosis (≤55, 56+ years), prostate cancer death (yes), stage (localized, other localized, advanced), grade (low, medium, high), time to diagnosis (<1, 1-2, 3-6, 7-9, 10+ years), year of diagnosis (pre 1990, 1990-1994, 1995 onwards), age at blood draw (≤55, 55-59, 60-64, 65-69, 70+ years), BMI (<25, 25-29.9, 30+ kg/m^2), smoking status (never, ex, current), alcohol consumption (none, 1-9, 10+ g ethanol/day), PSA at blood collection (<2, 2-2.9, 3+ ng/mL), time of blood collection (morning, afternoon), racial/ethnic group (white, other), education status (no degree, degree), currently married/cohabiting (yes, no), diabetes status (yes, no).

For aggressive prostate cancer diagnosis, subgroups were: age at diagnosis (≤55, 56+ years), prostate cancer death (yes), stage (localized/other localized, advanced), grade (low/medium, high), time to diagnosis (<1, 1-4, 5+ years), year of diagnosis (pre 1990, 1990-1994, 1995 onwards), age at blood draw (<60, 60-69, 70+ years), BMI (<25, 25-29.9, 30+ kg/m^2), smoking status (never, ex, current), alcohol consumption (none, 1-9; 10+ g ethanol/day), PSA at blood collection (<2, 2-2.9, 3+ ng/mL), time of blood collection (morning, afternoon), racial/ethnic group (white, other), education status (no degree, degree), currently married/cohabiting (yes, no), diabetes status (yes, no), overnight fast (no, yes). Subgroups were defined *a priori* based on the availability of data and previous analyses using this dataset[38, 39]. To further investigate the apparent heterogeneity by age at blood collection, we examined the associations of free testosterone with overall and aggressive prostate cancer in fifths, stratified by age at blood collection (<60; 60+ years).

For each biomarker, heterogeneity in linear trends of the biomarkers and aggressive and overall prostate cancer between studies was assessed by comparing the χ^2 values for models with and without a (study) × (linear trend) interaction term.

This was tested across between all studies as well as in studies which included organised screening in their study design (PCPT and PLCO).

We also investigated associations in models conditioned on the matching variables but not further adjusted and associations in study-specific tenths. For comparison with previous analyses using the EHNBPCCG, estimates for trend were also defined using the study-specific fifths of the biomarker concentrations scored as 0, 0.25, 0.5, 0.75, and 1 and entered into the model as a continuous variable, therefore a unit increase in this variable can be taken to represent an 80 percentile increase in the biomarker study-specific concentration.

Associations with prostate cancer were also examined following mutual adjustment for the other analytes (IGF-I-2, IGFBP-1-3, free and total testosterone and SHBG), which were standardised by study (continuous). We additionally tested for interaction between the biomarkers by study-specific median concentrations, using a χ^2-test of interaction. Stratified analyses and associations in tenths were not investigated for early-onset disease due to the limited number of cases.

ii. Mendelian randomization analyses

Genetic instruments for hormone concentrations
Summary GWAS results for free and total testosterone and SHBG in men, using the UK Biobank resource, were extracted from a published analysis (up to 194,453 men)[40]. For free and total testosterone, the investigators used the inverse normal transformation of rank and adjusted for 10 principal components, fasting time, age, centre, chip/release of genetic data. For SHBG, the investigators used the natural log transformation and adjusted for 10 principal components, age, BMI, batch, dilution. To prevent possible issues related to collider bias[41], for MR analyses genetic instruments were identified using the BMI-adjusted estimates, but weights were assigned using the GWAS results that were not adjusted for BMI, as described previously[40].

UK Biobank genotyping details are reported elsewhere[42]. To ensure SNPs were independent, SNPs were pruned by a linkage disequilibrium (LD) threshold of $r^2<0.001$, based on the lowest p-value.

Genetic associations with prostate cancer
For each of the SNPs included as an instrument for free testosterone, total testosterone and SHBG, we obtained the association with prostate cancer from fixed-effects meta-analyses based on individuals of European ancestry in the PRACTICAL (Prostate Cancer Association Group to Investigate Cancer-Associated Alterations in the Genome) (including GAME-ON/ELLIPSE (Genetic Associations and Mechanisms in Oncology, Elucidating Loci Involved in Prostate Cancer Susceptibility))[43, 44]; genetic data for UK Biobank participants were not currently included in this dataset. Associations with overall prostate cancer were generated from 79,148 prostate cancer cases and 61,106 controls, aggressive disease from 15,167 aggressive cases and 58,308 controls, and early-onset disease from 6,988 cases and 44,256 controls[43, 44].

Statistical analysis
We used a 2-sample MR approach to estimate free and total testosterone, and SHBG associations with overall, aggressive, and early-onset prostate cancer risk, using UK Biobank as our source of genetic instruments for hormones and PRACTICAL for genetic outcome analyses.

The MR estimation for hormones was conducted using the multiplicative random effects inverse-variance weighted (IVW) method[45]. We additionally calculated the I^2 statistic to assess measurement error in SNP-exposure associations[46] and Cochran's Q statistic for heterogeneity between the MR estimates for each SNP[47]. PhenoScanner was used to assess pleiotropy of the genetic instruments[1]. As sensitivity analyses, we used the MR residual sum and outlier (MR-PRESSO) and MR robust adjusted profile score (MR-RAPS) to investigate the role of SNP outliers[48], and the weighted median, MR-Egger and the MR-Egger intercept to investigate horizontal pleiotropy[49, 50]. We also used the contamination mixture method, which assumes a normal distribution of valid instruments around the true causal value, and invalid instruments are normally distributed around zero in order to account for potentially pleiotropic variants[51].

For SHBG, we additionally investigated associations of the *cis*-SNP with prostate cancer risk, defined as the lead SNP identified from the exposure dataset in the SHBG gene-coding region. This *cis*-SNP may be less likely to be affected by horizontal pleiotropy than *trans*-SNPs[52]. Associations of this single SNP with prostate cancer were assessed using the Wald ratio.

Statistical software

Blood-based analyses were performed using Stata version 14.1 (Stata Corporation, College Station, TX, USA). MR analyses were performed using the *TwoSampleMR* R package (version 0.4.2)[53] and figures were plotted in R version 3.6.3. All tests of significance were two-sided, and P-values <0.05 were considered statistically significant.

Supplementary Table 1: Sample populations, recruitment and case ascertainment methods

Study	Sample population	Location	Recruitment period	Age at blood collection (years)	Prostate cancer ascertainment method
ATBC	Randomised trial of α-tocopherol and β-carotene among smokers	Finland	1985-1988	50-69	Cancer registry linkage, central review of medical records and specimens
BLSA	Prospective cohort study of the physiology of aging	USA	1958-onward	30-84	Self-report with medical record review
CARET	Randomised controlled trial of β-carotene and retinyl palmitate in heavy smokers and asbestos workers	USA	1987-1998	47-77	Self-report with medical record review
CHDS	Prospective cohort study	USA	1959-1966	20-55	Cancer registry linkage
CLUE	Prospective cohort study	USA	1974-onward	44-87	Cancer registry linkage
EPIC	Prospective cohort study	Europe	1991-2001	43-76	Cancer registry linkage; health insurance record linkage; self-report with medical record review
FMC	Prospective cohort study	Finland	1966-1972	17-80	Cancer registry linkage
HHS NBSBWG	Randomised controlled trial of gemfibrozil	Finland	1981-1982	40-56	Cancer registry linkage
HIMS	Population-based cohort study	Australia	1996-1999 (Blood collection 2001-2004)	71-87	Cancer registry linkage
HPFS	Cohort study of male dentists, optometrists, osteopathic physicians, podiatrists, pharmacists, and veterinarians	USA	1986	46-87	Self-report with medical record review
JACC	Prospective cohort study	Japan	1988-1990	57-85	Cancer registry linkage
JHCS	Prospective cohort study	USA	1971-1975	52-74	Surveillance of all general hospitals on Oahu, confirmed by biopsy/surgery and cancer registry linkage
JPHC	Prospective cohort study	Japan	Cohort 1)1990-1992 Cohort 2)1993-1995	41-71	Active patient notification from major local hospitals in the study area and linkage with population-based cancer registries.
Janus	Prospective cohort study	Norway			Cancer registry linkage
Janus NBSBWG	Prospective cohort study	Norway	1973-onward	33-61	Cancer registry linkage
MCCS	Prospective cohort study	Australia	1990-1994	40-72	Cancer registry linkage
MEC	Prospective cohort study	USA	1993-1996 (Blood collection 2001-2006)	48-85	Cancer registry linkage
MMAS	Prospective cohort study of the physiology of aging	USA	1987-1989	41-70	Cancer registry linkage
NSHDC	Combination of a population-based intervention study to decrease cardiovascular disease and a	Sweden	1985-onward	39-61	Cancer registry linkage

	population-based monitoring study of cardiovascular disease				
PCPT	Randomised, placebo-controlled trial of finasteride and prostate cancer	USA	1994-1997	55-83	Diagnosed as part of trial protocol. Annual digital rectal examinations and PSA measurements. Biopsy if abnormal DRE or reported PSA level > 4.0 ng per. End-of-study prostate biopsy
PHS	Randomised trial of aspirin and β-carotene among physicians	USA	1982-onward	41-78	Self-report with medical record review
PLCO	Randomised controlled multicentre trial for early detection of cancer of the prostate, lung, colorectum and ovary	USA	1993-2001	55-74	Medical and pathology record review after screening and self-report with medical record review
RBS	Prospective cohort study	USA	1984-1986	47-86	Self-report or death certificate, cancer deaths after 1988 were confirmed by the California Cancer Registry
UK Biobank	Prospective cohort study	UK	2006-2010	40-69	Cancer registry linkage

Abbreviations: ATBC=The Alpha-Tocopherol, Beta-Carotene Cancer Prevention Study; BLSA= The Baltimore Longitudinal Study of Aging; CARET =The Carotene and Retinol Efficacy Trial; CHDS=Child Health and Development Studies; CLUE=Give Us a Clue to Cancer and Heart Disease; DRE= digital rectal exam; EPIC=European Prospective Investigation into Cancer and Nutrition; FMC=Finnish Mobile Clinic Health Examination Survey*;* HHS= Helsinki Heart Study; HIMS=Health In Men Study; HPFS= Health Professionals Follow-up Study; JACC=Japan Collaborative Cohort Study; JPHC= Japan Public Health Center-based Prospective Study; JHCS= Japan-Hawaii Cancer Study; MCCS=Melbourne Collaborative Cohort Study; MEC= Multiethnic Cohort Study of Diet and Cancer; MMAS=Massachusetts Male Aging Study; NBSBWG=Nordic Biological Specimen Biobank Working Group; NSHDC=Northern Sweden Health and Disease Cohort; PCPT= Prostate Cancer Prevention Trial; PHS=Physicians' Health Study; PLCO= Prostate, Lung, Colorectal and Ovarian Cancer Screening Trial; PSA=prostate-specific antigen; RBS=Rancho Bernardo Study

Supplementary Table 2: Criteria used by individual studies to match case patients and control subjects

Study	Case:control ratio	Age at recruitment	Date of recruitment	Time of blood draw	Other matching criteria
ATBC	1:2	±1 y	±28 days		Trial intervention group, study centre
BLSA*	1:1	±6 mo			Follow-up time
CARET	1:1	±5 y	Month from enrolment to blood draw, year of randomisation	±2 h	Study centre, ethnicity
CHDS	1:2	±1 y			Ethnicity
CLUE	1:2	±1 y	±3 weeks		Ethnicity
EPIC phases 1-4	1:1 except for Umea centre (1:2)	±6 mo		±1 h	Recruitment centre, time between blood draw and last food or drink consumption, follow-up time
EPIC- Norfolk	1:2	±3 y	±3 mo		Follow-up time
FMC	1:2	Nearest available	±1 mo		Municipality
HHS NBSBWG	1:4	±2 y	±2 mo		Study centre
HIMS*	1:4	±1 y			±1 y date of blood collection
					Fasting status, diabetes, controls must be 'alive and at risk' beyond the case's date of diagnosis
HPFS	1:1	Year of birth ±1 y	Exact year	Midnight–9 am; 9 am–12 pm; 12 pm–4 pm; and 4 pm–midnight	PSA test before blood draw, season. control subjects had at least one PSA test after the date of blood draw
JACC	1:3	As close as possible			Recruitment area
JHCS	1:1	Same age	Same month or year	Same hour	
JPHC	1:2	±3 y	±60 days	±3 h	Municipality, duration of fasting at blood collection (±3 h)
Janus	1:3	±1 y	±6 mo		
Janus NBSBWG	1:4	±2 y	±6 mo		County of residence and Red Cross blood donor status (Oslo)
MCCS*	1:3	±5 y	±2 y		Assay batch, country of birth
MEC	1:2	±1 y	±6 mo	±2 h	Geographic site, ethnicity, fasting status (<6, 6-7, 8-9, 10+ hours)
MMAS*	1:4	±2 y	± 2 y		Ethnicity
NSHDC	1:2	±6 mo	±2 mo		County of residency
PCPT	1:1	As close as possible	As close as possible		PCPT treatment arm (placebo only). All non-whites controls were sampled and then backfilled with

					whites to achieve frequency matching on age and family history. Controls were required to have completed end of study biopsy procedure.
PHS	1:2	±1 y			Had not had a total or partial prostatectomy and smoking status
PLCO	1:1	±5 y	Exact year		Follow-up time
RBS	1:3	±2 y	±18 mo	±2 h	Race and smoking status
UK Biobank*	1:4	±18 mo	±18 mo	±2 h	No more than 2 hours difference in time since last meal, ethnicity

*Used a case–cohort design that was subsequently converted into nested case–control design.

Abbreviations: ATBC=The Alpha-Tocopherol, Beta-Carotene Cancer Prevention Study; BLSA= The Baltimore Longitudinal Study of Aging; CARET =The Carotene and Retinol Efficacy Trial; CHDS=Child Health and Development Studies; CLUE=Give Us a Clue to Cancer and Heart Disease; DRE= digital rectal exam; EPIC=European Prospective Investigation into Cancer and Nutrition; FMC= Finnish Mobile Clinic Health Examination Survey; HHS= Helsinki Heart Study; HIMS=Health In Men Study; HPFS= Health Professionals Follow-up Study; JACC= Japan Collaborative Cohort Study; JPHC= Japan Public Health Center-based Prospective Study; JHCS= Japan-Hawaii Cancer Study; MCCS=Melbourne Collaborative Cohort Study; MEC= Multiethnic Cohort Study of Diet and Cancer; MMAS=Massachusetts Male Aging Study; NBSBWG=Nordic Biological Specimen Biobank Working Group; NSHDC=Northern Sweden Health and Disease Cohort; PCPT= Prostate Cancer Prevention Trial; PHS=Physicians' Health Study; PLCO= Prostate, Lung, Colorectal and Ovarian Cancer Screening Trial; PSA=prostate-specific antigen; RBS=Rancho Bernardo Study.

Supplementary Table 3: Assay methods and geometric mean biomarker concentrations by study

Study		Testosterone (nmol/L) Method	CV %	Mean (SD)	SHBG (nmol/L) Method	CV %	Mean (SD)	Estimated free testosterone (pmol/L) Mean (SD)
ATBC	Case	E RIA	5.5†	21(7.5)	IRMA	4.2†	87(29)	228(62)
	Control			21(6.5)			90(31)	225(55)
BLSA	Case	NE RIA	3.3-6.4§	15(3.9)	NE RIA	1.8-22§	80(25)	178(56)
	Control			16(3.7)			85(27)	171(52)
CARET	Case	E RIA	1-12§	14(5)	IRMA	2-7§	30(13)	313(88)
	Control			15(5.6)			30(13)	331(101)
CHDS	Case	E RIA	9-11‡	23(8.2)	IA	4-6‡	35(14)	504(168)
	Control			24(8.5)			36(15)	505(174)
CLUE	Case	RIA	8*	16(6.9)	RIA	N/A	48(34)	
	Control			16(8.9)			47(24)	
EPIC Phase 1	Case	NE RIA	10.8-14.8†	17(7.4)	IRMA	7.7-12.2†	45(20)	309(138)
	Control			17(7)			47(21)	294(123)
EPIC Phase 2	Case	ECIA	2.7-6.1‡	16(5.8)	ECIA	2.0-2.7‡	54(22)	244(73)
	Control			16(5.7)			56(24)	239(68)
EPIC Phase 3/4	Case	ECIA	1.8-9.3‡	16(5.5)	ECIA	1.5-3.4‡	50(20)	259(77)
	Control			16(5.9)			52(22)	255(77)
EPIC Norfolk	Case	ECIA	7.1-12.0§	16(4.9)	ECIA	4.6-5.7§	43(14)	295(86)
	Control			17(5.7)			47(19)	296(91)
FMC	Case	NE RIA	4.5-7.2‡	25(9.9)	IMF	6.6-8.7‡	56(24)	408(140)
	Control			25(9.7)			56(25)	406(163)
HHS	Case	NE RIA	5.5-13§	21(5.9)	IMF	1.3-10.1§	57(28)	328(91)
	Control			21(6.7)			56(27)	328(102)
HIMS	Case	LC-MS/MS	<6‡	13(4.4)	IA	<7‡	41(15)	232(67)
	Control			13(4.9)			43(16)	228(77)
HPFS	Case	ECIA	≤5.2†¶	17(6.2)	IRMA	≤11.5†¶	74(51)	242(143)
	Control			17(5.9)			74(51)	238(137)
JACC	Case	NE RIA	5-12*	17(4.6)	IRMA	5.6-6.9*	44(18)	300(86)
	Control			16(4.7)			47(16)	278(78)
Janus	Case	E RIA	5-15§	20(6.1)				
	Control			20(7.5)				
Janus NBSBWG	Case	E RIA	5.5-13.0§	23(7)	IMF	5-15§	52(23)	395(134)
	Control			24(8.2)			54(26)	405(158)
JHCS	Case	E RIA	5-15§	19(7.4)	Precipitation	5-15§	39(12)	
	Control			19(7.9)			39(13)	
JPHC	Case	ECIA	1-3§	16(5.4)	IRMA	2-8§	50(18)	263(73)
	Control			17(5.5)			50(17)	266(78)
MCCS	Case	ECIA	1.6†	16(5.1)	IA	6 †	40(15)	304(84)
	Control			16(5.6)			39(15)	310(96)
MEC	Case	E RIA	3.5†	20(8)	ECIA	3†	38(16)	393(137)
	Control			20(7.6)			39(16)	390(136)
MMAS	Case	E RIA	4.6-7.2§	18(5.9)	Filtration assay	8-10.9§	34(18)	385(151)
	Control			17(5.8)			34(16)	377(138)
NSHDC	Case	N/S		22(8.1)	IRMA	N/S	46(20)	395(161)
	Control			21(9.3)			45(20)	390(169)
PCPT	Case	ECIA	7.6-11.9§	13(4.7)	ECIA	5.2-12.2§	39(15)	245(74)
	Control			13(4.5)			39(16)	242(71)
PHS	Case	NE RIA	8.7†	17(9.1)	IRMA	8.9†	23(12)	455(320)
	Control			17(6.2)			24(13)	433(163)
PLCO	Case	NE RIA	14*	18(7.9)	IRMA	18*	48(24)	299(119)
	Control			18(8.7)			49(23)	292(122)
RBS	Case	E RIA	4.1-10§	11(4)				
	Control			11(4.1)				
UK Biobank	Case	ECIA	3.7-8.3§	12(3.5)	ECIA	5.2-5.7§	42(16)	209(58)
	Control			12(3.8)			43(18)	208(61)

Abbreviations: ATBC=The Alpha-Tocopherol, Beta-Carotene Cancer Prevention Study; BLSA= The Baltimore Longitudinal Study of Aging; CARET =The Carotene and Retinol Efficacy Trial; CHDS=Child Health and Development Studies; CIA=chemiluminescence immunoassay; CLUE=Give Us a Clue to Cancer and Heart Disease; CV=coefficient of variation; E RIA=extraction radioimmunoassay; ECIA= electrochemiluminescence immunoassay; EPIC=European Prospective Investigation into Cancer and Nutrition; FMC= Finnish Mobile Clinic Health Examination Survey; HHS=Helsinki Heart Study; HIMS=Health In Men Study; HPFS= Health Professionals Follow-up Study; IA=immunoassay; IMF=immunofluorometry; IRMA= immunoradiometric assay; JACC= Japan Collaborative Cohort Study; JPHC= Japan Public Health Center-based Prospective Study; JHCS= Japan-Hawaii Cancer Study; LC-MS/MS= Liquid chromatography-tandem mass spectrometry; MCCS=Melbourne Collaborative Cohort Study; MEC=Multiethnic Cohort Study of Diet and Cancer; MMAS=Massachusetts Male Aging Study; NBSBWG=Nordic Biological Specimen Biobank Working Group; NE RIA=non-extraction radioimmunoassay; NSHDC=Northern Sweden Health and Disease Cohort; PCPT= Prostate Cancer Prevention Trial; PHS=Physicians' Health Study; PLCO= Prostate, Lung, Colorectal and Ovarian Cancer Screening Trial; RBS=Rancho Bernardo Study; SD=standard deviation.

* Not specified

† Intra-assay

‡ Inter-assay

§Intra-and inter-assay range

Supplementary Table 4: Prostate cancer characteristics in participating EHNBPCCG studies

Study	Years blood collection to diagnosis, %		Age at diagnosis, %		Year of diagnosis, %		Aggressive disease*, %			Disease stage†, %			Disease grade‡, %			Prostate cancer death, %
	<5 years	5+ years	≤55 years	>55 years	pre 1995	1995 onwards	No	Yes	Unknown[a]	Localized	Advanced	Unknown[a]	Low	High	Unknown[a]	Yes
ATBC	84 (72)	32 (28)	6 (5)	110 (95)	116 (100)	0 (0)	49 (43)	66 (57)	1 (1)	71 (61)	45 (39)	0 (0)	90 (80)	22 (20)	4 (3)	59 (51)
BLSA	17 (15)	95 (85)	4 (4)	108 (96)	76 (68)	36 (32)	38 (72)	15 (28)	59 (53)	42 (79)	11 (21)	59 (53)	77 (87)	12 (13)	23 (21)	18 (16)
CARET	247 (83)	51 (17)	12 (4)	286 (96)	132 (44)	166 (56)	200 (76)	64 (24)	34 (11)	189 (72)	75 (28)	34 (11)	246 (88)	34 (12)	18 (6)	33 (11)
CHDS	0 (0)	322 (100)	21 (7)	301 (93)	142 (44)	180 (56)	249 (91)	26 (9)	47 (15)	229 (80)	57 (20)	36 (11)	299 (99)	4 (1)	19 (6)	32 (10)
CLUE	37 (40)	56 (60)	1 (1)	92 (99)	93 (100)	0 (0)	37 (51)	35 (49)	21 (23)	46 (73)	17 (27)	30 (32)	65 (89)	8 (11)	20 (22)	29 (31)
EPIC phase 1	471 (77)	141 (23)	38 (6)	574 (94)	6 (1)	606 (99)	239 (57)	180 (43)	193 (32)	291 (69)	130 (31)	191 (31)	402 (89)	51 (11)	159 (26)	141 (23)
EPIC phase 2	75 (13)	497 (87)	28 (5)	544 (95)	0 (0)	572 (100)	260 (68)	124 (32)	188 (33)	318 (76)	102 (24)	152 (27)	371 (88)	50 (12)	151 (26)	85 (14)
EPIC phase 3/4	15 (1)	1,588 (99)	64 (4)	1,539 (96)	0 (0)	1,603 (100)	676 (70)	285 (30)	642 (40)	815 (76)	260 (24)	528 (33)	972 (87)	140 (13)	491 (31)	114 (7)
EPIC-Norfolk	10 (13)	65 (87)	0 (0)	75 (100)	0 (0)	75 (100)	0 (0)	33 (100)	41 (55)	52 (75)	17 (25)	6 (8)	5 (71)	2 (29)	68 (91)	28 (33)
FMC	22 (13)	144 (87)	8 (5)	158 (95)	166 (100)	0 (0)	0 (0)	57 (100)	109 (66)	N/A	N/A	166 (100)	N/A	N/A	166 (100)	70 (42)
HHS NBSBWG	6 (7)	78 (93)	7 (8)	77 (92)	64 (76)	20 (24)	0 (0)	25 (100)	59 (70)	41 (62)	25 (38)	18 (21)	N/A	N/A	84 (100)	0 (0)
HIMS	212 (66)	109 (34)	0 (0)	321 (100)	0 (0)	321 (100)	0 (0)	36 (100)	285 (89)	N/A	N/A	321 (100)	N/A	N/A	321 (100)	36 (11)
HPFS	564 (83)	118 (17)	46 (7)	636 (93)	85 (12)	597 (88)	503 (83)	102 (17)	77 (11)	608 (95)	32 (5)	42 (6)	552 (91)	54 (9)	76 (11)	80 (12)
JACC	18 (45)	22 (55)	0 (0)	40 (100)	22 (55)	18 (45)	0 (0)	2 (100)	38 (95)	N/A	N/A	40 (100)	N/A	N/A	40 (100)	0 (0)
JHCS 1988	28 (29)	70 (71)	1 (1)	97 (99)	98 (100)	0 (0)	0 (0)	32 (100)	66 (67)	51 (53)	45 (47)	2 (2)	N/A	N/A	98 (100)	0 (0)
JHCS 1996	16 (12)	122 (88)	0 (0)	138 (100)	138 (100)	0 (0)	0 (0)	29 (100)	109 (79)	96 (70)	42 (30)	0 (0)	N/A	N/A	138 (100)	0 (0)
JPHC	39 (19)	162 (81)	2 (1)	199 (99)	7 (3)	194 (97)	16 (23)	53 (77)	132 (66)	108 (72)	43 (28)	50 (25)	47 (76)	15 (24)	139 (69)	3 (1)
Janus	13 (22)	47 (78)	1 (2)	59 (98)	60 (100)	0 (0)	N/A	N/A	60 (100)	N/A	N/A	60 (100)	N/A	N/A	60 (100)	0 (0)
Janus NBSBWG	17 (3)	520 (97)	50 (9)	487 (91)	445 (83)	92 (17)	N/A	N/A	537 (100)	N/A	N/A	537 (100)	N/A	N/A	537 (100)	0 (0)
MCCS	229 (41)	328 (59)	27 (5)	530 (95)	86 (15)	471 (85)	415 (76)	129 (24)	13 (2)	498 (91)	52 (9)	7 (1)	469 (86)	74 (14)	14 (3)	109 (18)
MEC	434 (94)	30 (6)	6 (1)	458 (99)	0 (0)	464 (100)	0 (0)	49 (100)	415 (89)	N/A	N/A	464 (100)	442 (100)	1 (0)	21 (5)	49 (11)
MMAS	32 (20)	131 (80)	12 (7)	151 (93)	47 (29)	116 (71)	N/A	N/A	163 (100)	N/A	N/A	163 (100)	86 (100)	0 (0)	77 (47)	3 (2)
NSHDC	141 (37)	243 (63)	31 (8)	353 (92)	18 (5)	366 (95)	258 (77)	75 (23)	51 (13)	305 (80)	75 (20)	4 (1)	287 (90)	31 (10)	66 (17)	0 (0)
PCPT	238 (23)	794 (77)	0 (0)	1,032 (100)	4 (0)	1,028 (100)	929 (95)	53 (5)	50 (5)	988 (98)	17 (2)	27 (3)	958 (95)	49 (5)	25 (2)	4 (0)
PHS	66 (30)	153 (70)	11 (5)	208 (95)	219 (100)	0 (0)	130 (63)	76 (37)	13 (6)	167 (78)	46 (22)	6 (3)	184 (91)	18 (9)	17 (8)	67 (30)
PLCO	648 (89)	79 (11)	0 (0)	728 (100)	0 (0)	728 (100)	522 (89)	66 (11)	0 (0)	636 (89)	79 (11)	0 (0)	681 (95)	35 (5)	1 (0)	30 (4)
RBS	36 (35)	68 (65)	1 (1)	103 (99)	64 (62)	40 (38)	0 (0)	21 (100)	82 (80)	N/A	N/A	104 (100)	N/A	N/A	104 (100)	22 (21)
UK Biobank	3,707 (67)	1,824 (33)	252 (5)	5,279 (95)	0 (0)	5,531 (100)	0 (0)	189 (100)	5,342 (97)	N/A	N/A	5,531 (100)	N/A	N/A	5,531 (100)	313 (6)

*Aggressive disease was defined as Gleason Score 8+, death from prostate cancer, metastatic disease, or PSA>100 ng/mL. Non-aggressive disease is defined as Gleason score <8 and non-metastatic disease.

†Stage of disease was defined as follows: localised if TNM was T2 or lower with no reported lymph node involvement or metastases, stage II or lower, or equivalent (ie, a tumour that does not extend beyond the prostate capsule); advanced if TNM stage was T3 or T4 and/or N1+ and/or M1, stage III or IV, equivalent (ie, a tumour extending beyond the prostate capsule and/or lymph node involvement and/or distant metastases), or unknown.

‡Histological grade was categorised as low-intermediate grade (Gleason sum <8 or cases coded as well, moderately, or poorly differentiated), high grade (Gleason sum 8+ or cases coded as undifferentiated), or unknown.

[a]Percentage value is for those with known disease characteristics.

Abbreviations: ATBC=The Alpha-Tocopherol, Beta-Carotene Cancer Prevention Study; BLSA= The Baltimore Longitudinal Study of Aging; CARET =The Carotene and Retinol Efficacy Trial; CHDS=Child Health and Development Studies; CLUE=Give Us a Clue to Cancer and Heart Disease; EHNBPCCG=Endogenous Hormones, Nutritional Biomarkers and Prostate Cancer Collaborative Group; EPIC=European Prospective Investigation into Cancer and Nutrition; FMC= Finnish Mobile Clinic Health Examination Survey; HHS= Helsinki Heart Study; HIMS=Health In Men Study; HPFS= Health Professionals Follow-up Study; JACC= Japan Collaborative Cohort Study; JPHC= Japan Public Health Center-based Prospective Study; JHCS= Japan-Hawaii Cancer Study; MCCS=Melbourne Collaborative Cohort Study; MEC=Multiethnic Cohort Study of Diet and Cancer; MMAS=Massachusetts Male Aging Study; NBSBWG=Nordic Biological Specimen Biobank Working Group; NSHDC=Northern Sweden Health and Disease Cohort; PCPT= Prostate Cancer Prevention Trial; PHS=Physicians' Health Study; PLCO= Prostate, Lung, Colorectal and Ovarian Cancer Screening Trial; PSA=prostate-specific antigen; RBS=Rancho Bernardo Study.

Supplementary Table 5: Partial correlation coefficients of circulating biomarkers in prostate cancer controls*

	IGF-I	IGF-II	IGFBP-1	IGFBP-2	IGFBP-3	Total testosterone	SHBG	Free testosterone	PSA
IGF-I	1								
IGF-II	0.42[b]	1							
IGFBP-1	0.14[b]	0.43[b]	1						
IGFBP-2	0.24[b]	0.54[b]	0.71[b]	1					
IGFBP-3	0.50[b]	0.45[b]	-0.13[b]	-0.16[b]	1				
Total testosterone	0.02[a]	-0.21[b]	0.20[b]	0.05[a]	-0.09[b]	1			
SHBG	-0.20[b]	-0.27[b]	0.03	0.03	-0.21[b]	0.49[b]	1		
Free testosterone	0.17[b]	-0.04	0.21[b]	0.03	0.07[b]	0.77[b]	-0.17[b]	1	
PSA	-0.03[a]	-0.004	0.04[a]	0.06[b]	0.01	0.08[b]	-0.04[b]	0.11[b]	1

* Biomarkers were log-transformed and adjusted for age (5-year groups) and BMI (<25, 25-27.4, 27.5-29.9, 30+ kg/m^2, unknown)

[a] P <0.05

[b] P <0.001

Abbreviations: IGF=insulin-like growth factor; IGFBP=insulin-like growth factor binding protein; PSA=prostate-specific antigen; SHBG=sex hormone binding globulin

Supplementary Table 6: Risks of overall, aggressive and early-onset prostate cancer per 80% tile increment in biomarker concentrations in the EHNBPCCG studies

	Overall prostate cancer			Aggressive prostate cancer*			Early-onset prostate cancer†		
	Cases/Controls	OR per 80%tile increment (95% CI)	P_{trend}	Cases/Controls	OR per 80%tile increment (95% CI)	P_{trend}	Cases/Controls	OR per 80%tile increment (95% CI)	P_{trend}
Free testosterone	14112/34347	1.09 (1.02, 1.16)	0.01	1756/3327	0.88 (0.73, 1.07)	0.22	588/1579	1.41 (1.02, 1.95)	0.04
Total testosterone	14944/36752	0.91 (0.86, 0.97)	0.003	1870/3527	0.87 (0.73, 1.04)	0.12	611/1665	0.94 (0.70, 1.26)	0.68
SHBG	14474/35038	0.80 (0.76, 0.86)	<0.0001	1835/3419	0.91 (0.76, 1.11)	0.36	605/1611	0.64 (0.46, 0.90)	0.01

Estimates are from logistic regression conditioned on the matching variables and adjusted for age, BMI, height, alcohol intake, smoking status, marital status, education status, racial/ethnic group, and diabetes status. The categorical variables representing the study-specific fifths of the biomarker concentrations was replaced with a continuous variable that was scored as 0, 0.25, 0.5, 0.75, and 1; because the mid-points of the lowest and highest fifths are the 10th and 90th percentiles of the study-specific biomarker concentrations, a unit increase in this variable can be taken to represent an 80 percentile increase in the biomarker study-specific concentration.

*Aggressive cancer defined as Gleason grade 8+, or prostate cancer death, or metastases or PSA>100 ng/mL.

†Early-onset defined as diagnosed ≤ 55 years.

Abbreviations: BMI=body mass index; CI=confidence interval; EHNBPCCG=Endogenous Hormones, Nutritional Biomarkers and Prostate Cancer Collaborative Group; OR=odds ratio; PSA=prostate-specific antigen; SHBG=sex hormone–binding globulin.

Supplementary Table 7: Risks of overall, aggressive and early-onset prostate cancer per study-specific 1 SD increment in biomarker concentrations with mutual adjustment, among cases and their matched controls in EHNBPCCG studies

	Overall prostate cancer			Aggressive prostate cancer*			Early-onset prostate cancer†		
	Cases /Controls	OR (95% CI)	P_{trend}	Cases/ Controls	OR (95% CI)	P_{trend}	Cases/ Controls	OR (95% CI)	P_{trend}
**Free testosterone alone**	**14112/34347**	**1.03 (1.01, 1.05)**	**0.01**	**1756/3327**	**0.96 (0.90, 1.03)**	**0.29**	**588/1579**	**1.08 (0.98, 1.19)**	**0.13**
Adjusted for IGF-I	11885/29261	1.03 (1.00, 1.05)	0.03	1349/2607	0.95 (0.87, 1.03)	0.20	453/1228	1.10 (0.99, 1.23)	0.08
Adjusted for IGF-II	3645/4027	1.06 (1.01, 1.12)	0.02	524/546	1.00 (0.86, 1.16)	0.97	98/102	1.16 (0.81, 1.67)	0.42
Adjusted for IGFBP-1	2986/4274	1.06 (1.00, 1.11)	0.05	531/626	1.05 (0.91, 1.22)	0.51	100/104	1.14 (0.80, 1.63)	0.46
Adjusted for IGFBP-2	3133/3130	1.08 (1.02, 1.15)	0.01	442/442	1.09 (0.93, 1.27)	0.29	85/85	1.23 (0.83, 1.82)	0.31
Adjusted for IGFBP-3	6505/8472	1.06 (1.02, 1.10)	0.003	1035/1444	1.02 (0.92, 1.13)	0.67	201/272	1.38 (1.10, 1.73)	0.01
**Total testosterone alone**	**14944/36752**	**0.96 (0.94, 0.99)**	**0.0009**	**1870/3527**	**0.95 (0.89, 1.01)**	**0.09**	**611/1665**	**0.96 (0.87, 1.07)**	**0.48**
Adjusted for IGF-I	12344/31008	0.97 (0.95, 0.99)	0.009	1384/2712	0.93 (0.87, 1.01)	0.07	473/1307	1.00 (0.89, 1.12)	0.98
Adjusted for IGF-II	3649/4032	0.99 (0.94, 1.04)	0.62	525/548	0.99 (0.86, 1.14)	0.90	99/103	1.19 (0.80, 1.76)	0.39
Adjusted for IGFBP-1	2990/4280	0.97 (0.92, 1.02)	0.26	532/628	1.04 (0.91, 1.19)	0.55	101/105	1.13 (0.78, 1.65)	0.51
Adjusted for IGFBP-2	3137/3134	0.99 (0.94, 1.05)	0.80	443/443	1.08 (0.93, 1.26)	0.32	86/86	1.29 (0.83, 2.01)	0.26
Adjusted for IGFBP-3	6553/8521	1.00 (0.97, 1.04)	0.91	1048/1458	1.00 (0.91, 1.09)	0.98	202/273	1.39 (1.10, 1.76)	0.01
**SHBG alone**	**14474/35038**	**0.91 (0.89, 0.93)**	**<0.0001**	**1835/3419**	**0.97 (0.91, 1.04)**	**0.40**	**605/1611**	**0.83 (0.74, 0.95)**	**0.005**
Adjusted for IGF-I	12077/29665	0.91 (0.89, 0.94)	<0.0001	1388/2658	0.98 (0.91, 1.06)	0.58	468/1254	0.83 (0.72, 0.96)	0.01
Adjusted for IGF-II	3711/4094	0.92 (0.87, 0.97)	0.0012	541/563	0.99 (0.87, 1.13)	0.89	104/108	1.20 (0.76, 1.88)	0.44
Adjusted for IGFBP-1	3053/4347	0.90 (0.85, 0.95)	0.0001	548/645	1.02 (0.89, 1.16)	0.79	106/110	1.12 (0.71, 1.76)	0.63
Adjusted for IGFBP-2	3198/3195	0.90 (0.85, 0.96)	0.0008	459/459	0.99 (0.84, 1.15)	0.86	91/91	1.31 (0.74, 2.33)	0.36
Adjusted for IGFBP-3	6662/8649	0.93 (0.90, 0.97)	0.0006	1075/1489	1.00 (0.91, 1.10)	0.98	214/285	1.03 (0.79, 1.36)	0.81
Adjusted for total testosterone	14159/34689	0.90 (0.87, 0.92)	<0.0001	1756/3327	1.01 (0.93, 1.10)	0.73	591/1599	0.77 (0.66, 0.90)	0.001

Estimates are from logistic regression conditioned on the matching variables and adjusted for age, BMI, height, alcohol intake, smoking status, marital status, education status, racial/ethnic group, and diabetes status. Estimates were mutually adjusted for biomarkers per study-specific 1 SD entered as a continuous variable.

Abbreviations: BMI=body mass index; CI=confidence interval; EHNBPCCG=Endogenous Hormones, Nutritional Biomarkers and Prostate Cancer Collaborative Group; IGF=insulin-like growth factor; IGFBP=insulin-like growth factor binding protein; OR=odds ratio; SD=standard deviation.

Supplementary Table 8: Risks of overall prostate cancer in relation to biomarkers, stratified by other study-specific median biomarker concentrations in EHNBPCCG studies

		Free testosterone			Total testosterone			SHBG		
		Cases/ controls	OR (95 % CI)	P_{het}	Cases/ controls	OR (95 % CI)	P_{het}	Cases/ controls	OR (95 % CI)	P_{het}
Free testosterone	< Median									
	Median +									
Total testosterone	< Median							7172/17152	0.92 (0.88, 0.96)	
	Median +							6940/17195	0.90 (0.87, 0.92)	0.24
SHBG	< Median				7379/17169	1.03 (0.99, 1.07)				
	Median +				6733/17178	0.97 (0.94, 1.00)	0.01			
IGF-I	< Median	5467/14419	1.05 (1.02, 1.09)		5677/15316	0.96 (0.93, 0.99)		5559/14602	0.90 (0.87, 0.93)	
	Median +	6384/14682	1.01 (0.98, 1.05)	0.09	6632/15528	0.97 (0.94, 1.00)	0.76	6479/14902	0.92 (0.89, 0.95)	0.42
IGF-II	< Median	1660/1972	1.08 (1.00, 1.16)		1662/1974	0.96 (0.90, 1.03)		1689/2004	0.89 (0.83, 0.95)	
	Median +	1976/2045	1.05 (0.98, 1.12)	0.53	1978/2048	1.02 (0.95, 1.09)	0.23	2013/2080	0.97 (0.90, 1.04)	0.08
IGFBP-1	< Median	1276/1899	1.04 (0.96, 1.13)		1277/1902	1.00 (0.92, 1.09)		1304/1923	0.96 (0.88, 1.05)	
	Median +	1703/2328	1.07 (1.00, 1.14)	0.59	1706/2331	0.95 (0.89, 1.01)	0.31	1742/2377	0.86 (0.81, 0.92)	0.04
IGFBP-2	< Median	1508/1616	1.08 (1.00, 1.17)		1508/1620	1.00 (0.92, 1.08)		1544/1644	0.90 (0.82, 0.98)	
	Median +	1619/1511	1.07 (0.99, 1.16)	0.91	1623/1511	0.97 (0.90, 1.04)	0.61	1648/1548	0.90 (0.84, 0.96)	0.97
IGFBP-3	< Median	3136/4218	1.08 (1.02, 1.14)		3163/4248	0.98 (0.93, 1.03)		3214/4309	0.91 (0.87, 0.95)	
	Median +	3332/4193	1.05 (1.00, 1.10)	0.37	3352/4212	1.01 (0.96, 1.07)	0.32	3407/4278	0.96 (0.90, 1.01)	0.17

Estimates are from logistic regression conditioned on the matching variables and adjusted for age, BMI, height, alcohol intake, smoking status, marital status, education status, racial/ethnic group, and diabetes status.

Abbreviations: BMI=body mass index; CI=confidence interval; EHNBPCCG=Endogenous Hormones, Nutritional Biomarkers and Prostate Cancer Collaborative Group; IGF=insulin-like growth factor; IGFBP=insulin-like growth factor binding protein; OR=odds ratio; SD=standard deviation; SHBG=sex hormone binding globulin.

Supplementary Table 9: Risks of aggressive prostate cancer in relation to biomarkers, stratified by other study-specific median biomarker concentrations in EHNBPCCG studies

		Free testosterone			Total testosterone			SHBG		
		Cases/ controls	OR (95 % CI)	P_{het}	Cases/ controls	OR (95 % CI)	P_{het}	Cases/ controls	OR (95 % CI)	P_{het}
Free testosterone	< Median									
	Median +									
Total testosterone	< Median							943/1704	1.05 (0.93, 1.18)	
	Median +							813/1623	0.96 (0.87, 1.05)	0.08
SHBG	< Median				890/1657	1.07 (0.94, 1.21)				
	Median +				866/1670	0.87 (0.79, 0.95)	0.01			
IGF-I	< Median	658/1322	0.95 (0.85, 1.07)		676/1383	0.91 (0.82, 1.01)		678/1345	0.93 (0.84, 1.02)	
	Median +	691/1285	0.95 (0.86, 1.05)	0.72	708/1329	0.96 (0.86, 1.07)	0.41	710/1313	1.04 (0.92, 1.16)	0.33
IGF-II	< Median	261/261	1.09 (0.88, 1.36)		262/262	1.02 (0.84, 1.24)		268/266	0.99 (0.84, 1.17)	
	Median +	263/285	0.94 (0.78, 1.14)	0.33	263/286	0.97 (0.80, 1.18)	1	273/297	1.01 (0.82, 1.24)	0.73
IGFBP-1	< Median	217/280	1.08 (0.84, 1.38)		217/281	1.22 (0.97, 1.54)		227/285	1.23 (1.00, 1.51)	
	Median +	314/346	1.03 (0.87, 1.22)	0.6	315/347	0.93 (0.79, 1.10)	0.06	321/360	0.85 (0.71, 1.02)	0.01
IGFBP-2	< Median	214/221	1.14 (0.93, 1.40)		214/222	1.09 (0.87, 1.36)		227/227	0.93 (0.70, 1.24)	
	Median +	228/221	1.01 (0.81, 1.26)	0.17	229/221	1.00 (0.82, 1.21)	0.3	232/232	0.94 (0.78, 1.14)	0.83
IGFBP-3	< Median	548/770	1.01 (0.87, 1.16)		556/778	0.99 (0.88, 1.11)		567/792	0.99 (0.89, 1.11)	
	Median +	487/674	1.04 (0.90, 1.19)	0.56	492/680	1.01 (0.88, 1.16)	0.33	508/697	1.00 (0.86, 1.17)	0.44

Estimates are from logistic regression conditioned on the matching variables and adjusted for age, BMI, height, alcohol intake, smoking status, marital status, education status, racial/ethnic group, and diabetes status.

Abbreviations: BMI=body mass index; CI=confidence interval; IGF=insulin-like growth factor; EHNBPCCG=Endogenous Hormones, Nutritional Biomarkers and Prostate Cancer Collaborative Group; IGFBP=insulin-like growth factor binding protein; OR=odds ratio; SD=standard deviation; SHBG=sex hormone-binding globulin.

Supplementary Table 10: Trait associations with the SHBG *cis*-SNP*

SNP	Positive association
SHBG (rs1799941)	Testosterone<br>Impedance of arm left<br>Impedance of arm right<br>Vascular or heart problems diagnosed by doctor: high blood pressure<br>Impedance of whole body<br>Self-reported hypertension

*Using PhenoScanner resource (P threshold=5 x 10^{-8})[1, 2]. Associations are orientated such that the effect allele is positively associated with protein expression.

Abbreviations: SHBG=sex hormone–binding globulin.

Supplementary Table 11: Association of free testosterone SNPs used in 2-sample Mendelian randomization analyses with prostate cancer

							Association parameters with free testosterone			Association parameters with overall prostate cancer			Association parameters with aggressive prostate cancer			Association parameters with early-onset prostate cancer		
SNP	Chr	Position	Consequence	Nearest gene	Effect allele	Other allele	Beta	SE	P-value	Beta	SE	P-value	Beta	SE	P-value	Beta	SE	P-value
rs72664935	1	32274901	intron	SPOCD1	C	T	0.019	0.003	1.70E-09	0.0154	0.0086	0.07481	0.0046	0.0148	0.7543	0.0039	0.0216	0.8554
rs71519251	1	163251833	intron	NUF2	A	G	0.024	0.004	1.50E-09	-0.0203	0.0105	0.05285	-0.0109	0.018	0.5456	-0.0245	0.0264	0.3545
rs35737316	1	204161534	-	-	T	C	0.04	0.004	4.70E-32	0.0017	0.0093	0.8552	-0.0075	0.0161	0.6427	-3.00E-04	0.0231	0.9886
rs13028479	2	11712075	intron	GREB1	G	T	0.031	0.003	2.70E-24	0.0143	0.0087	0.1017	0.0079	0.0151	0.5989	0.0089	0.0218	0.6823
rs829593	2	30641234	intergenic	RP11-182E7.1	G	A	0.018	0.003	1.50E-08	-0.0014	0.009	0.876	-0.0072	0.0155	0.6439	-0.0148	0.0224	0.5082
rs2438086	2	105871129	downstream	AC012360.1	G	A	0.022	0.003	6.90E-13	0.0202	0.0091	0.02634	0.0196	0.0155	0.2071	0.0229	0.023	0.32
rs6718154	2	180497923	intron	ZNF385B	T	C	0.032	0.003	1.20E-22	0.0011	0.0088	0.8978	-0.0077	0.0152	0.6111	0.0153	0.0219	0.4834
rs2011425	2	234627608	missense	UGT1A4	T	G	0.05	0.006	6.40E-20	0.0117	0.0147	0.4278	0.0054	0.0254	0.8299	0.0047	0.0365	0.8976
rs1112195	3	24085166	intergenic	CRIP1P2	G	A	0.018	0.003	4.70E-10	-0.003	0.0082	0.7172	0.009	0.014	0.523	-0.0183	0.0204	0.37
rs9824196	3	28807441	intron	RBMS3	T	G	0.026	0.003	4.10E-16	0.0096	0.009	0.2893	0.0075	0.0155	0.6296	0.0202	0.0226	0.3711
rs3821866	3	53805577	intron	CACNA1D	G	C	0.022	0.003	4.10E-14	-0.0052	0.0084	0.5324	0.0131	0.0145	0.3646	0.0211	0.0211	0.3172
rs10510939	3	65507808	intron	MAGI1	C	T	0.017	0.003	1.60E-08	0.0066	0.0083	0.4294	-0.0101	0.0143	0.4791	0.0135	0.0207	0.5155
rs34040779	3	107235109	intergenic	CD200R1L	T	C	0.035	0.006	2.20E-09	0.0794	0.0158	4.58E-07	0.0587	0.0271	0.03022	0.1415	0.0413	0.000613
rs4678408	3	138053187	upstream	NME9	G	A	0.026	0.003	7.60E-17	-0.0082	0.0088	0.3522	-0.0184	0.0151	0.2232	0.0059	0.0221	0.791
rs61762319	3	154801978	missense	MME	G	A	0.05	0.009	2.70E-08	0.0219	0.0355	0.5377	-0.0224	0.0612	0.7146	-0.0134	0.089	0.8807
rs7679843	4	22028079	intron	RP11-17E2.2	G	C	0.05	0.005	3.80E-22	0.0095	0.0143	0.5054	-0.0025	0.0248	0.9207	0.0182	0.0353	0.6055
rs4274916	4	69988378	upstream	RP13-644M16.1	C	T	0.017	0.003	3.20E-09	-0.0055	0.0081	0.4964	-0.0122	0.014	0.3851	0.0031	0.0202	0.8798
rs17254118	4	103651441	intron	MANBA	C	T	0.109	0.011	5.10E-24	0.0206	0.0371	0.5788	0.0323	0.0641	0.6149	0.1764	0.0894	0.04832
rs72774885	5	95840231	intron	CTD-2337A12.1	C	T	0.027	0.004	3.50E-10	-0.0202	0.013	0.1186	-0.0231	0.0224	0.302	-0.0412	0.0325	0.2054
rs950716	5	135680540	intron	TRPC7	A	G	0.034	0.004	3.10E-14	0.0122	0.012	0.3126	-0.002	0.0207	0.921	0.0181	0.0295	0.5385
rs2961853	5	165932048	intergenic	HMP19	C	T	0.019	0.003	3.20E-10	0.01	0.0087	0.2517	0.0253	0.0149	0.08851	0.0156	0.0218	0.4751
rs34192788	6	17416258	intron	CAP2	T	A	0.02	0.003	4.20E-09	0.0175	0.0095	0.06431	0.0285	0.0161	0.07553	0.0172	0.0238	0.4711
rs204995	6	32154285	intron	PBX2	A	G	0.027	0.003	3.20E-13	0.0502	0.0096	1.76E-07	0.0622	0.0166	0.0001741	0.0557	0.0232	0.01645
rs11751920	6	34655818	intron	C6orf106	G	C	0.061	0.01	4.60E-09	-0.0516	0.0301	0.08581	-0.075	0.0508	0.14	-0.0742	0.0754	0.3256
rs7454964	6	52728059	intron	GSTA11P	T	C	0.017	0.003	1.80E-08	-0.001	0.0081	0.8981	0.0199	0.014	0.1552	-0.0043	0.0202	0.8332
rs9322822	6	105369598	intron	LIN28B-AS1	C	T	0.049	0.003	2.10E-52	0.0078	0.0085	0.3532	-0.0211	0.0145	0.1458	0.0208	0.0209	0.3216
rs2184968	6	126760994	intergenic	RP11-394G3.2	C	T	0.02	0.003	9.10E-11	0.0094	0.008	0.2435	0.0028	0.0138	0.839	-0.041	0.02	0.04085
rs9986829	7	15019259	upstream	DGKB	A	G	0.056	0.003	2.80E-76	0.0011	0.0085	0.8949	0.0172	0.0145	0.2365	0.007	0.0211	0.7408
rs10279715	7	40870935	intron	SUGCT	A	G	0.022	0.003	3.10E-13	0.0412	0.0082	4.87E-07	0.0459	0.014	0.001002	0.0431	0.0204	0.03434
rs55795858	7	146123500	intron	CNTNAP2	C	T	0.018	0.003	1.50E-08	-0.0039	0.0094	0.6779	-0.0031	0.016	0.8443	-1.00E-04	0.0234	0.9972
rs2631864	8	21112084	intergenic	RP11-24P4.1	G	A	0.033	0.005	8.30E-12	0.0236	0.0131	0.07202	0.0378	0.0224	0.09162	0.0518	0.0322	0.1076
rs4872310	8	25247181	intron	DOCK5	G	A	0.023	0.004	3.70E-12	0.0036	0.0094	0.7018	-0.0111	0.0162	0.4931	0.0223	0.0237	0.3454
rs4562360	8	61704817	intron	CHD7	G	A	0.032	0.004	3.60E-20	0.0269	0.0095	0.004597	0.0372	0.0163	0.02256	0.0443	0.0233	0.05703
rs71529289	8	77879487	upstream	hsa-mir-3149	C	T	0.036	0.003	2.00E-25	8.00E-04	0.0097	0.9329	-0.0077	0.0166	0.642	0.0386	0.0247	0.1185
rs745486	9	11242155	intergenic	RP11-23D5.1	C	T	0.021	0.003	1.20E-10	-0.0029	0.0092	0.7516	0.0017	0.0158	0.9132	0.0349	0.0231	0.131
rs10738700	9	24973797	intergenic	RMRPP5	A	G	0.02	0.003	5.60E-11	0.0074	0.0085	0.3821	0.0239	0.0145	0.09955	-0.0069	0.0211	0.744
rs912202	9	77225603	intron	RORB	C	G	0.04	0.003	2.50E-38	0.0103	0.0087	0.2357	-0.0058	0.0149	0.698	0.0258	0.0217	0.2357
rs2090409	9	108967088	intron	LINC01505	C	A	0.031	0.003	1.70E-21	0.0015	0.0084	0.8582	0.0051	0.0144	0.7206	-0.0264	0.0208	0.2053
rs10982156	9	117088064	intron	ORM1	A	T	0.048	0.006	4.50E-14	-0.0259	0.0201	0.1992	-0.0418	0.0341	0.2209	0.0079	0.05	0.8742
rs7872329	9	131956152	intron	RP11-247A12.2	A	T	0.017	0.003	3.80E-08	0.0173	0.0093	0.06466	0.008	0.016	0.616	0.0585	0.0236	0.01327
rs7912521	10	67262089	intergenic	LINC01515	C	T	0.061	0.003	6.20E-94	-0.001	0.0082	0.9003	0.0053	0.0141	0.7085	-0.0136	0.0203	0.5039
rs4919686	10	104592249	intron	CYP17A1	A	C	0.023	0.003	3.90E-13	0.04	0.0088	5.45E-06	0.0413	0.0151	0.006366	0.0923	0.022	2.72E-05
rs7915430	10	121660465	intron	SEC23IP	T	G	0.021	0.004	9.70E-09	0.0026	0.0098	0.7913	-0.0024	0.0169	0.8853	-0.0176	0.0245	0.474
rs2035837	11	29200527	intron	RP11-466I1.1	T	C	0.073	0.004	6.70E-67	0.0149	0.0112	0.1819	0.0117	0.0193	0.546	0.0444	0.0281	0.1143
rs55765314	11	72360935	intron	PDE2A	C	A	0.025	0.004	4.60E-10	-0.0054	0.0112	0.6312	-0.0177	0.019	0.3521	0.0078	0.0285	0.7831
rs12796488	11	94131557	intron	GPR83	C	A	0.056	0.004	6.90E-46	0.0155	0.0112	0.1669	0.0115	0.0192	0.5485	0.0231	0.0278	0.4063
rs503542	11	118590743	intron	AP002954.4	G	A	0.018	0.003	5.80E-10	-0.0066	0.0083	0.4305	-0.0034	0.0143	0.8142	-0.0088	0.0207	0.6698
rs56196860	12	2908330	missense	FKBP4	A	C	0.321	0.009	1.00E-200	0.0353	0.0287	0.2199	0.0583	0.0489	0.2329	-0.0455	0.0721	0.5275
rs12810788	12	116196322	-	RP11-110L15.1	G	A	0.027	0.004	1.10E-11	0.0222	0.0108	0.03952	0.0176	0.0187	0.3462	0.0531	0.0262	0.04299
rs6486542	12	130952209	intron	RIMBP2	C	T	0.026	0.003	2.90E-18	0.0018	0.0091	0.8445	0.0167	0.0155	0.2814	0.0368	0.0229	0.1087
rs2038695	13	100559123	intergenic	CLYBL	C	A	0.023	0.003	2.60E-15	-0.001	0.0097	0.9185	0.0026	0.0164	0.872	-0.0121	0.0245	0.6195

rs10137488	14	35797122	intergenic	PSMA6	C	T	0.053	0.01	2.10E-08	0.0108	0.0241	0.6527	-0.0395	0.0413	0.3393	-0.0176	0.0611	0.773
rs1272131	14	60886150	intron	C14orf39	C	T	0.027	0.003	1.40E-17	-0.009	0.0082	0.271	0.0213	0.0141	0.1306	-0.0539	0.0204	0.008183
rs1812755	14	90007637	intron	FOXN3	T	C	0.034	0.004	5.10E-19	-0.0107	0.0113	0.3472	0.0048	0.0197	0.8069	-0.0139	0.0282	0.6209
rs1454836	15	47551054	intron	SEMA6D	T	A	0.017	0.003	3.40E-08	0.0183	0.0085	0.03156	0.0041	0.0145	0.7777	-0.0177	0.0212	0.4023
rs17703883	15	51530097	intron	CYP19A1	C	T	0.044	0.004	7.50E-40	0.0085	0.0093	0.3606	0.0216	0.0159	0.1764	0.0031	0.0229	0.8932
rs13835	15	89056040	3_prime_UTR	DET1	A	C	0.019	0.003	4.90E-10	-0.0034	0.0081	0.6718	0.0155	0.0139	0.2666	0.001	0.0201	0.9586
rs2764772	16	20060653	intron	GPR139	A	T	0.037	0.003	5.40E-33	0.0079	0.0089	0.3713	2.00E-04	0.0154	0.9883	0.0734	0.0221	0.000906
rs8061590	16	28895130	intron	ATP2A1	A	G	0.029	0.003	3.40E-21	-0.0022	0.0083	0.788	0.007	0.0144	0.6246	0.0162	0.0207	0.4335
rs62041532	16	73922719	intergenic	RPSAP56	G	T	0.023	0.004	1.60E-10	0.007	0.0111	0.5255	0.0114	0.0188	0.545	0.075	0.0275	0.006334
rs1799941	17	7533423	5_prime_UTR	SHBG	G	A	0.034	0.003	7.70E-23	0.0025	0.0092	0.7873	0.0079	0.0159	0.6203	-0.0141	0.0228	0.5369
rs58879558	17	44095467	intron	MAPT	C	T	0.023	0.004	2.40E-09	-0.0187	0.0097	0.05529	-0.0225	0.0168	0.1803	-0.0531	0.0237	0.02504
rs8076703	17	75612643	intergenic	RP11-13K12.6	C	T	0.024	0.003	1.00E-12	-0.0045	0.0095	0.6327	0.004	0.0161	0.8017	-0.029	0.0239	0.2238
rs2668776	18	44750365	intron	SKOR2	C	T	0.029	0.003	1.70E-22	0.0136	0.008	0.08917	0.0037	0.0137	0.7861	0.0284	0.0198	0.152
rs2327121	20	8878250	intron	PLCB1	C	G	0.018	0.003	1.30E-08	0.0148	0.0086	0.08369	0.0215	0.0146	0.1418	0.0385	0.0215	0.07379
rs7265992	20	33525407	intron	GSS	G	A	0.032	0.004	4.00E-16	-0.0083	0.011	0.4483	0.0148	0.0189	0.435	0.0085	0.0267	0.7498
rs11703376	22	49678713	intergenic	-	T	C	0.04	0.003	5.90E-32	0.0163	0.0095	0.08627	0.0148	0.0163	0.3632	0.0381	0.0236	0.1066

*Aggressive cancer defined as Gleason grade 8+, or prostate cancer death, or metastases or PSA>100 ng/mL. Early-onset defined as diagnosed ≤ 55 years.

Abbreviations: Chr=chromosome; PSA=prostate-specific antigen; SE=standard error; SNP=single nucleotide polymorphism UTR=untranslated region.

Supplementary Table 12: Association of total testosterone SNPs used in 2-sample Mendelian randomization analyses with prostate cancer

							Association parameters with total testosterone			Association parameters with overall prostate cancer			Association parameters with aggressive prostate cancer			Association parameters with early-onset prostate cancer		
SNP	Chr	Position	Consequence	Nearest gene	Effect allele	Other allele	Beta	SE	P-value	Beta	SE	P-value	Beta	SE	P-value	Beta	SE	P-value
rs36086195	1	16510894	intergenic	ARHGEF19-AS1	T	C	0.019	0.003	1.70E-09	-0.0071	0.0082	0.3885	-0.002	0.0142	0.8862	0.0167	0.0204	0.4143
rs114165349	1	27021913	intron	ARID1A	G	C	0.148	0.01	6.70E-51	0.0242	0.0277	0.3823	0.0925	0.0487	0.05735	-0.0756	0.0695	0.277
rs3768321	1	40035928	intron	PABPC4	G	T	0.031	0.004	5.40E-15	-0.0179	0.0106	0.09203	-0.0025	0.018	0.8903	-0.0345	0.026	0.185
rs9970140	1	61684288	intron	NFIA	G	A	0.04	0.006	6.80E-12	0.0157	0.0168	0.3495	-0.0021	0.0288	0.9428	-0.0299	0.0423	0.479
rs6676846	1	92942352	intron	GFI1	A	G	0.03	0.004	1.20E-15	0.0025	0.0098	0.7961	0.0027	0.0168	0.8708	-0.0226	0.0244	0.3556
rs12406721	1	107563243	intergenic	PRMT6	T	G	0.033	0.003	2.30E-28	0.0049	0.0088	0.5773	-1.00E-04	0.0152	0.9969	0.0078	0.0217	0.7185
rs267733	1	150958836	missense	ANXA9	A	G	0.023	0.004	1.80E-08	-0.0461	0.0109	2.20E-05	-0.0376	0.0186	0.04376	-0.0784	0.0268	0.00346
rs34702488	1	163256609	-	NUF2	A	T	0.026	0.004	1.70E-11	-0.0213	0.0101	0.03458	-0.0079	0.0172	0.6444	-0.0235	0.0254	0.3563
rs35737316	1	204161534	-	-	T	C	0.036	0.004	3.50E-27	0.0017	0.0093	0.8552	-0.0075	0.0161	0.6427	-3.00E-04	0.0231	0.9886
rs10864086	1	214318748	intron	RP11-53A1.3	C	A	0.022	0.003	5.30E-11	0.0048	0.0094	0.6047	0.0095	0.0162	0.5582	-0.0018	0.0235	0.9384
rs12470971	2	11725241	intron	GREB1	A	G	0.017	0.003	2.60E-08	0.0016	0.0089	0.8575	-0.0089	0.0154	0.5648	-0.0035	0.0223	0.8765
rs1260326	2	27730940	missense	GCKR	C	T	0.062	0.003	2.70E-91	-0.0222	0.0082	0.00657	-0.0191	0.0139	0.1713	-0.0224	0.0202	0.2688
rs6736913	2	42510018	missense	EML4	A	G	0.062	0.01	1.20E-08	-0.012	0.0346	0.7288	0.0332	0.0585	0.5698	-0.0553	0.087	0.5253
rs12614829	2	64893183	Non coding transcript exon	AC007365.4	T	C	0.03	0.004	6.10E-17	-0.0191	0.0106	0.07095	-0.0372	0.0182	0.04085	-0.0084	0.0262	0.7489
rs6750410	2	70417730	5_prime_UTR	C2orf42	A	G	0.042	0.006	5.20E-12	0.041	0.0159	0.01008	0.0534	0.0273	0.05058	0.0538	0.0402	0.1814
rs10192634	2	180500950	intron	ZNF385B	T	C	0.023	0.003	2.00E-11	0.0021	0.0089	0.8171	-0.0052	0.0153	0.7307	0.0169	0.022	0.443
rs2551641	2	208410267	intron	CREB1	C	T	0.022	0.004	7.20E-09	0.008	0.0102	0.4351	0.0164	0.0176	0.3519	0.0416	0.0255	0.102
rs2012736	2	234622379	missense	UGT1A5	C	A	0.048	0.006	5.10E-19	0.0097	0.014	0.4876	0.006	0.024	0.8018	-0.0053	0.0347	0.8793
rs7610366	3	28810588	intron	RBMS3	T	C	0.021	0.003	2.20E-11	0.0114	0.0091	0.2102	0.0056	0.0156	0.7221	0.0152	0.0228	0.5051
rs55869022	3	61656512	intron	PTPRG	C	G	0.027	0.005	2.00E-09	0.0094	0.0133	0.4787	-0.0027	0.023	0.9066	0.0061	0.0321	0.8482
rs34040779	3	107235109	intergenic	CD200R1L	T	C	0.034	0.006	5.30E-09	0.0794	0.0158	4.58E-07	0.0587	0.0271	0.03022	0.1415	0.0413	0.0006126
rs645040	3	135926622	upstream	RP11-463H24.1	G	T	0.043	0.004	1.50E-35	-0.0089	0.0097	0.361	-0.0061	0.0167	0.7162	-0.0194	0.0238	0.4156
rs61762319	3	154801978	missense	MME	G	A	0.061	0.009	1.40E-11	0.0219	0.0355	0.5377	-0.0224	0.0612	0.7146	-0.0134	0.089	0.8807
rs59194935	3	172147239	upstream	BZW1P1	A	G	0.02	0.003	3.70E-11	-0.0048	0.0081	0.558	-0.024	0.014	0.08666	-0.0246	0.0201	0.2217
rs13108218	4	3443931	intron	HGFAC	A	G	0.035	0.003	1.90E-29	0.0035	0.0087	0.6829	0.0164	0.015	0.2714	0.0141	0.0216	0.5132
rs7679843	4	22028079	intron	RP11-17E2.2	G	C	0.043	0.005	1.20E-17	0.0095	0.0143	0.5054	-0.0025	0.0248	0.9207	0.0182	0.0353	0.6055
rs7696472	4	69538180	upstream	UGT2B15	A	G	0.038	0.003	1.50E-39	-0.0027	0.0089	0.7607	0.0059	0.0155	0.7035	0.0033	0.0223	0.8816
rs1441911	4	77193545	intron	FAM47E	G	T	0.022	0.004	5.90E-09	0.0089	0.0099	0.3716	0.002	0.0171	0.909	0.0358	0.0247	0.1464
rs11735092	4	88226231	3_prime_UTR	HSD17B13	T	C	0.037	0.003	9.30E-35	0	0.0081	0.9955	0.013	0.0139	0.3512	-0.0063	0.02	0.7517
rs1154401	4	100009738	intron	ADH5	G	C	0.028	0.003	2.90E-18	-0.002	0.0085	0.8157	0.014	0.0144	0.332	0.0058	0.021	0.7839
rs11099675	4	148985104	intron	ARHGAP10	T	C	0.021	0.004	8.80E-10	-0.0133	0.0097	0.1717	0.0028	0.0167	0.8672	-2.00E-04	0.0243	0.9925
rs60701	5	10733776	intron	DAP	C	T	0.019	0.003	4.30E-08	-0.0078	0.0092	0.3979	-0.0207	0.0159	0.1927	-0.0049	0.0229	0.8322
rs7735249	5	53310139	intron	ARL15	C	G	0.028	0.005	1.80E-09	0.0022	0.0126	0.8589	-0.0202	0.0217	0.3519	-0.0124	0.0314	0.6918
rs40270	5	55804552	downstream	C5orf67	A	C	0.022	0.004	2.40E-09	-0.004	0.0097	0.6837	-0.0179	0.0167	0.2829	-0.0035	0.0244	0.8848
rs112530420	5	95871370	intron	CAST	C	T	0.026	0.004	5.30E-10	-0.0075	0.0121	0.5365	-0.0028	0.0208	0.8938	-0.0112	0.0303	0.712
rs329122	5	133864599	intron	JADE2	G	A	0.018	0.003	2.90E-09	-0.0288	0.008	0.0003155	-0.0366	0.0136	0.007372	-0.0386	0.0198	0.05192
rs6870458	5	137818916	intergenic	PRELID2	T	G	0.023	0.003	4.10E-13	-0.0177	0.0084	0.03505	-0.0236	0.0144	0.1006	-0.0409	0.0208	0.04932
rs1349359	5	165901446	intergenic	HMP19	A	G	0.017	0.003	2.10E-08	0.0115	0.0087	0.1835	0.0312	0.0148	0.03524	0.0181	0.0217	0.4034
rs9461224	6	25936402	intergenic	ZFP57	T	G	0.018	0.003	4.00E-10	-0.0069	0.0081	0.3985	0.0112	0.0139	0.4211	-0.0021	0.0201	0.9176
rs543504257	6	32571403	intergenic	TBC1D22B	A	C	0.024	0.003	1.30E-11	-0.0207	0.0252	0.4117	-0.0143	0.0427	0.7384	-0.0015	0.07	0.983
rs6939861	6	41703041	intron	TFEB	G	A	0.025	0.003	1.70E-12	-8.00E-04	0.0093	0.9288	0.0099	0.0162	0.5391	0.021	0.0235	0.3719
rs1933801	6	105365725	intron	LIN28B-AS1	T	C	0.038	0.003	7.80E-33	0.0089	0.0085	0.293	-0.0207	0.0145	0.1528	0.022	0.0209	0.2931
rs7773995	6	154382367	intron	OPRM1	C	T	0.024	0.004	1.20E-09	5.00E-04	0.0109	0.9649	0.0185	0.0188	0.3271	-0.0018	0.0273	0.9488

rs9986829	7	15019259	upstream	DGKB	A	G	0.041	0.003	5.20E-45	0.0011	0.0085	0.8949	0.0172	0.0145	0.2365	0.007	0.0211	0.7408
rs1708302	7	28198677	intron	JAZF1	T	C	0.018	0.003	2.60E-09	0.0134	0.0079	0.09142	0.0308	0.0136	0.02367	0.0287	0.0197	0.1445
rs10279715	7	40870935	intron	SUGCT	A	G	0.018	0.003	9.90E-10	0.0412	0.0082	4.87E-07	0.0459	0.014	0.001002	0.0431	0.0204	0.03434
rs1229498	7	81568750	intergenic	CACNA2D1	T	G	0.019	0.003	6.50E-09	-0.0057	0.0094	0.5481	-0.0054	0.0162	0.7381	0.0318	0.0235	0.1765
rs7015	7	97920623	3_prime_UTR	BRI3	G	A	0.056	0.004	7.60E-49	-0.0612	0.0102	1.96E-09	-0.0422	0.0175	0.01592	-0.0793	0.0251	0.001595
rs4841133	8	9183664	Non coding transcript exon	RP11-115J16.1	G	A	0.031	0.005	8.90E-10	-0.0055	0.0138	0.6893	-0.0032	0.0239	0.8949	0.0196	0.0353	0.5789
rs7835492	8	21089517	intergenic	RP11-24P4.1	C	T	0.025	0.004	1.10E-09	0.0196	0.0113	0.08248	0.0318	0.0194	0.1012	0.0502	0.0279	0.07181
rs4871844	8	22879734	3_prime_UTR	TNFRSF10B	C	T	0.017	0.003	3.10E-08	-0.0439	0.0085	2.52E-07	-0.0299	0.0146	0.04002	-0.0566	0.0212	0.007422
rs10958704	8	38328302	upstream	FGFR1	A	G	0.02	0.003	2.70E-11	0.0145	0.0083	0.07864	0.0229	0.0144	0.1112	0.0302	0.0207	0.1446
rs7844586	8	61782304	downstream	CHD7	C	T	0.02	0.004	1.10E-08	0.026	0.0096	0.006547	0.0334	0.0164	0.04173	0.0407	0.0235	0.08299
rs55867305	8	77884459	intergenic	hsa-mir-3149	G	A	0.033	0.003	1.80E-21	6.00E-04	0.0097	0.9498	-0.0073	0.0166	0.6608	0.0368	0.0248	0.1375
rs34955534	8	81710349	intron	ZNF704	G	A	0.042	0.005	4.10E-16	-0.003	0.0139	0.8295	-0.0087	0.0238	0.7158	-0.0427	0.0338	0.2069
rs7828742	8	116960729	downstream	LINC00536	A	G	0.017	0.003	2.50E-08	-0.0088	0.0085	0.3034	-0.0089	0.0146	0.5418	0.0114	0.0213	0.5928
rs2721195	8	145677011	intron	CYHR1	T	C	0.02	0.003	7.50E-11	0.0074	0.0084	0.3752	0.0091	0.0145	0.5297	0.0407	0.0211	0.05341
rs12336359	9	4129657	intron	GLIS3	C	G	0.019	0.003	1.70E-09	-0.006	0.0085	0.4826	-0.023	0.0146	0.1146	-0.0061	0.0212	0.774
rs112107457	9	19103774	upstream	HAUS6	T	C	0.029	0.005	1.00E-10	0.0305	0.0122	0.01278	2.00E-04	0.021	0.9916	0.0326	0.0301	0.2795
rs3808869	9	34622389	missense	ARID3C	C	A	0.017	0.003	5.40E-09	0.0277	0.008	0.0005217	0.0234	0.0137	0.08781	0.0385	0.0198	0.05154
rs199950405	9	83271419	intergenic	AL353707.1	C	G	0.019	0.004	4.20E-08	0.011	0.0095	0.2456	0.0088	0.0162	0.5881	0.0015	0.024	0.9488
rs10868080	9	86626769	intergenic	RMI1	T	A	0.036	0.003	1.20E-27	-0.022	0.009	0.01468	-0.0263	0.0155	0.09077	-0.0136	0.0224	0.545
rs2090409	9	108967088	intron	LINC01505	C	A	0.026	0.003	2.20E-16	0.0015	0.0084	0.8582	0.0051	0.0144	0.7206	-0.0264	0.0208	0.2053
rs10982192	9	117149417	intron	AKNA	T	C	0.024	0.004	6.30E-11	0.0257	0.0098	0.008972	0.0454	0.0168	0.006861	0.0212	0.0248	0.3921
rs13289095	9	131466489	intron	PKN3	G	T	0.021	0.004	4.00E-08	-0.0079	0.0125	0.5309	-0.0083	0.0217	0.701	0.0067	0.0313	0.8317
rs35182096	9	137268682	intron	RXRA	C	T	0.023	0.004	1.30E-10	0.0046	0.01	0.6462	0.037	0.0171	0.03074	0.0322	0.0252	0.2016
rs79717793	10	5262267	downstream	AKR1C4	G	A	0.049	0.004	9.10E-36	0.0163	0.011	0.1382	0.0262	0.0188	0.1647	0.0045	0.0271	0.8681
rs7912521	10	67262089	intergenic	LINC01515	C	T	0.049	0.003	2.60E-61	-0.001	0.0082	0.9003	0.0053	0.0141	0.7085	-0.0136	0.0203	0.5039
rs2862954	10	101912064	missense	ERLIN1	C	T	0.018	0.003	5.80E-09	-0.0079	0.0081	0.328	-0.0263	0.014	0.05925	-0.0316	0.0202	0.1173
rs7915430	10	121660465	intron	SEC23IP	T	G	0.025	0.004	2.30E-12	0.0026	0.0098	0.7913	-0.0024	0.0169	0.8853	-0.0176	0.0245	0.474
rs4757142	11	13325695	intron	ARNTL	G	A	0.018	0.003	6.40E-09	-0.0105	0.0084	0.2099	-0.0052	0.0144	0.7205	-0.0123	0.0209	0.5549
rs10832570	11	16249510	intron	SOX6	A	G	0.025	0.003	1.80E-16	-0.029	0.0083	0.0004454	-0.0237	0.0142	0.0957	-0.0836	0.0205	4.49E-05
rs1994721	11	29204531	intron	RP11-466I1.1	G	A	0.049	0.004	1.90E-32	0.0142	0.0112	0.2055	0.0098	0.0193	0.6132	0.0453	0.0281	0.1062
rs11607114	11	48151287	intron	PTPRJ	C	G	0.025	0.004	4.20E-10	-0.0114	0.0112	0.3111	-0.0231	0.0194	0.2336	-0.0226	0.0276	0.412
rs10750766	11	65473798	intergenic	KAT5	C	A	0.022	0.003	1.60E-11	0.0029	0.0089	0.7434	0.0032	0.0154	0.8348	-0.0246	0.0222	0.2661
rs631695	11	69283303	intron	AP000439.5	T	G	0.024	0.003	7.20E-17	0.0045	0.0082	0.585	0.0148	0.0141	0.2911	-0.014	0.0203	0.489
rs12796488	11	94131557	intron	GPR83	C	A	0.041	0.004	2.00E-26	0.0155	0.0112	0.1669	0.0115	0.0192	0.5485	0.0231	0.0278	0.4063
rs4754839	11	102157900	intergenic	RP11-864G5.1	A	G	0.018	0.003	1.10E-08	1.00E-04	0.0088	0.9932	0.0198	0.0153	0.1958	-0.0278	0.0227	0.2219
rs56196860	12	2908330	missense	FKBP4	A	C	0.301	0.009	1.00E-200	0.0353	0.0287	0.2199	0.0583	0.0489	0.2329	-0.0455	0.0721	0.5275
rs73079476	12	21343833	intron	SLCO1B1	A	C	0.054	0.004	4.20E-40	0.0026	0.0108	0.809	0.0096	0.0185	0.6025	0.0528	0.0272	0.05207
rs12320328	12	25408464	upstream	KRAS	A	G	0.043	0.005	2.30E-16	-0.0183	0.0154	0.2349	0.0076	0.0264	0.7732	-0.0333	0.039	0.3933
rs540730	12	57807114	intron	R3HDM2	T	C	0.03	0.004	3.70E-19	-0.0061	0.0098	0.5382	-0.0119	0.0168	0.4804	0.0385	0.0246	0.1174
rs2583948	12	66194613	intron	RPSAP52	A	G	0.035	0.005	3.00E-11	0.005	0.0136	0.7157	0.0168	0.0236	0.4765	0.0177	0.0334	0.5962
rs7314285	12	111522026	intron	CUX2	G	T	0.039	0.006	8.80E-12	0.0198	0.0173	0.2519	0.0324	0.03	0.2792	0.0723	0.0424	0.08787
rs3809272	12	111800258	3_prime_UTR	FAM109A	G	A	0.02	0.003	3.90E-10	-0.0239	0.009	0.007835	-0.0178	0.0156	0.254	-0.0415	0.0224	0.06417
rs2393775	12	121424574	intron	HNF1A	A	G	0.026	0.003	2.70E-17	-0.013	0.0082	0.1137	-0.0239	0.0142	0.09325	-0.0041	0.0206	0.8411
rs7997628	13	95217852	intergenic	TGDS	A	T	0.017	0.003	2.80E-08	0.0037	0.0084	0.659	-0.0187	0.0146	0.1996	-0.001	0.0211	0.9624
rs2038695	13	100559123	intergenic	CLYBL	C	A	0.021	0.003	1.10E-12	-0.001	0.0097	0.9185	0.0026	0.0164	0.872	-0.0121	0.0245	0.6195
rs3742223	13	112725196	downstream	SOX1	T	C	0.03	0.005	2.80E-10	0.0124	0.0144	0.3904	0.0177	0.0249	0.4782	-0.0191	0.0356	0.5916
rs11621792	14	24871926	intron	NYNRIN	C	T	0.018	0.003	6.70E-09	0	0.0086	0.997	-0.0107	0.0148	0.4683	-0.0132	0.0215	0.5388
rs2239222	14	73011885	intron	RGS6	G	A	0.021	0.003	1.80E-10	-0.0033	0.0087	0.7023	-0.0056	0.0147	0.7042	0.0321	0.0215	0.1351
rs72721770	14	74204686	intron	ELMSAN1	G	C	0.019	0.003	1.00E-09	-0.0059	0.0086	0.4938	-0.0351	0.0148	0.01762	0.0067	0.0216	0.7578
rs28929474	14	94844947	missense	SERPINA1	T	C	0.222	0.011	1.20E-95	-0.1338	0.0302	9.61E-06	0.0014	0.0494	0.978	-0.1834	0.0752	0.01467

rs17580	14	94847262	missense	SERPINA1	A	T	0.049	0.007	1.60E-12	-0.0093	0.0198	0.6388	0.0257	0.0339	0.4484	-0.006	0.0468	0.8978
rs45490496	14	105272678	downstream	ZBTB42	A	T	0.021	0.003	2.60E-11	-0.0169	0.0085	0.04757	-0.0278	0.0148	0.06029	0.0141	0.0213	0.5082
rs55707100	15	43820717	missense	MAP1A	C	T	0.111	0.01	1.80E-33	0.0406	0.0264	0.1244	0.0537	0.047	0.2526	0.0606	0.0659	0.3575
rs79391862	15	53739426	intergenic	WDR72	A	C	0.157	0.013	5.60E-38	0.1154	0.0378	0.002246	0.0586	0.065	0.3668	0.1262	0.1058	0.2329
rs7166920	15	96219503	intron	RP11-61O11.1	A	G	0.02	0.003	1.10E-10	0.0089	0.0081	0.2705	0.0041	0.014	0.7719	0.0333	0.0202	0.09918
rs56332871	15	96714816	Non coding transcript exon	RP11-327J17.2	A	C	0.047	0.003	1.70E-47	0.0208	0.0096	0.03118	-0.0029	0.0165	0.8623	0.0601	0.0241	0.01269
rs841194	16	4667690	-	MGRN1	G	A	0.027	0.004	6.70E-11	0.001	0.0113	0.9276	-0.0134	0.0192	0.4845	-0.0304	0.0282	0.281
rs2764772	16	20060653	intron	GPR139	A	T	0.032	0.003	2.50E-24	0.0079	0.0089	0.3713	2.00E-04	0.0154	0.9883	0.0734	0.0221	0.000906
rs1421085	16	53800954	intron	FTO	T	C	0.022	0.003	1.20E-11	0.0157	0.0082	0.05512	0.0013	0.0141	0.924	-0.0019	0.0204	0.9262
rs4525526	17	1650125	intron	SERPINF2	C	T	0.033	0.004	2.50E-20	-0.0229	0.01	0.02206	-0.0415	0.0172	0.016	-0.048	0.025	0.05454
rs1799941	17	7533423	5_prime_UTR	SHBG	A	G	0.197	0.003	1.00E-200	-0.0025	0.0092	0.7873	-0.0079	0.0159	0.6203	0.0141	0.0228	0.5369
rs2905801	17	29524974	intron	NF1	T	C	0.029	0.003	3.60E-19	0.0065	0.0088	0.4597	0.0046	0.0152	0.7642	0.0132	0.022	0.5481
rs62062271	17	44091988	intron	MAPT	C	T	0.031	0.004	5.40E-18	-0.0178	0.0097	0.06767	-0.0237	0.0168	0.1572	-0.053	0.0237	0.02519
rs28394864	17	47450775	intron	RP11-81K2.1	G	A	0.053	0.003	1.50E-72	0.005	0.0079	0.5319	0.0093	0.0136	0.4964	0.0114	0.0197	0.5613
rs2306216	17	73240559	intron	GGA3	A	G	0.027	0.004	9.20E-12	0.01	0.0108	0.3521	0.0068	0.0186	0.7122	-0.0492	0.0269	0.06728
rs7216664	17	73825664	-	-	G	A	0.019	0.003	2.40E-10	-0.0104	0.0092	0.2588	-0.0029	0.0158	0.8533	-0.0298	0.023	0.196
rs2668776	18	44750365	intron	SKOR2	C	T	0.023	0.003	6.80E-14	0.0136	0.008	0.08917	0.0037	0.0137	0.7861	0.0284	0.0198	0.152
rs1624295	19	2792034	intron	THOP1	A	G	0.033	0.003	7.20E-24	0.0187	0.0094	0.04669	-0.0041	0.016	0.7997	0.0158	0.0237	0.5048
rs8107967	19	7972615	intron	MAP2K7	G	A	0.019	0.003	1.70E-10	0.0205	0.0081	0.01173	4.00E-04	0.014	0.9749	0.0525	0.0204	0.01001
rs202200760	19	17346854	missense	NR2F6	C	G	0.12	0.009	8.60E-48	0.036	0.0197	0.06831	0.0409	0.0345	0.2358	0.0077	0.0494	0.8767
rs35824797	19	19456264	intron	MAU2	C	T	0.044	0.006	1.70E-15	-0.0216	0.0157	0.1691	0.013	0.0272	0.6327	0	0.0391	0.9991
rs34851490	19	46384554	downstream	IRF2BP1	G	A	0.048	0.005	1.80E-23	-0.0094	0.0129	0.4679	-0.0214	0.0223	0.3381	-0.0673	0.0318	0.03442
rs11671304	19	47564643	downstream	ZC3H4	C	T	0.016	0.003	4.40E-08	0.0187	0.0086	0.02925	0.0366	0.0148	0.01342	0.0287	0.0214	0.1787
rs6073431	20	43040569	intron	HNF4A	T	C	0.031	0.003	5.00E-24	-0.0029	0.0081	0.7198	6.00E-04	0.014	0.9657	-0.0084	0.0202	0.6768
rs1058319	20	62374389	3_prime_UTR	SLC2A4RG	T	C	0.024	0.005	1.50E-08	-0.1261	0.0124	1.92E-24	-0.1463	0.0216	1.37E-11	-0.1946	0.0311	3.93E-10
rs575146	22	24295074	downstream	GSTT2B	G	A	0.018	0.003	1.10E-08	-0.0132	0.0088	0.1313	0.0038	0.0152	0.8038	-0.0303	0.0219	0.1656
rs1033667	22	29130300	intron	CHEK2	T	C	0.023	0.003	9.30E-13	-0.0184	0.0089	0.03883	-0.0146	0.0153	0.3386	-0.0108	0.0219	0.6209
rs738409	22	44324727	missense	PNPLA3	G	C	0.051	0.004	4.30E-43	-0.022	0.01	0.02749	-0.0214	0.0171	0.2124	-0.0163	0.025	0.5143
rs11703376	22	49678713	intergenic	-	T	C	0.034	0.003	2.70E-23	0.0163	0.0095	0.08627	0.0148	0.0163	0.3632	0.0381	0.0236	0.1066

*Aggressive cancer defined as Gleason grade 8+, or prostate cancer death, or metastases or PSA>100 ng/mL. Early-onset defined as diagnosed ≤ 55 years.

Abbreviations: Chr=chromosome; PSA=prostate-specific antigen; SE=standard error; SNP=single nucleotide polymorphism UTR=untranslated region

Supplementary Table 13: Association of SHBG SNPs used in 2-sample Mendelian randomization analyses with prostate cancer

							Association parameters with IGF-I			Association parameters with overall prostate cancer			Association parameters with aggressive prostate cancer			Association parameters with early-onset prostate cancer		
SNP	Chr	Position	Consequence	Nearest gene	Effect allele	Other allele	Beta	SE	P-value	Beta	SE	P-value	Beta	SE	P-value	Beta	SE	P-value
rs36086195	1	16510894	intergenic	ARHGEF19-AS1	T	C	0.01674	0.00124	4.50E-44	-0.0071	0.0082	0.3885	-0.002	0.0142	0.8862	0.0167	0.0204	0.4143
rs114165349	1	27021913	intron	ARID1A	G	C	0.08711	0.00409	3.50E-111	0.0242	0.0277	0.3823	0.0925	0.0487	0.05735	-0.0756	0.0695	0.277
rs59708846	1	61687651	intron	NFIA	A	G	0.01949	0.00231	7.70E-18	0.0183	0.017	0.2809	0.0013	0.0291	0.9651	-0.0337	0.0427	0.4307
rs1730865	1	107605611	downstream	PRMT6	G	T	0.02605	0.00129	4.60E-98	0.0034	0.0086	0.6913	-0.0029	0.0149	0.8444	0.0029	0.0212	0.8922
rs72708162	1	149882811	intron	SV2A	T	G	0.01582	0.00227	9.60E-14	0.0457	0.0156	0.00341	0.0693	0.0268	0.009741	0.0745	0.0377	0.04797
rs2275560	1	164529666	intron	PBX1	G	A	0.00723	0.00157	3.00E-07	0.0191	0.0118	0.106	0.019	0.0195	0.3314	0.0031	0.0302	0.9192
rs12059956	1	171063262	intron	FMO3	G	A	0.00781	0.00124	4.30E-10	1.00E-04	0.0083	0.9916	-0.011	0.0143	0.4433	0.0265	0.0209	0.2054
rs78444298	1	184672098	missense	EDEM3	G	A	0.02587	0.00447	5.40E-11	-0.1077	0.0352	0.00225	-0.0838	0.0604	0.1652	-0.1252	0.089	0.1596
rs17583875	1	197924770	intergenic	LHX9	A	G	0.0242	0.00423	2.20E-09	-0.0122	0.0286	0.6697	-0.0331	0.0498	0.5064	0.0225	0.0709	0.7509
rs7540115	1	200265618	intron	LINC00862	C	A	0.00937	0.00159	1.10E-09	-2.00E-04	0.0109	0.9833	-0.0054	0.0188	0.7748	0.0098	0.0268	0.7134
rs10864086	1	214318748	intron	RP11-53A1.3	C	A	0.01521	0.00141	1.70E-28	0.0048	0.0094	0.6047	0.0095	0.0162	0.5582	-0.0018	0.0235	0.9384
rs2820441	1	219734960	downstream	RP11-95P13.2	C	A	0.00741	0.00131	2.70E-09	-0.0137	0.0087	0.1152	-0.0129	0.0149	0.3868	-0.0096	0.0216	0.6577
rs2247213	1	221055463	intron	HLX	G	A	0.01366	0.0013	5.70E-28	-0.0126	0.0087	0.1494	-0.0055	0.015	0.7161	-0.0198	0.0218	0.3626
rs1870927	1	226426337	-	LIN9	A	T	0.0065	0.00126	6.60E-07	0.0211	0.0086	0.01345	0.0085	0.0147	0.5634	0.0448	0.0214	0.03623
rs144647926	1	235467607	intron	ARID4B	A	G	0.01232	0.00217	1.40E-09	0.008	0.0156	0.6072	-0.0142	0.027	0.5998	0.0494	0.038	0.193
rs1260326	2	27730940	missense	GCKR	C	T	0.03808	0.00125	1.00E-200	-0.0222	0.0082	0.00657	-0.0191	0.0139	0.1713	-0.0224	0.0202	0.2688
rs138529890	2	32478354	intron	NLRC4	A	G	0.0253	0.00328	2.50E-14	-0.0134	0.0241	0.5792	-0.0548	0.0419	0.1902	-0.0478	0.0573	0.4046
rs6736913	2	42510018	missense	EML4	A	G	0.03291	0.00424	3.00E-15	-0.012	0.0346	0.7288	0.0332	0.0585	0.5698	-0.0553	0.087	0.5253
rs6750410	2	70417730	5 prime UTR	C2orf42	A	G	0.0182	0.00248	9.30E-14	0.041	0.0159	0.01008	0.0534	0.0273	0.05058	0.0538	0.0402	0.1814
rs3747647	2	112245586	intron	MIR4435-2HG	C	G	0.00827	0.00152	8.20E-09	0.0175	0.0129	0.1751	0.0123	0.0221	0.5785	0.0586	0.0327	0.07308
rs13389219	2	165528876	intron	COBLL1	T	C	0.01115	0.00125	8.60E-21	-0.0075	0.0081	0.3512	0.0028	0.0138	0.8367	-0.0481	0.02	0.0164
rs72948115	2	178167086	intron	NFE2L2	C	T	0.01228	0.00209	4.70E-09	0.0027	0.0144	0.8506	-0.0287	0.0246	0.2435	0.0262	0.0354	0.4599
rs8176526	2	188345322	intron	TFPI	C	T	0.00625	0.00138	2.00E-06	-0.0166	0.0088	0.06058	-0.0143	0.0151	0.3457	-0.0249	0.022	0.2564
rs4675682	2	208402750	intron	CREB1	T	C	0.00895	0.00123	3.10E-15	0.0036	0.008	0.6546	0.0151	0.0138	0.2735	0.0242	0.0199	0.2234
rs62182125	2	219274142	downstream	CTDSP1	G	A	0.00612	0.00124	5.50E-08	7.00E-04	0.0083	0.9325	0.008	0.0143	0.5756	-0.0119	0.0208	0.5661
rs12694450	2	220019638	intron	NHEJ1	T	C	0.00706	0.00136	3.80E-08	-0.0083	0.0085	0.333	-0.0208	0.0147	0.1587	-0.0182	0.0213	0.3943
rs2222018	2	227095220	intergenic	NEU2	C	A	0.01307	0.00128	1.40E-24	-0.0095	0.0084	0.2566	0	0.0145	0.9978	-0.0261	0.0211	0.2149
rs10153800	2	242179134	synonymous	HDLBP	A	G	0.00674	0.00145	5.90E-07	-0.0079	0.0094	0.4045	-0.0086	0.0162	0.5968	-0.0174	0.0234	0.4568
rs17036326	3	12389313	intron	PPARG	G	A	0.017	0.00188	1.20E-18	0.015	0.0119	0.2099	-0.0093	0.0206	0.6503	0.0035	0.0303	0.9086
rs6792725	3	24520283	intron	THRB	G	A	0.01205	0.00137	2.10E-21	-1.00E-04	0.0094	0.9902	-1.00E-04	0.0161	0.9953	0.0269	0.0237	0.2572
rs2564923	3	53103262	intron	RP11-894J14.5	A	G	0.0079	0.00124	7.80E-12	0.0065	0.008	0.4135	-0.0088	0.0137	0.5226	-0.0176	0.0199	0.3745
rs13315174	3	105406468	intron	CBLB	G	A	0.00736	0.0015	5.20E-08	-0.0159	0.0098	0.1047	-0.0435	0.0169	0.009792	-0.0052	0.0245	0.8307
rs687339	3	135932359	intergenic	KRT18P35	C	T	0.02755	0.00146	1.00E-87	-0.0097	0.0097	0.3156	-0.0085	0.0167	0.6093	-0.0205	0.0238	0.3887
rs7623513	3	142100428	intron	XRN1	C	A	0.00937	0.00185	3.70E-07	-0.0261	0.0117	0.02652	-0.0045	0.0202	0.8225	-0.0155	0.0294	0.5968
rs12696304	3	169481271	downstream	ACTRT3	G	C	0.00813	0.00139	1.70E-09	-0.0243	0.009	0.00685	-0.0327	0.0155	0.03484	-0.0319	0.0224	0.1534
rs79287178	3	172294500	intron	LINC02068	G	A	0.0397	0.00368	3.60E-30	-0.0129	0.0245	0.5985	-0.0248	0.0427	0.5605	0.02	0.0622	0.7482
rs7631981	3	185273510	upstream	LIPH	G	A	0.00692	0.00135	4.00E-08	1.00E-04	0.0096	0.9941	0.0021	0.0164	0.8969	-0.0099	0.0237	0.6766
rs35654957	4	1010077	intron	FGFRL1	T	C	0.00841	0.00128	2.60E-12	-0.0105	0.0084	0.21	-0.0082	0.0144	0.5714	-0.0332	0.0208	0.1104
rs13108218	4	3443931	intron	HGFAC	A	G	0.02299	0.00127	1.20E-79	0.0035	0.0087	0.6829	0.0164	0.015	0.2714	0.0141	0.0216	0.5132
rs11734408	4	23882519	5 prime UTR	PPARGC1A	G	A	0.00768	0.00135	3.30E-10	-0.0116	0.009	0.2013	-0.0116	0.0155	0.4551	-8.00E-04	0.0228	0.9703
rs1349852	4	69533217	Intron	UGT2B15	C	A	0.02037	0.00124	5.90E-67	-0.0031	0.0089	0.7258	0.004	0.0156	0.7979	0.0031	0.0225	0.8913
rs28507491	4	77197651	Intron	FAM47E	A	G	0.014	0.00127	5.70E-29	-0.0089	0.0084	0.2889	-0.0072	0.0146	0.6187	-0.0301	0.021	0.1509
rs7694379	4	88186509	Intron	RP11-529H2.1	G	A	0.02064	0.00124	4.00E-66	-0.0045	0.0081	0.5757	0.0081	0.014	0.5642	-0.0077	0.0201	0.7005
rs6831352	4	100063525	Intron	ADH4	T	C	0.01983	0.00134	1.00E-50	-0.0039	0.0087	0.6508	0.0061	0.0149	0.683	-0.0111	0.0217	0.6092
rs7655064	4	120106348	Intron	MYOZ2	T	C	0.00787	0.00185	6.50E-07	0.0157	0.0123	0.2027	0.0202	0.0212	0.3418	-0.0055	0.0312	0.8596
rs10027275	4	148981496	-	ARHGAP10	G	C	0.01255	0.00141	1.70E-21	-0.0101	0.0097	0.296	0.0087	0.0167	0.5997	-0.0021	0.0242	0.93
rs78890745	4	159834474	Intron	C4orf45	A	G	0.01365	0.00198	8.90E-12	-0.0045	0.0144	0.7552	-0.0216	0.0248	0.3846	-0.0542	0.0367	0.1404
rs11732763	4	171010101	Intron	AADAT	A	G	0.01249	0.002	3.00E-11	-0.0285	0.0146	0.05076	-0.0345	0.0253	0.172	-0.0246	0.036	0.4941
rs29681	5	190639	Upstream	LRRC14B	T	C	0.00862	0.0019	6.30E-06	-0.0046	0.0128	0.7223	-0.0175	0.0221	0.4286	-0.0139	0.0334	0.6784
rs10069690	5	1279790	Intron	TERT	C	T	0.00663	0.00141	8.70E-07	0.1332	0.0094	1.44E-45	0.1514	0.0166	8.83E-20	0.2083	0.0247	3.00E-17
rs7735249	5	53310139	Intron	ARL15	C	G	0.01751	0.00195	2.40E-21	0.0022	0.0126	0.8589	-0.0202	0.0217	0.3519	-0.0124	0.0314	0.6918

rs40270	5	55804552	downstream	C5orf67	A	C	0.01417	0.00147	1.30E-22	-0.004	0.0097	0.6837	-0.0179	0.0167	0.2829	-0.0035	0.0244	0.8848
rs11739158	5	72927292	Intron	ARHGEF28	T	C	0.00829	0.00125	2.10E-12	-0.0023	0.0083	0.7773	0.008	0.0142	0.576	0.0089	0.0207	0.6656
rs6595447	5	122750847	Intron	CEP120	T	C	0.00824	0.00157	2.90E-07	0.0113	0.0103	0.2761	0.0267	0.0177	0.1319	0.0195	0.0257	0.4483
rs329122	5	133864599	Intron	JADE2	G	A	0.00613	0.00125	4.80E-07	-0.0288	0.008	0.00032	-0.0366	0.0136	0.007372	-0.0386	0.0198	0.05192
rs11743810	5	137802404	Intron	EGR1	T	C	0.00894	0.00124	2.00E-12	-0.0189	0.0084	0.02476	-0.0205	0.0144	0.1569	-0.0473	0.0209	0.02376
rs2431752	5	162882702	Intron	NUDCD2	A	G	0.01016	0.002	7.50E-08	-0.0153	0.0125	0.222	0.0134	0.0215	0.5332	-0.0221	0.0317	0.4858
rs55646464	5	173324971	Intron	CPEB4	G	T	0.00673	0.00134	3.90E-07	0.005	0.0087	0.5628	0.0098	0.015	0.5152	-0.0074	0.0218	0.7359
rs9379084	6	7231843	missense	RREB1	G	A	0.01533	0.00199	1.00E-15	-0.0199	0.0131	0.129	-0.0121	0.0226	0.5933	-0.0469	0.0321	0.144
rs62394490	6	25934018	Upstream	SLC17A2	T	A	0.0109	0.00126	1.90E-20	-0.0056	0.0081	0.4889	0.0132	0.0139	0.3444	-0.0027	0.0202	0.8916
rs4714001	6	36638175	Upstream	LAP3P2	G	A	0.00539	0.00128	2.90E-06	0.0108	0.0082	0.1878	0.0125	0.014	0.375	0.0238	0.0203	0.241
rs6939861	6	41703041	Intron	TFEB	G	A	0.01128	0.00142	1.10E-15	-8.00E-04	0.0093	0.9288	0.0099	0.0162	0.5391	0.021	0.0235	0.3719
rs4715316	6	52628998	Upstream	GSTA2	T	C	0.00866	0.00129	2.00E-13	-0.003	0.0085	0.7278	-0.0168	0.0147	0.2533	0.0129	0.0213	0.5439
rs17185536	6	100620931	Upstream	RP3-344J20.1	T	C	0.00483	0.00144	0.00014	0.0061	0.0105	0.5604	0.0131	0.0178	0.4644	-0.0268	0.0262	0.3054
rs1890426	6	116338065	Intron	FRK	C	T	0.00749	0.00126	2.30E-09	-0.001	0.0081	0.8979	0.0093	0.0139	0.5051	-0.0038	0.0203	0.8529
rs6900473	6	130375810	Intron	L3MBTL3	A	G	0.0089	0.00133	7.40E-12	-0.0344	0.0087	7.82E-05	-0.0375	0.015	0.01256	-0.085	0.0218	9.38E-05
rs501470	6	160770918	Intron	SLC22A3	G	T	0.0152	0.00124	6.70E-40	-0.0814	0.0079	4.66E-25	-0.0678	0.0135	5.18E-07	-0.1262	0.0195	1.01E-10
rs62442919	7	1978384	Intron	MAD1L1	A	G	0.00702	0.00128	1.10E-09	-0.0299	0.0085	0.00045	-0.0585	0.0148	8.01E-05	-0.0417	0.0214	0.05137
rs2106727	7	17287998	intergenic	AC003075.4	G	A	0.00485	0.00128	2.30E-05	-0.0064	0.0084	0.4448	-0.0042	0.0144	0.7718	-0.0137	0.0207	0.5096
rs860262	7	28194397	Intron	JAZF1	A	C	0.00975	0.00123	2.20E-17	0.0107	0.0079	0.1753	0.0266	0.0136	0.04998	0.029	0.0196	0.1393
rs1799831	7	44199142	Intron	GCK	C	T	0.00878	0.00169	2.90E-07	-0.0045	0.0111	0.6884	0.006	0.0192	0.7524	-0.0512	0.0277	0.06496
rs73109480	7	44811221	downstream	ZMIZ2	T	C	0.01357	0.0024	2.80E-09	-0.0215	0.0168	0.2009	-0.0042	0.0292	0.8844	-0.0545	0.0413	0.1867
rs12536766	7	70158864	Intron	AUTS2	T	G	0.00528	0.00125	0.00014	-0.0062	0.0086	0.4755	-0.0068	0.0151	0.6513	0.0347	0.0216	0.1086
rs17145750	7	73026378	Intron	MLXIPL	T	C	0.01074	0.00167	9.70E-11	-0.0291	0.0122	0.01722	0.0048	0.0208	0.8179	-0.0166	0.0303	0.5825
rs1229492	7	81564122	intergenic	CACNA2D1	T	C	0.01147	0.0014	6.30E-18	-0.002	0.0095	0.8352	0.0019	0.0163	0.9055	0.0409	0.0238	0.08626
rs445	7	92408370	Intron	CDK6	C	T	0.01405	0.0021	6.60E-11	0.0022	0.0139	0.8745	0.0133	0.0242	0.5828	-0.0142	0.0344	0.6795
rs6950023	7	97915635	Intron	BRI3	G	T	0.03167	0.00158	9.90E-96	-0.0612	0.0102	1.95E-09	-0.0416	0.0175	0.01757	-0.0794	0.0251	0.00158
rs187437	7	116445091	intergenic	CAPZA2	G	A	0.00827	0.00124	9.60E-14	-0.0029	0.0081	0.7187	-0.011	0.014	0.4327	-0.0312	0.0203	0.1237
rs157935	7	130585553	Intron	AC058791.1	G	T	0.0121	0.00134	1.50E-22	-0.0085	0.0092	0.3554	0.0079	0.0158	0.6141	0.0188	0.0231	0.4144
rs3812275	7	135064882	Intron	CNOT4	C	A	0.00677	0.00125	3.30E-08	0.0255	0.0087	0.00329	0.0096	0.015	0.5224	0.0138	0.0217	0.5267
rs114949263	7	150498245	5 prime UTR	TMEM176B	C	T	0.0188	0.00196	4.20E-25	0.0265	0.0139	0.05776	0.07	0.0238	0.003249	0.051	0.0348	0.1434
rs4841133	8	9183664	Non coding transcript exon	RP11-115J16.1	G	A	0.02213	0.00214	1.40E-28	-0.0055	0.0138	0.6893	-0.0032	0.0239	0.8949	0.0196	0.0353	0.5789
rs876435	8	22873533	Intron	RHOBTB2	G	A	0.00757	0.00126	3.00E-11	-0.037	0.008	3.88E-06	-0.0283	0.0137	0.03902	-0.0431	0.0198	0.02982
rs12543287	8	42334511	Intron	SLC20A2	C	G	0.01061	0.00128	1.90E-18	0.0055	0.0092	0.5483	0.0074	0.0157	0.6372	0.0199	0.023	0.3873
rs10107182	8	59392737	Intergenic	CYP7A1	T	C	0.0124	0.0013	2.80E-22	-0.0013	0.0085	0.8814	-0.0161	0.0146	0.2722	-0.0212	0.0212	0.3172
rs75349541	8	71152803	Intron	NCOA2	C	T	0.00887	0.00182	2.60E-07	-0.0194	0.0119	0.1025	-0.0289	0.0206	0.1621	0.0043	0.0298	0.8849
rs11994858	8	81273210	Intergenic	RP11-775E10.1	G	A	0.01143	0.0013	1.20E-19	0.0034	0.0084	0.6823	0.0063	0.0144	0.6638	-0.0138	0.0209	0.5099
rs2721195	8	145677011	Intron	CYHR1	T	C	0.01129	0.00124	5.30E-21	0.0074	0.0084	0.3752	0.0091	0.0145	0.5297	0.0407	0.0211	0.05341
rs10116426	9	4145648	Intron	GLIS3	C	A	0.0089	0.00125	5.60E-13	-0.009	0.0084	0.285	-0.0249	0.0144	0.08442	-0.0093	0.0209	0.6558
rs820503	9	6667928	Intron	RP11-390F4.6	C	A	0.01121	0.00179	3.70E-11	-0.0121	0.0116	0.2982	-0.0263	0.02	0.189	-0.0487	0.0288	0.09068
rs35234337	9	35661243	3_prime_UTR	ARHGEF39	C	T	0.00761	0.00141	2.30E-08	0.0146	0.0094	0.1206	0.0069	0.0163	0.6716	0.034	0.0232	0.1439
rs10868080	9	86626769	Intergenic	RMI1	T	A	0.02133	0.00141	1.50E-57	-0.022	0.009	0.01468	-0.0263	0.0155	0.09077	-0.0136	0.0224	0.545
rs56237852	9	100343212	Intron	TMOD1	C	A	0.00748	0.00162	5.70E-06	0.0177	0.0107	0.09769	0.0153	0.0184	0.4056	0.0512	0.0266	0.05463
rs62580766	9	113034490	Intergenic	TXN	T	C	0.00961	0.0016	1.90E-10	0.0096	0.0111	0.3893	-0.0064	0.0192	0.7392	-0.0306	0.0276	0.2691
rs1570516	9	119053275	Intron	PAPPA	T	C	0.00594	0.00146	1.60E-05	0.0258	0.0102	0.01184	-0.0084	0.0174	0.6289	0.0332	0.0257	0.1971
rs9697210	9	131468740	Intron	PKN3	G	A	0.01874	0.00175	1.10E-29	-0.0077	0.0125	0.5385	-0.0082	0.0216	0.7065	0.007	0.0312	0.8234
rs79717793	10	5262267	Downstream	AKR1C4	G	A	0.02461	0.00169	2.60E-55	0.0163	0.011	0.1382	0.0262	0.0188	0.1647	0.0045	0.0271	0.8681
rs3781085	10	13370958	Intron	SEPHS1	T	G	0.00589	0.00124	1.00E-07	-0.0064	0.0087	0.4621	-0.0297	0.0148	0.04488	0.0083	0.0218	0.7034
rs3006593	10	31171626	Intron	ZNF438	C	G	0.00767	0.00126	7.90E-10	-5.00E-04	0.0082	0.9537	-0.0054	0.0142	0.7026	0.0257	0.0203	0.2067
rs34390319	10	63960611	Intron	RTKN2	C	T	0.01529	0.00205	1.40E-12	0.0022	0.0139	0.8725	-0.0105	0.0238	0.6606	-0.0045	0.0348	0.8975
rs10822153	10	65056813	Intron	JMJD1C	A	C	0.06432	0.00122	1.00E-200	0.0026	0.008	0.7476	-0.0163	0.0139	0.2409	-0.0135	0.0201	0.5007
rs1782652	10	81074125	3_prime_UTR	ZMIZ1	T	A	0.01274	0.00128	5.80E-27	-0.0154	0.0088	0.07928	-0.0175	0.0151	0.2487	-0.0264	0.0219	0.2274
rs2259305	10	93615903	Intron	TNKS2	G	A	0.01189	0.00122	2.90E-22	-0.0134	0.0082	0.1025	-0.0116	0.0141	0.4076	0.0169	0.0204	0.4091
rs856534	10	94810665	Intron	EXOC6	A	G	0.01016	0.00125	6.70E-19	-0.0053	0.0082	0.5209	-0.0176	0.0142	0.2128	-3.00E-04	0.0205	0.9878
rs7096937	10	113950418	Intron	GPAM	T	C	0.00947	0.00138	5.30E-14	0.014	0.009	0.1218	0.0106	0.0156	0.4989	-0.0032	0.0226	0.8875
rs1037169	11	13361005	Intron	ARNTL	T	C	0.01153	0.00133	3.70E-20	-0.0119	0.0086	0.1628	-0.0242	0.0148	0.1018	0.0065	0.0214	0.7616
rs62618693	11	32956492	Missense	QSER1	T	C	0.01879	0.00295	5.20E-11	0.0231	0.0209	0.268	0.0579	0.0358	0.1058	0.0105	0.0511	0.8373
rs12364060	11	45889775	Intron	CRY2	T	C	0.00788	0.00145	1.60E-07	0.0076	0.0096	0.4305	-0.0026	0.0168	0.8774	3.00E-04	0.0243	0.9897
rs12797706	11	65561369	5_prime_UTR	OVOL1	A	G	0.01208	0.00147	6.10E-17	0.0118	0.0095	0.2155	0.0173	0.0164	0.2933	-0.0031	0.0238	0.8957

rs631695	11	69283303	Intron	AP000439.5	T	G	0.01683	0.00125	8.70E-47	0.0045	0.0082	0.585	0.0148	0.0141	0.2911	-0.014	0.0203	0.489
rs10895277	11	102084940	Intron	YAP1	A	G	0.00962	0.0013	8.90E-15	-0.0044	0.0089	0.6236	0.0054	0.0154	0.7284	-3.00E-04	0.0231	0.989
rs665731	11	114032484	Intron	ZBTB16	T	C	0.00652	0.00158	1.20E-05	0.0141	0.012	0.2397	0.0126	0.0204	0.5376	0.0152	0.0301	0.6127
rs2156805	11	122610568	Intron	UBASH3B	G	A	0.00538	0.00124	1.40E-06	-0.013	0.008	0.104	-0.0153	0.0137	0.263	-0.0121	0.0198	0.541
rs56196860	12	2908330	Missense	FKBP4	A	C	0.02193	0.00352	6.60E-11	0.0353	0.0287	0.2199	0.0583	0.0489	0.2329	-0.0455	0.0721	0.5275
rs76895963	12	4384844	Intron	CCND2	G	T	0.05541	0.00474	2.40E-33	0.0383	0.0317	0.2271	0.0422	0.0551	0.4439	0.0013	0.0783	0.987
rs1871395	12	21352315	Intron	SLCO1B1	A	G	0.0325	0.00171	4.60E-88	0.0024	0.0105	0.8163	0.0027	0.018	0.8801	0.0536	0.0268	0.04564
rs75130744	12	25410741	Intergenic	KRAS	G	C	0.0296	0.00239	3.10E-39	-0.0018	0.017	0.9182	0.0307	0.0292	0.2939	-0.0288	0.0426	0.4991
rs12818938	12	53783182	-	-	T	G	0.0095	0.00167	1.00E-08	-0.0157	0.0109	0.1517	-0.0246	0.0186	0.1869	-0.0179	0.0272	0.5103
rs540730	12	57807114	Intron	R3HDM2	T	C	0.01772	0.00143	4.90E-39	-0.0061	0.0098	0.5382	-0.0119	0.0168	0.4804	0.0385	0.0246	0.1174
rs61929307	12	69997422	Downstream	CCT2	G	T	0.00667	0.0013	1.30E-07	-0.0131	0.0083	0.1163	-0.0137	0.0143	0.3392	-0.0409	0.0208	0.04888
rs11111274	12	102838128	Intron	IGF1	G	A	0.00731	0.00139	4.60E-08	0.0238	0.0089	0.00768	0.0273	0.0153	0.07392	0.0579	0.0219	0.008388
rs7314285	12	111522026	Intron	CUX2	G	T	0.03012	0.00244	1.50E-41	0.0198	0.0173	0.2519	0.0324	0.03	0.2792	0.0723	0.0424	0.08787
rs9738226	12	121423659	Intron	HNF1A	G	A	0.01941	0.00127	5.20E-54	-0.0134	0.0082	0.1045	-0.0242	0.0142	0.08926	-0.0033	0.0206	0.8724
rs41284816	13	50655989	Upstream	DLEU1	T	G	0.01965	0.00462	1.90E-06	-0.0257	0.0265	0.3319	0.0109	0.0449	0.8085	-0.0646	0.0683	0.3442
rs116338429	13	114767040	Intron	RASA3	T	C	0.00986	0.00173	1.30E-09	-0.0097	0.0117	0.4068	0.0027	0.0202	0.8934	-0.0311	0.029	0.2841
rs11621792	14	24871926	Intron	NYNRIN	C	T	0.01275	0.00124	1.40E-25	0	0.0086	0.997	-0.0107	0.0148	0.4683	-0.0132	0.0215	0.5388
rs72683923	14	50735947	Synonymous	L2HGDH	C	T	0.03398	0.00444	3.00E-16	0.0362	0.0351	0.3017	-0.0761	0.0619	0.2188	-0.1214	0.0937	0.195
rs2239222	14	73011885	Intron	RGS6	G	A	0.01106	0.0013	5.50E-17	-0.0033	0.0087	0.7023	-0.0056	0.0147	0.7042	0.0321	0.0215	0.1351
rs13379043	14	74250126	Intron	ELMSAN1	C	T	0.00829	0.0014	2.20E-10	-8.00E-04	0.0095	0.9305	-0.0061	0.0163	0.7093	0.0635	0.0231	0.005971
rs28929474	14	94844947	Missense	SERPINA1	T	C	0.1383	0.00442	1.00E-200	-0.1338	0.0302	9.61E-06	0.0014	0.0494	0.978	-0.1834	0.0752	0.01467
rs3742366	14	104198351	Intron	ZFYVE21	C	T	0.00918	0.0013	3.70E-14	0.0228	0.0084	0.00656	0.01	0.0143	0.4844	0.0335	0.0209	0.1081
rs28562483	15	31660799	Intron	KLF13	G	T	0.01058	0.00186	4.90E-08	-0.0233	0.0129	0.07131	-0.0238	0.022	0.2806	-0.0523	0.0324	0.1069
rs139974673	15	44027885	Intron	CATSPER2P1	T	C	0.07389	0.00394	3.00E-84	0.0346	0.0268	0.1957	0.0643	0.0477	0.1775	0.0501	0.0672	0.4558
rs149624078	15	53728710	Intergenic	WDR72	C	T	0.09758	0.00544	5.40E-81	0.1139	0.0377	0.00254	0.0625	0.065	0.3358	0.1255	0.1057	0.2349
rs56187480	15	63789479	Downstream	AC007950.2	G	A	0.01266	0.0013	1.30E-25	-0.0024	0.0085	0.7772	-0.0203	0.0146	0.1641	0.0116	0.0212	0.5849
rs8038465	15	73978337	Intron	CD276	T	C	0.00618	0.00125	5.20E-08	0.009	0.0084	0.2854	0.0158	0.0144	0.272	0.0068	0.0208	0.7455
rs72753908	15	83334856	Intron	AP3B2	C	T	0.01085	0.00236	1.80E-06	-1.00E-04	0.0159	0.9972	0.0206	0.0274	0.4527	-0.0541	0.0386	0.1617
rs11856926	15	96223649	Intron	RP11-61O11.1	G	A	0.01159	0.00125	1.90E-22	0.0056	0.0081	0.4902	-8.00E-04	0.0141	0.9567	0.0267	0.0204	0.1898
rs56332871	15	96714816	Non coding transcript exon	RP11-327J17.2	A	C	0.02983	0.00139	4.20E-111	0.0208	0.0096	0.03118	-0.0029	0.0165	0.8623	0.0601	0.0241	0.01269
rs36108764	16	4624130	Intron	C16orf96	G	A	0.00953	0.00156	1.80E-10	-0.0065	0.0111	0.5593	-0.0191	0.0188	0.3099	-0.0394	0.0278	0.1566
rs12928099	16	15150505	Intron	PDXDC1	A	C	0.00913	0.00136	1.20E-11	0.0224	0.0093	0.01528	0.0344	0.0159	0.03016	0.0405	0.023	0.07784
rs2288004	16	31054040	Downstream	STX4	G	C	0.00701	0.00127	1.10E-08	-0.0123	0.0081	0.1293	-0.0145	0.0139	0.2957	-0.0147	0.0202	0.4679
rs28650012	16	80497341	Intron	RP11-525K10.3	G	C	0.00712	0.00138	2.50E-09	0.013	0.0091	0.1523	-0.0084	0.0157	0.5923	0.0046	0.0226	0.8396
rs1799941	17	7533423	5_prime_UTR	SHBG	A	G	0.12077	0.00136	1.00E-200	-0.0025	0.0092	0.7873	-0.0079	0.0159	0.6203	0.0141	0.0228	0.5369
rs12950562	17	17995166	Intron	DRG2	T	C	0.01102	0.00123	7.40E-21	-0.025	0.0081	0.0019	-0.0158	0.0138	0.2508	-0.0787	0.02	8.33E-05
rs2905801	17	29524974	Intron	NF1	T	C	0.01401	0.00131	3.80E-28	0.0065	0.0088	0.4597	0.0046	0.0152	0.7642	0.0132	0.022	0.5481
rs11655704	17	47448172	Non coding transcript exon	RP11-81K2.1	C	T	0.03116	0.00128	4.90E-136	0.0445	0.0086	2.44E-07	0.041	0.0148	0.005607	0.0431	0.0213	0.04279
rs1801689	17	64210580	Missense	APOH	A	C	0.03343	0.00351	1.80E-21	0.0257	0.0243	0.2919	0.053	0.0436	0.2237	0.1096	0.0604	0.06985
rs7210574	17	73824121	Synonymous	UNC13D	C	T	0.00977	0.00127	8.10E-16	-0.0069	0.0092	0.4502	0.0052	0.0157	0.7434	-0.0277	0.0229	0.2268
rs36013981	17	79493307	Upstream	FSCN2	A	G	0.00674	0.00122	3.80E-10	0.0137	0.0083	0.1012	0.0254	0.0144	0.07702	0.0039	0.0207	0.8512
rs55855238	18	55089715	Upstream	RP11-714M23.2	C	T	0.00858	0.0013	3.80E-13	-0.0086	0.0088	0.3297	-0.032	0.0151	0.03448	-0.0046	0.0222	0.8353
rs1788641	18	71949629	intron	CYB5A	A	G	0.00715	0.00134	2.00E-07	0.0087	0.0088	0.3224	0.0271	0.0152	0.07526	0.0056	0.0218	0.7985
rs1640267	19	2789337	intron	THOP1	C	T	0.01729	0.00136	5.90E-41	0.021	0.0098	0.03127	-5.00E-04	0.0166	0.9743	0.0193	0.0246	0.4311
rs60018147	19	3375572	intron	NFIC	G	A	0.01315	0.00198	1.00E-12	-0.0063	0.0165	0.7007	-0.0461	0.0285	0.1055	0.0215	0.0417	0.6068
rs202200760	19	17346854	missense	NR2F6	C	G	0.0711	0.00349	3.70E-98	0.036	0.0197	0.06831	0.0409	0.0345	0.2358	0.0077	0.0494	0.8767
rs35824797	19	19456264	intron	MAU2	C	T	0.01565	0.00229	1.10E-14	-0.0216	0.0157	0.1691	0.013	0.0272	0.6327	0	0.0391	0.9991
rs45512696	19	35550878	synonymous	HPN	T	C	0.02057	0.00162	1.10E-41	-0.0012	0.012	0.9237	0.0302	0.0203	0.1367	-0.0312	0.03	0.2992
rs11666245	19	38229926	missense	ZNF573	G	A	0.01633	0.0029	2.20E-09	0.0027	0.0192	0.8899	-0.0547	0.0329	0.09653	-0.0791	0.047	0.09215
rs34255979	19	46384830	downstream	IRF2BP1	T	C	0.02725	0.00191	4.00E-51	-0.0068	0.0127	0.5894	-0.0219	0.022	0.318	-0.0569	0.0312	0.068
rs111981233	19	50016479	5_prime_UTR	FCGRT	G	T	0.02616	0.00228	8.20E-33	0.0391	0.0154	0.01129	-1.00E-04	0.0267	0.9961	0.0209	0.0382	0.5841
rs13042148	20	32298286	intron	PXMP4	C	T	0.01245	0.00171	1.40E-14	0.0394	0.0113	0.00047	0.0108	0.0195	0.5788	0.0974	0.0283	0.000584
rs4812336	20	37547374	3_prime_UTR	PPP1R16B	A	G	0.00772	0.00134	9.20E-09	-0.0062	0.009	0.4914	0.0038	0.0155	0.8039	-0.0081	0.0224	0.7159
rs3746575	20	43058096	intron	HNF4A	G	C	0.01749	0.00128	2.20E-43	-0.0057	0.0084	0.4985	-0.0089	0.0144	0.5393	0.0046	0.0208	0.8258

rs78319058	20	49020571	intergenic	RP13-379L11.3	T	C	0.02487	0.0042	4.20E-10	0.0434	0.0296	0.1436	0.0856	0.0515	0.09628	0.1573	0.0728	0.03062
rs1058319	20	62374389	3_prime_UTR	SLC2A4RG	T	C	0.00923	0.00185	1.60E-07	-0.1261	0.0124	1.92E-24	-0.1463	0.0216	1.37E-11	-0.1946	0.0311	3.93E-10
rs2234694	21	33038865	intron	SOD1	A	C	0.013	0.00301	6.70E-07	0.026	0.0194	0.18	0.0243	0.0333	0.4655	-0.0331	0.0503	0.5107
rs6005840	22	29101357	intron	CHEK2	A	G	0.01687	0.00132	4.00E-39	-0.0085	0.0087	0.3287	-0.0059	0.0149	0.6945	0.0102	0.0213	0.6317
rs738409	22	44324727	missense	PNPLA3	G	C	0.03096	0.0015	1.80E-96	-0.022	0.01	0.02749	-0.0214	0.0171	0.2124	-0.0163	0.025	0.5143

*Aggressive cancer defined as Gleason grade 8+, or prostate cancer death, or metastases or PSA>100 ng/mL. Early-onset defined as diagnosed ≤55 years.

Abbreviations: Chr=chromosome; SE=standard error; SHBG=sex hormone binding globulin; SNP=single nucleotide polymorphism UTR=untranslated region.

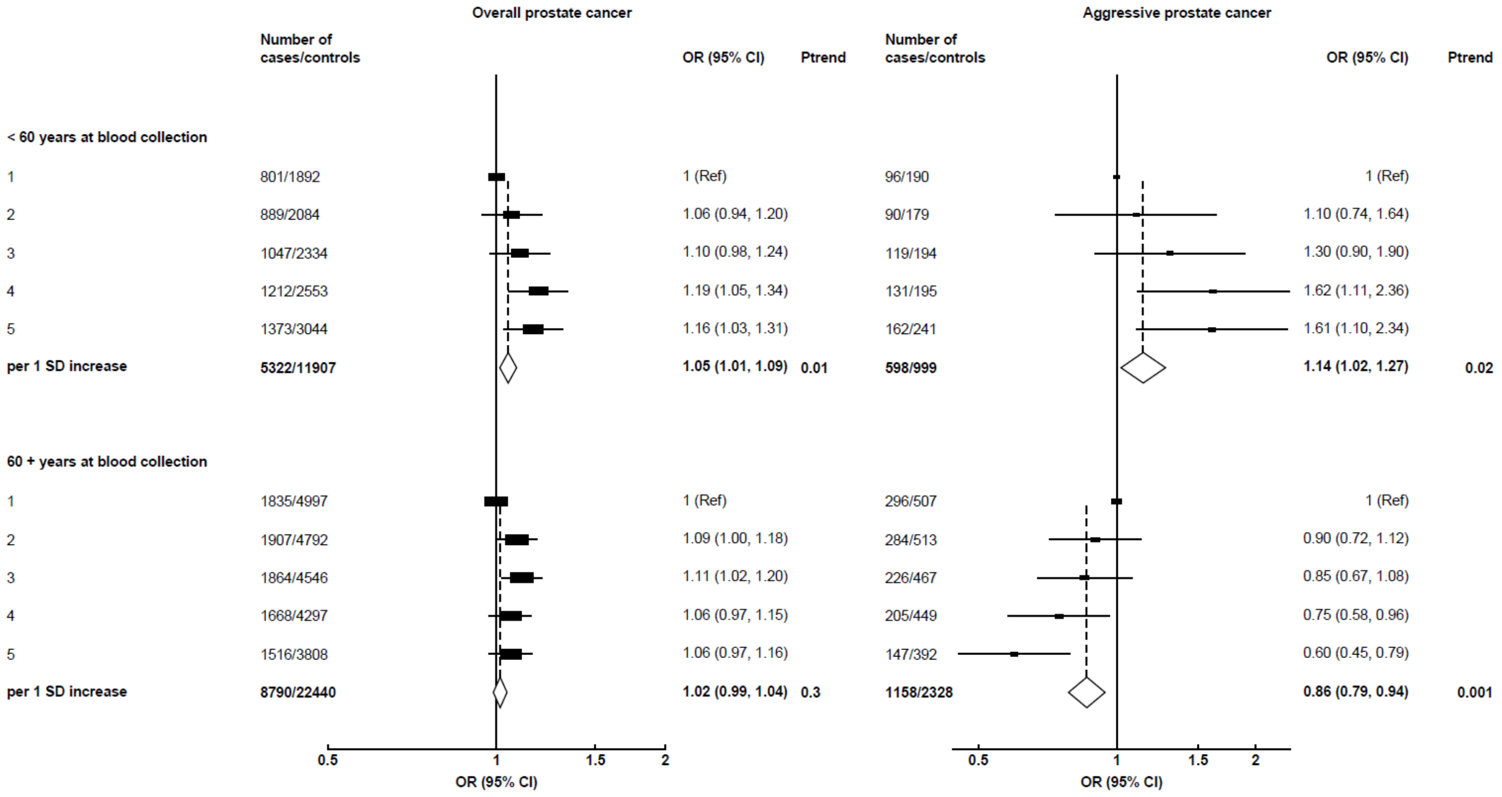

Supplementary Figure 1: Risks of overall and aggressive* prostate cancer in by study-specific fifths of free testosterone concentrations and per 1 SD increment, stratified by age at blood collection
Blood-based estimates are from logistic regression conditioned on the matching variables and adjusted for age, BMI, height, alcohol intake, smoking status, marital status, education status, racial/ethnic group, and diabetes status. The position of each square indicates the magnitude of the OR, and the area of the square is proportional to the inverse of the variance of the logarithm of the OR. The length of the horizontal line through the square indicates the 95% confidence interval.
*Aggressive cancer defined as Gleason grade 8+, or prostate cancer death, or metastases or PSA >100 ng/mL.
Abbreviations: BMI=body mass index; CI=confidence interval; OR=odds ratio; PSA=prostate-specific antigen; SD=standard deviation.

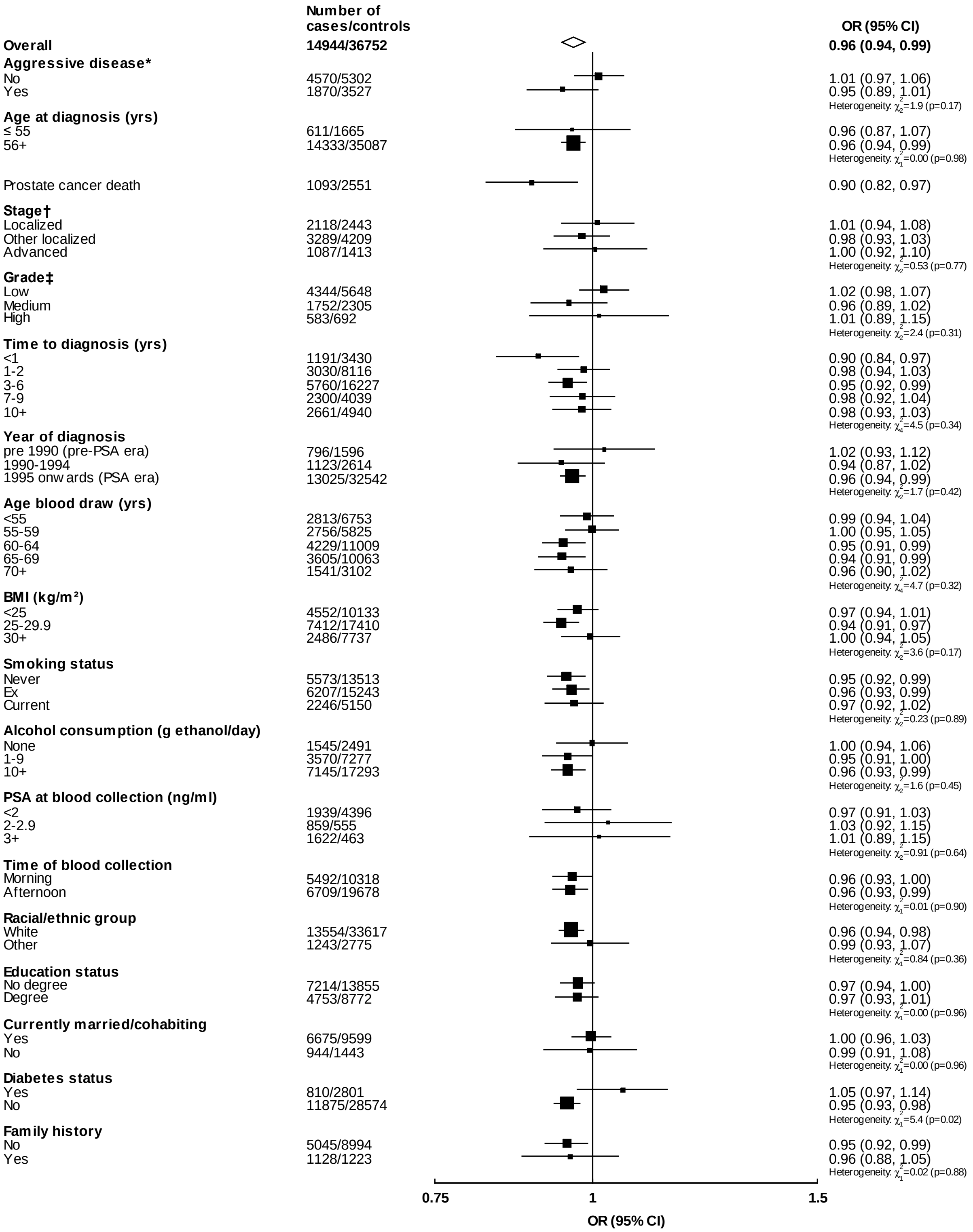

Supplementary Figure 2: Odds ratio (95% CIs) for overall prostate cancer per study-specific 1 SD increment of total testosterone concentration by subgroup

Estimates are from logistic regression conditioned on the matching variables and adjusted for age, BMI, height, alcohol intake, smoking status, marital status, education status, racial/ethnic group, and diabetes status. The position of each square indicates the magnitude of the OR, and the area of the square is proportional to the inverse of the variance of the logarithm of the OR. The length of the horizontal line through the square indicates the 95% confidence interval. Tests for heterogeneity for case-defined factors were obtained by fitting separate models for each subgroup and assuming independence of the ORs using a method analogous to a meta-analysis. Tests for heterogeneity for non–case-defined factors were assessed with a $\chi 2$ test of interaction between subgroup and the binary variable

*Aggressive cancer defined as Gleason grade 8+, or prostate cancer death, or metastases or PSA >100 ng/mL
†Localized defined as TNM stage <T2 with no reported lymph node involvement or metastases or stage I; other localized stage if TNM stage T2 with no reported lymph node involvement or metastases, stage II, or equivalent; advanced stage if they were TNM stage T3 or T4 and/or N1+ and/or M1, stage III–IV, or equivalent.
‡ Low grade defined as Gleason score was <7 or equivalent (i.e. extent of differentiation good, moderate); medium grade if Gleason score was 7 (i.e. poorly differentiated); high grade if the Gleason score was ≥8 or equivalent (i.e. undifferentiated).
Abbreviations: BMI=body mass index; CI=confidence interval; OR=odds ratio; PSA=prostate-specific antigen; SD=standard deviation; TNM= tumour, node, metastasis

	Number of cases/controls	OR (95% CI)
**Aggressive disease***	**1870/3527**	**0.95 (0.89, 1.01)**
**Age at diagnosis (yrs)**		
≤ 55	58/108	2.40 (1.28, 4.52)
56 +	1812/3419	0.94 (0.88, 1.00)
		Heterogeneity: χ^2_2=8.4 (p=0.004)
Protate cancer death	1093/2551	0.90 (0.82, 0.97)
**Stage†**		
Localized	539/864	0.98 (0.86, 1.11)
Advanced	682/943	1.05 (0.93, 1.18)
		Heterogeneity: χ^2_2=0.63 (p=0.43)
**Grade‡**		
Low	483/860	0.97 (0.85, 1.11)
High	583/692	1.01 (0.89, 1.15)
		Heterogeneity: χ^2_2=0.18 (p=0.67)
**Time to diagnosis (yrs)**		
<1	111/167	0.86 (0.63, 1.16)
1-4	649/1317	0.93 (0.83, 1.04)
5 +	1110/2043	0.96 (0.88, 1.04)
		Heterogeneity: χ^2_2=0.62 (p=0.74)
**Year of diagnosis**		
pre 1990 (pre-PSA era)	223/351	1.08 (0.88, 1.32)
1990-1994	183/309	0.94 (0.75, 1.19)
1995 onwards (PSA era)	1464/2867	0.93 (0.87, 1.00)
		Heterogeneity: χ^2_2=1.7 (p=0.42)
**Age blood draw (yrs)**		
<60	624/1034	1.09 (0.98, 1.22)
60-69	960/2006	0.89 (0.82, 0.98)
70+	286/487	0.84 (0.72, 0.98)
		Heterogeneity: χ^2_2=11 (p=0.004)
**BMI (kg/m²)**		
<25	574/1126	0.89 (0.80, 0.99)
25-29.9	918/1694	0.95 (0.87, 1.04)
30+	330/629	1.01 (0.85, 1.20)
		Heterogeneity: χ^2_2=1.7 (p=0.43)
**Smoking status**		
Never	595/1227	0.92 (0.83, 1.03)
Ex	810/1506	0.92 (0.83, 1.01)
Current	403/645	0.95 (0.84, 1.09)
		Heterogeneity: χ^2_2=0.21 (p=0.90)
**Alcohol consumption (g ethanol/day)**		
None	248/429	0.90 (0.76, 1.06)
1-9	474/801	0.93 (0.82, 1.06)
10+	875/1652	0.96 (0.88, 1.05)
		Heterogeneity: χ^2_2=0.58 (p=0.75)
**PSA at blood collection (ng/ml)**		
<2	269/961	1.06 (0.90, 1.24)
2-2.9	118/138	0.83 (0.61, 1.13)
3+	379/153	0.91 (0.70, 1.17)
		Heterogeneity: χ^2_2=2.4 (p=0.3)
**Time of blood collection**		
Morning	872/1414	0.97 (0.88, 1.06)
Afternoon	658/1521	0.91 (0.82, 1.01)
		Heterogeneity: χ^2_1=0.8 (p=0.37)
**Racial/ethnic group**		
White	1669/3141	0.95 (0.89, 1.02)
Other	163/282	0.82 (0.66, 1.01)
		Heterogeneity: χ^2_1=1.7 (p=0.19)
**Education status**		
No degree	1036/1599	0.93 (0.86, 1.02)
Degree	487/855	0.95 (0.83, 1.09)
		Heterogeneity: χ^2_1=0.07 (p=0.80)
**Currently married/cohabiting**		
Yes	1275/2303	0.93 (0.86, 1.01)
No	182/236	0.99 (0.81, 1.20)
		Heterogeneity: χ^2_1=0.25 (p=0.62)
**Diabetes status**		
Yes	121/240	0.92 (0.72, 1.19)
No	1474/2839	0.95 (0.88, 1.02)
		Heterogeneity: χ^2_1=0.03 (p=0.86)

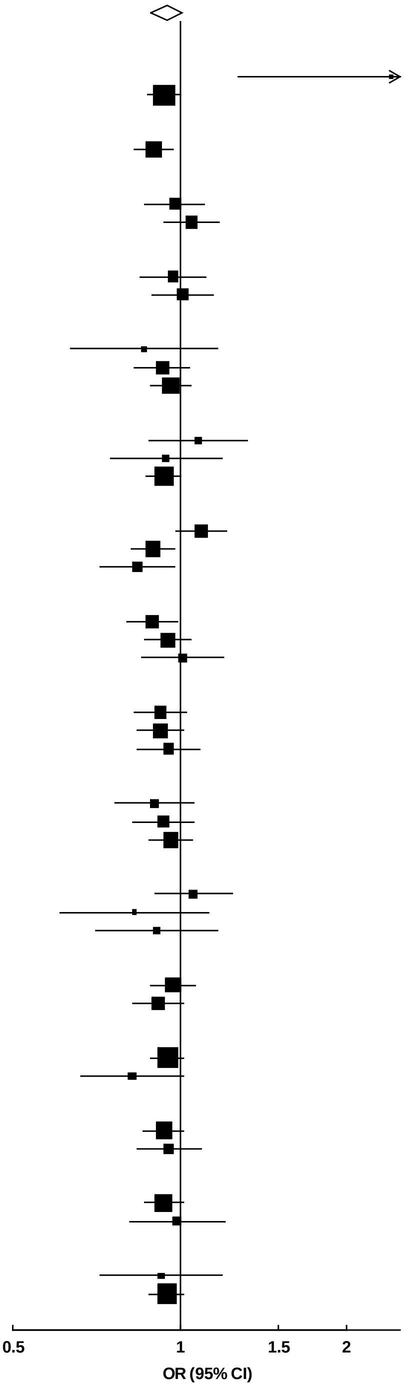

Supplementary Figure 3: Odds ratio (95% CIs) for aggressive* prostate cancer per study-specific 1 SD increment of total testosterone concentration by subgroup

Estimates are from logistic regression conditioned on the matching variables and adjusted for age, BMI, height, alcohol intake, smoking status, marital status, education status, racial/ethnic group, and diabetes status. The position of each square indicates the magnitude of the OR, and the area of the square is proportional to the inverse of the variance of the logarithm of the OR. The length of the horizontal line through the square indicates the 95% confidence interval. Tests for heterogeneity for case-defined factors were obtained by fitting separate models for each subgroup and assuming independence of the ORs using a method analogous to a meta-analysis. Tests for heterogeneity for non–case-defined factors were assessed with a $\chi 2$ test of interaction between subgroup and the binary variable

*Aggressive cancer defined as Gleason grade 8+, or prostate cancer death, or metastases or PSA >100 ng/mL
†Localized defined as TNM stage <T2 with no reported lymph node involvement or metastases or stage I, or TNM stage T2 with no reported lymph node involvement or metastases, stage II, or equivalent; advanced stage if they were TNM stage T3 or T4 and/or N1+ and/or M1, stage III–IV, or equivalent. Men with localized aggressive disease will have had high-grade prostate cancer, PSA > 100 ng/mL or died from prostate cancer.
‡ Low grade defined as Gleason score was <8 or equivalent (i.e., extent of differentiation good, moderate, poor); high grade if the Gleason score was ≥8 or equivalent (i.e., undifferentiated). Men with low-grade aggressive disease will have had advanced prostate cancer, PSA > 100 ng/mL or died from prostate cancer.
Abbreviations: BMI=body mass index; CI=confidence interval; OR=odds ratio; PSA=prostate-specific antigen; SD=standard deviation; TNM= tumour, node, metastasis.

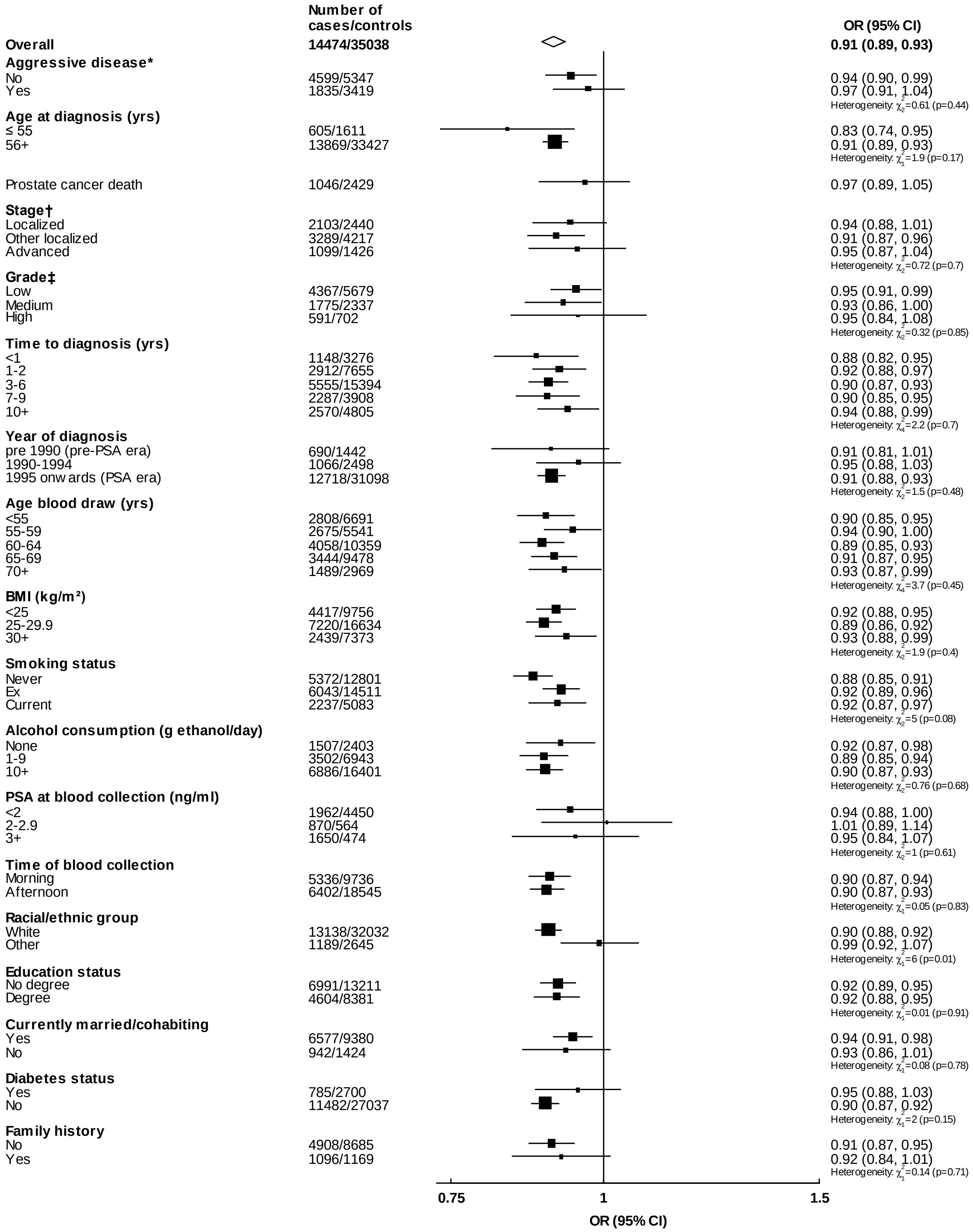

Supplementary Figure 4: Odds ratio (95% CIs) for overall prostate cancer per study-specific 1 SD increment of SHBG concentration by subgroup

Estimates are from logistic regression conditioned on the matching variables and adjusted for age, BMI, height, alcohol intake, smoking status, marital status, education status, racial/ethnic group, and diabetes status. The position of each square indicates the magnitude of the OR, and the area of the square is proportional to the inverse of the variance of the logarithm of the OR. The length of the horizontal line through the square indicates the 95% confidence interval. Tests for heterogeneity for case-defined factors were obtained by fitting separate models for each subgroup and assuming independence of the ORs using a method analogous to a meta-analysis. Tests for heterogeneity for non–case-defined factors were assessed with a $\chi 2$ test of interaction between subgroup and the binary variable

*Aggressive cancer defined as Gleason grade 8+, or prostate cancer death, or metastases or PSA >100 ng/mL
†Localized defined as TNM stage <T2 with no reported lymph node involvement or metastases or stage I; other localized stage if TNM stage T2 with no reported lymph node involvement or metastases, stage II, or equivalent; advanced stage if they were TNM stage T3 or T4 and/or N1+ and/or M1, stage III–IV, or equivalent.
‡ Low grade defined as Gleason score was <7 or equivalent (i.e. extent of differentiation good, moderate); medium grade if Gleason score was 7 (i.e. poorly differentiated); high grade if the Gleason score was ≥8 or equivalent (i.e. undifferentiated).

Abbreviations: BMI=body mass index; CI=confidence interval; OR=odds ratio; PSA=prostate-specific antigen; SD=standard deviation; SHBG=sex hormone-binding globulin; TNM= tumour, node, metastasis

	Number of cases/controls	OR (95% CI)
**Aggressive disease***	**1835/3419**	**0.97 (0.91, 1.04)**
**Age at diagnosis (yrs)**		
≤ 55	58/105	1.20 (0.69, 2.11)
56 +	1777/3314	0.97 (0.91, 1.04)
		Heterogeneity: χ^2_2=0.54 (p=0.46)
Protate cancer death	1046/2429	0.97 (0.89, 1.05)
**Stage†**		
Localized	548/877	1.01 (0.90, 1.15)
Advanced	683/944	0.98 (0.87, 1.10)
		Heterogeneity: χ^2_2=0.19 (p=0.67)
**Grade‡**		
Low	484/862	1.02 (0.89, 1.17)
High	591/702	0.95 (0.84, 1.08)
		Heterogeneity: χ^2_2=0.52 (p=0.47)
**Time to diagnosis (yrs)**		
<1	108/164	1.02 (0.74, 1.41)
1-4	636/1269	1.01 (0.89, 1.13)
5 +	1091/1986	0.95 (0.88, 1.04)
		Heterogeneity: χ^2_2=0.58 (p=0.75)
**Year of diagnosis**		
pre 1990 (pre-PSA era)	197/320	1.08 (0.87, 1.34)
1990-1994	175/296	1.03 (0.84, 1.28)
1995 onwards (PSA era)	1463/2803	0.96 (0.89, 1.03)
		Heterogeneity: χ^2_2=1.3 (p=0.51)
**Age blood draw (yrs)**		
<60	622/1027	0.95 (0.84, 1.06)
60-69	940/1925	0.99 (0.91, 1.09)
70+	273/467	0.96 (0.82, 1.12)
		Heterogeneity: χ^2_2=0.46 (p=0.79)
**BMI (kg/m²)**		
<25	559/1097	0.89 (0.80, 0.98)
25-29.9	913/1658	1.06 (0.96, 1.17)
30+	336/607	0.99 (0.83, 1.19)
		Heterogeneity: χ^2_2=6.4 (p=0.04)
**Smoking status**		
Never	582/1188	0.99 (0.89, 1.10)
Ex	792/1434	0.94 (0.84, 1.04)
Current	398/648	0.96 (0.84, 1.09)
		Heterogeneity: χ^2_2=0.6 (p=0.73)
**Alcohol consumption (g ethanol/day)**		
None	245/426	0.99 (0.86, 1.14)
1-9	479/781	1.01 (0.90, 1.15)
10+	862/1611	0.94 (0.85, 1.03)
		Heterogeneity: χ^2_2=1.2 (p=0.56)
**PSA at blood collection (ng/ml)**		
<2	275/969	0.97 (0.83, 1.13)
2-2.9	118/140	0.98 (0.70, 1.36)
3+	382/155	1.24 (0.98, 1.58)
		Heterogeneity: χ^2_2=3.3 (p=0.19)
**Time of blood collection**		
Morning	859/1382	0.96 (0.87, 1.06)
Afternoon	634/1438	1.00 (0.90, 1.11)
		Heterogeneity: χ^2_1=0.23 (p=0.63)
**Racial/ethnic group**		
White	1631/3030	0.97 (0.90, 1.04)
Other	166/285	0.94 (0.75, 1.17)
		Heterogeneity: χ^2_1=0.09 (p=0.77)
**Education status**		
No degree	1020/1568	0.96 (0.88, 1.04)
Degree	474/831	1.03 (0.90, 1.18)
		Heterogeneity: χ^2_1=0.88 (p=0.35)
**Currently married/cohabiting**		
Yes	1242/2233	0.98 (0.91, 1.07)
No	179/234	0.95 (0.79, 1.14)
		Heterogeneity: χ^2_1=0.15 (p=0.70)
**Diabetes status**		
Yes	120/235	1.03 (0.82, 1.28)
No	1440/2739	0.97 (0.90, 1.05)
		Heterogeneity: χ^2_1=0.23 (p=0.63)

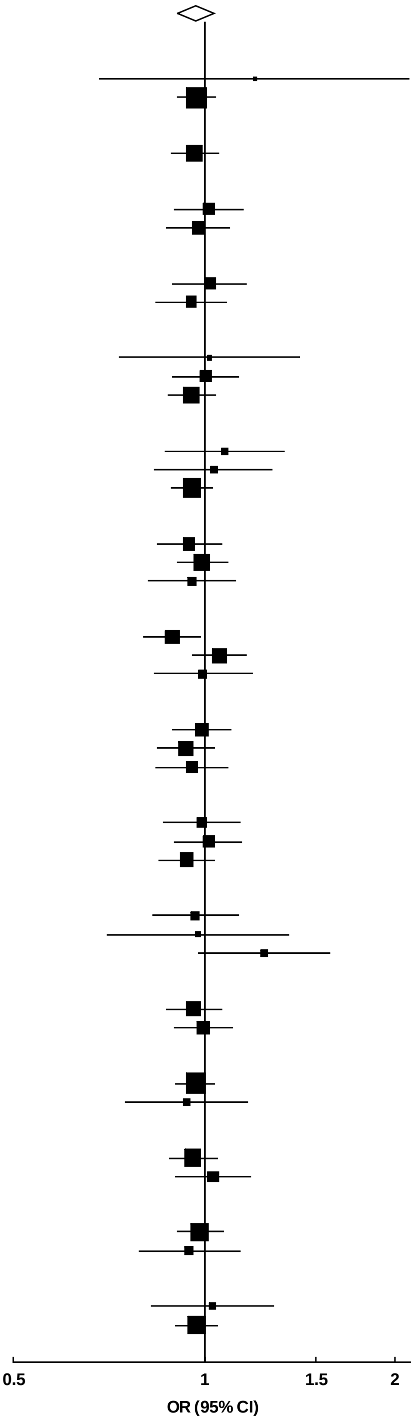

Supplementary Figure 5: Odds ratio (95% CIs) for aggressive* prostate cancer per study-specific 1 SD increment of SHBG concentration by subgroup

Estimates are from logistic regression conditioned on the matching variables and adjusted for age, BMI, height, alcohol intake, smoking status, marital status, education status, racial/ethnic group, and diabetes status. The position of each square indicates the magnitude of the OR, and the area of the square is proportional to the inverse of the variance of the logarithm of the OR. The length of the horizontal line through the square indicates the 95% CI. Tests for heterogeneity for case-defined factors were obtained by fitting separate models for each subgroup and assuming independence of the ORs using a method analogous to a meta-analysis. Tests for heterogeneity for non–case-defined factors were assessed with a $\chi 2$ test of interaction between subgroup and the binary variable

*Aggressive cancer defined as Gleason grade 8+, or prostate cancer death, or metastases or PSA >100 ng/mL
†Localized defined as TNM stage <T2 with no reported lymph node involvement or metastases or stage I, or TNM stage T2 with no reported lymph node involvement or metastases, stage II, or equivalent; advanced stage if they were TNM stage T3 or T4 and/or N1+ and/or M1, stage III–IV, or equivalent. Men with localized aggressive disease will have had high-grade prostate cancer, PSA > 100 ng/mL or died from prostate cancer.
‡ Low grade defined as Gleason score was <8 or equivalent (i.e., extent of differentiation good, moderate, poor); high grade if the Gleason score was ≥8 or equivalent (i.e., undifferentiated). Men with low-grade aggressive disease will have had advanced prostate cancer, PSA > 100 ng/mL or died from prostate cancer.
Abbreviations: BMI=body mass index; CI=confidence interval; OR=odds ratio; PSA=prostate-specific antigen; SD=standard deviation; SHBG=sex hormone-binding globulin; TNM= tumour, node, metastasis

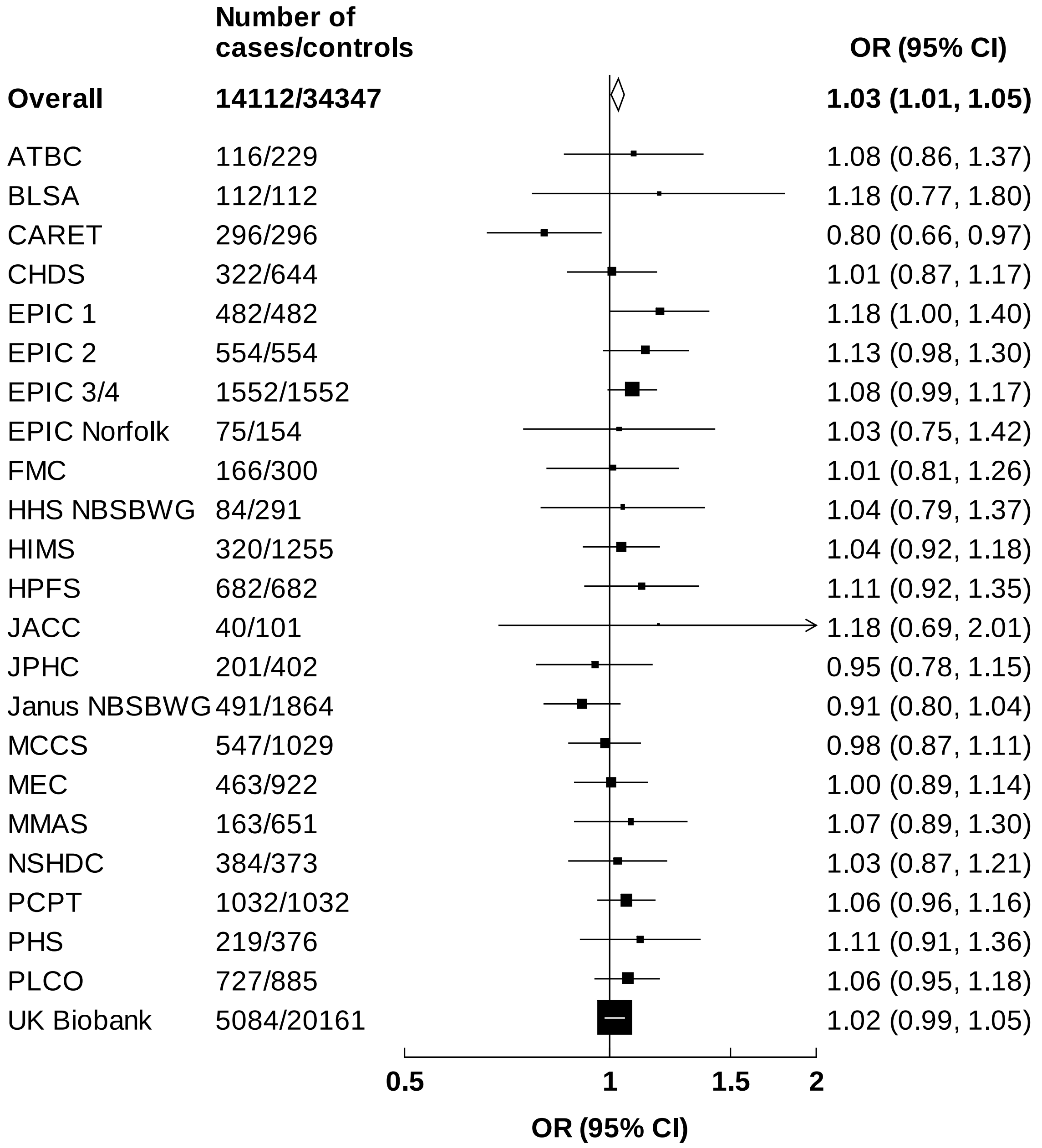

Supplementary Figure 6: Odds ratios (95% confidence intervals) for overall prostate cancer associated with a 1 SD increment in free testosterone by study

Estimates are from logistic regression conditioned on the matching variables and adjusted for age, BMI, height, alcohol intake, smoking status, marital status, education status, racial/ethnic group, and diabetes status. Heterogeneity in linear trends between studies was tested by comparing the χ^2 values for models with and without a (studies) × (linear trend) interaction term.

Test of significance (overall): p=0.01.
Test of heterogeneity (overall): χ^2_{22}=24; p=0.35.
Test of significance (studies without organised screening): p = 0.03.
Test of heterogeneity between studies without organised screening: χ^2_{20}= 23; p = 0.29.
Test of significance (studies with organised screening): p = 0.09.
Test of heterogeneity between studies with organised screening: χ^2_1 = 0.02; p = 0.88.
Test of heterogeneity between studies with and without organised screening: χ^2_1 = 0.80; p = 0.37.

Abbreviations: ATBC=The Alpha-Tocopherol, Beta-Carotene Cancer Prevention Study; BLSA= The Baltimore Longitudinal Study of Aging; CARET =The Carotene and Retinol Efficacy Trial; CHDS=Child Health and Development Studies; CI=confidence interval; EPIC=European Prospective Investigation into Cancer and Nutrition; FMC=Finnish Mobile Clinic Health Examination Survey; HHS= Helsinki Heart Study; HIMS=Health In Men Study; HPFS= Health Professionals Follow-up Study; JACC=Japan Collaborative Cohort Study; JPHC= Japan Public Health Center-based Prospective Study; MCCS=Melbourne Collaborative Cohort Study; MEC= Multiethnic Cohort Study of Diet and Cancer; MMAS=Massachusetts Male Aging Study; NBSBWG=Nordic Biological Specimen Biobank Working Group; NSHDC=Northern Sweden Health and Disease Cohort; OR=odds ratio; PCPT= Prostate Cancer Prevention Trial; PHS=Physicians' Health Study; PLCO= Prostate, Lung, Colorectal and Ovarian Cancer Screening Trial; SD=standard deviation.

	Number of cases/controls	OR (95% CI)
**Overall**	**1756/3327**	**0.96 (0.90, 1.03)**
ATBC	66/126	1.00 (0.72, 1.38)
BLSA	Insufficient data	
CARET	64/64	0.81 (0.41, 1.61)
CHDS	Insufficient data	
EPIC 1	147/147	1.47 (0.99, 2.17)
EPIC 2	120/120	1.24 (0.85, 1.80)
EPIC 3/4	271/271	1.13 (0.92, 1.37)
EPIC Norfolk	33/57	0.32 (0.08, 1.26)
FMC	57/97	0.89 (0.59, 1.36)
HHS NBSBWG	Insufficient data	
HIMS	36/111	1.24 (0.74, 2.09)
HPFS	102/102	0.87 (0.44, 1.72)
JACC	Insufficient data	
JPHC	53/106	1.07 (0.70, 1.65)
MCCS	126/201	0.86 (0.64, 1.15)
MEC	49/80	0.65 (0.30, 1.37)
NSHDC	75/72	0.73 (0.36, 1.47)
PCPT	53/53	0.84 (0.39, 1.79)
PHS	76/128	1.06 (0.58, 1.94)
PLCO	72/311	0.87 (0.60, 1.25)
UK Biobank	288/1119	0.85 (0.74, 0.98)

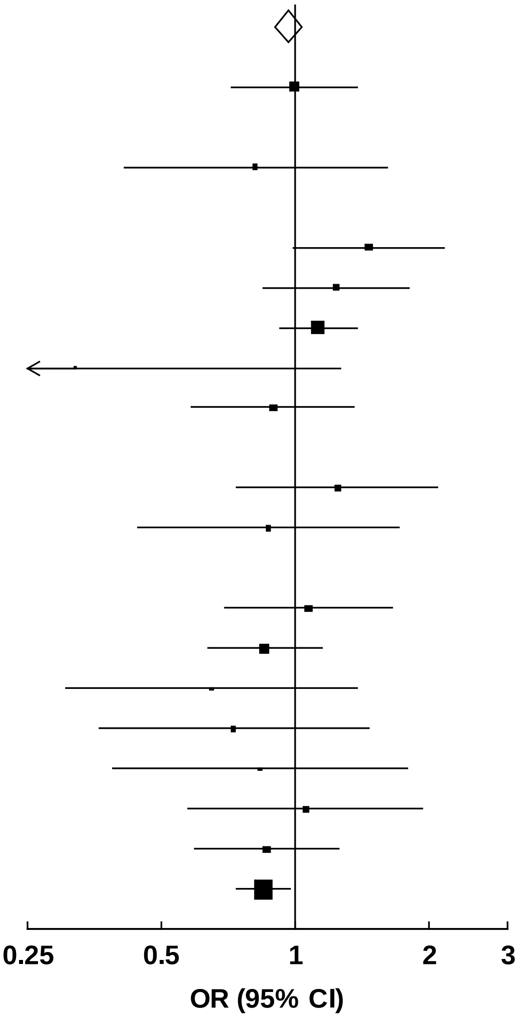

Supplementary Figure 7: Odds ratios (95% confidence intervals) for aggressive* prostate cancer associated with a 1 SD increment in free testosterone by study

Estimates are from logistic regression conditioned on the matching variables and adjusted for age, BMI, height, alcohol intake, smoking status, marital status, education status, racial/ethnic group, and diabetes status. Heterogeneity in linear trends between studies was tested by comparing the χ^2 values for models with and without a (studies) × (linear trend) interaction term.

Test of significance (overall): p=0.29.
Test of heterogeneity (overall): χ^2_{20}=35; p=0.02.
Test of significance (studies without organised screening): p = 0.47.
Test of heterogeneity between studies without organised screening: χ^2_{18}= 35; p = 0.01.
Test of significance (studies with organised screening): p = 0.25.
Test of heterogeneity between studies with organised screening: χ^2_1 = 0.38; p = 0.54.
Test of heterogeneity between studies with and without organised screening: χ^2_1 = 0.54; p = 0.46.

*Aggressive cancer defined as Gleason grade 8+, or prostate cancer death, or metastases or PSA >100 ng/mL.

Abbreviations: ATBC=The Alpha-Tocopherol, Beta-Carotene Cancer Prevention Study; BLSA= The Baltimore Longitudinal Study of Aging; CARET =The Carotene and Retinol Efficacy Trial; CHDS=Child Health and Development Studies; CI=confidence interval; EPIC=European Prospective Investigation into Cancer and Nutrition; FMC=Finnish Mobile Clinic Health Examination Survey; HHS= Helsinki Heart Study; HIMS=Health In Men Study; HPFS= Health Professionals Follow-up Study; JACC=Japan Collaborative Cohort Study; JPHC= Japan Public Health Center-based Prospective Study; MCCS=Melbourne Collaborative Cohort Study; MEC= Multiethnic Cohort Study of Diet and Cancer; NBSBWG=Nordic Biological Specimen Biobank Working Group; NSHDC=Northern Sweden Health and Disease Cohort; OR=odds ratio; PCPT= Prostate Cancer Prevention Trial; PHS=Physicians' Health Study; PLCO= Prostate, Lung, Colorectal and Ovarian Cancer Screening Trial; SD=standard deviation.

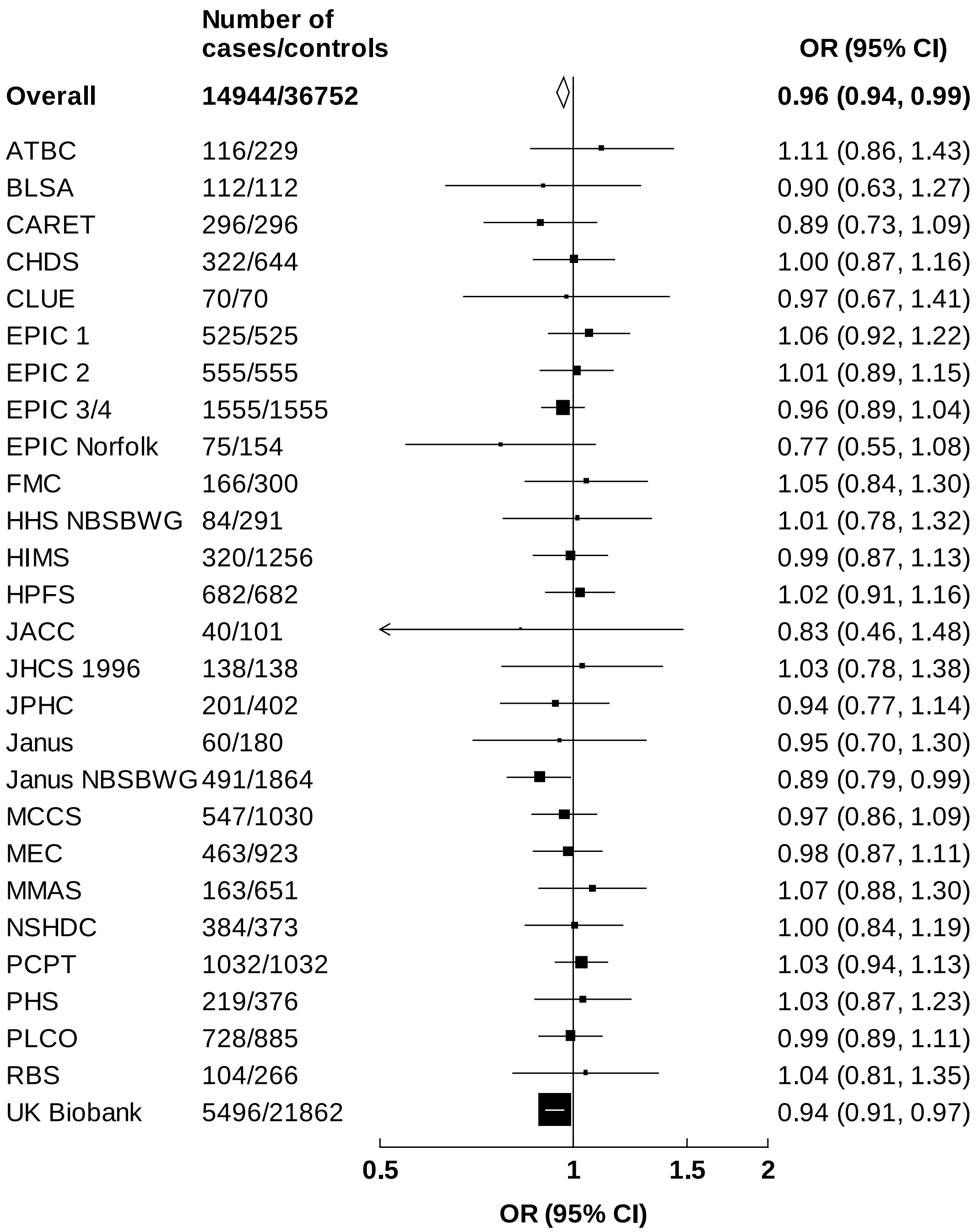

Supplementary Figure 8: Odds ratios (95% confidence intervals) for prostate cancer associated with a 1 SD increment in total testosterone by study

Estimates are from logistic regression conditioned on the matching variables and adjusted for age, BMI, height, alcohol intake, smoking status, marital status, education status, racial/ethnic group, and diabetes status. Heterogeneity in linear trends between studies was tested by comparing the χ^2 values for models with and without a (studies) × (linear trend) interaction term.
Test of significance (overall): p=0.09.
Test of heterogeneity (overall): χ^2_{22}=24; p=0.35.
Test of significance (studies without organised screening): p = 0.13.
Test of heterogeneity between studies without organised screening: χ^2_{20}= 24; p = 0.25.
Test of significance (studies with organised screening): p = 0.61.
Test of heterogeneity between studies with organised screening: χ^2_1 = 0.16; p = 0.68.
Test of heterogeneity between studies with and without organised screening: χ^2_1 = 0.05; p = 0.82.

Abbreviations: ATBC=The Alpha-Tocopherol, Beta-Carotene Cancer Prevention Study; BLSA= The Baltimore Longitudinal Study of Aging; CARET=The Carotene and Retinol Efficacy Trial; CHDS=Child Health and Development Studies; CI=confidence interval; CLUE=Give Us a Clue to Cancer and Heart Disease; EPIC=European Prospective Investigation into Cancer and Nutrition; FMC=Finnish Mobile Clinic Health Examination Survey*;* HHS= Helsinki Heart Study; HIMS=Health In Men Study; HPFS= Health Professionals Follow-up Study; JACC=Japan Collaborative Cohort Study; JPHC= Japan Public Health Center-based Prospective Study; JHCS= Japan-Hawaii Cancer Study; MCCS=Melbourne Collaborative Cohort Study; MEC=Multiethnic Cohort Study of Diet and Cancer; MMAS=Massachusetts Male Aging Study; NBSBWG=Nordic Biological Specimen Biobank Working Group; NSHDC=Northern Sweden Health and Disease Cohort; OR=odds ratio; PCPT= Prostate Cancer Prevention Trial; PHS=Physicians' Health Study; PLCO= Prostate, Lung, Colorectal and Ovarian Cancer Screening Trial; RBS=Rancho Bernardo Study; SD=standard deviation.

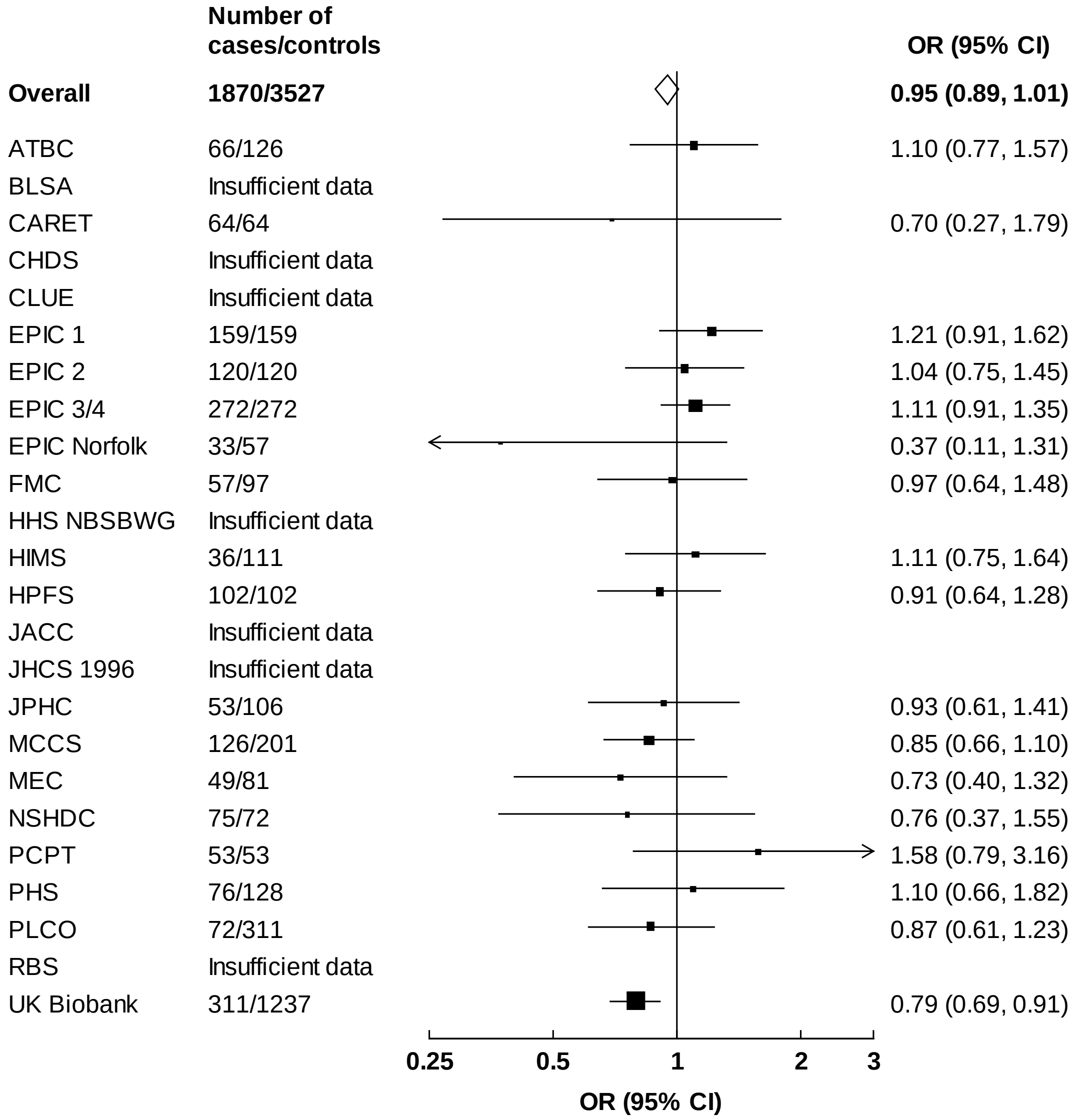

Supplementary Figure 9: Odds ratios (95% confidence intervals) for aggressive* prostate cancer associated with a 1 SD increment in total testosterone by study

Estimates are from logistic regression conditioned on the matching variables and adjusted for age, BMI, height, alcohol intake, smoking status, marital status, education status, racial/ethnic group, and diabetes status. Heterogeneity in linear trends between studies was tested by comparing the χ^2 values for models with and without a (studies) × (linear trend) interaction term.

Test of significance (overall): p = 0.09.

Test of heterogeneity (overall): χ^2_{23}=22; p=0.52.

Test of significance (studies without organised screening): p=0.12.

Test of heterogeneity between studies without organised screening: χ^2_{21}= 22; p = 0.40.

Test of significance (studies with organised screening): p = 0.61.

Test of heterogeneity between studies with organised screening: χ^2_1 = 0.2; p = 0.69.

Test of heterogeneity between studies with and without organised screening: χ^2_1 = 0.04; p = 0.85.

*Aggressive cancer defined as Gleason grade 8+, or prostate cancer death, or metastases or PSA >100 ng/mL.

Abbreviations: ATBC=The Alpha-Tocopherol, Beta-Carotene Cancer Prevention Study; BLSA=The Baltimore Longitudinal Study of Aging; CARET=The Carotene and Retinol Efficacy Trial; CHDS=Child Health and Development Studies; CI=confidence interval; CLUE=Give Us a Clue to Cancer and Heart Disease; EPIC=European Prospective Investigation into Cancer and Nutrition; FMC=Finnish Mobile Clinic Health Examination Survey*;* HHS= Helsinki Heart Study; HIMS=Health In Men Study; HPFS= Health Professionals Follow-up Study; JACC=Japan Collaborative Cohort Study; JPHC= Japan Public Health Center-based Prospective Study; JHCS= Japan-Hawaii Cancer Study; MCCS=Melbourne Collaborative Cohort Study; MEC=Multiethnic Cohort Study of Diet and Cancer; NBSBWG=Nordic Biological Specimen Biobank Working Group; NSHDC=Northern Sweden Health and Disease Cohort; OR=odds ratio; PCPT= Prostate Cancer Prevention Trial; PHS=Physicians' Health Study; PLCO= Prostate, Lung, Colorectal and Ovarian Cancer Screening Trial; SD=standard deviation.

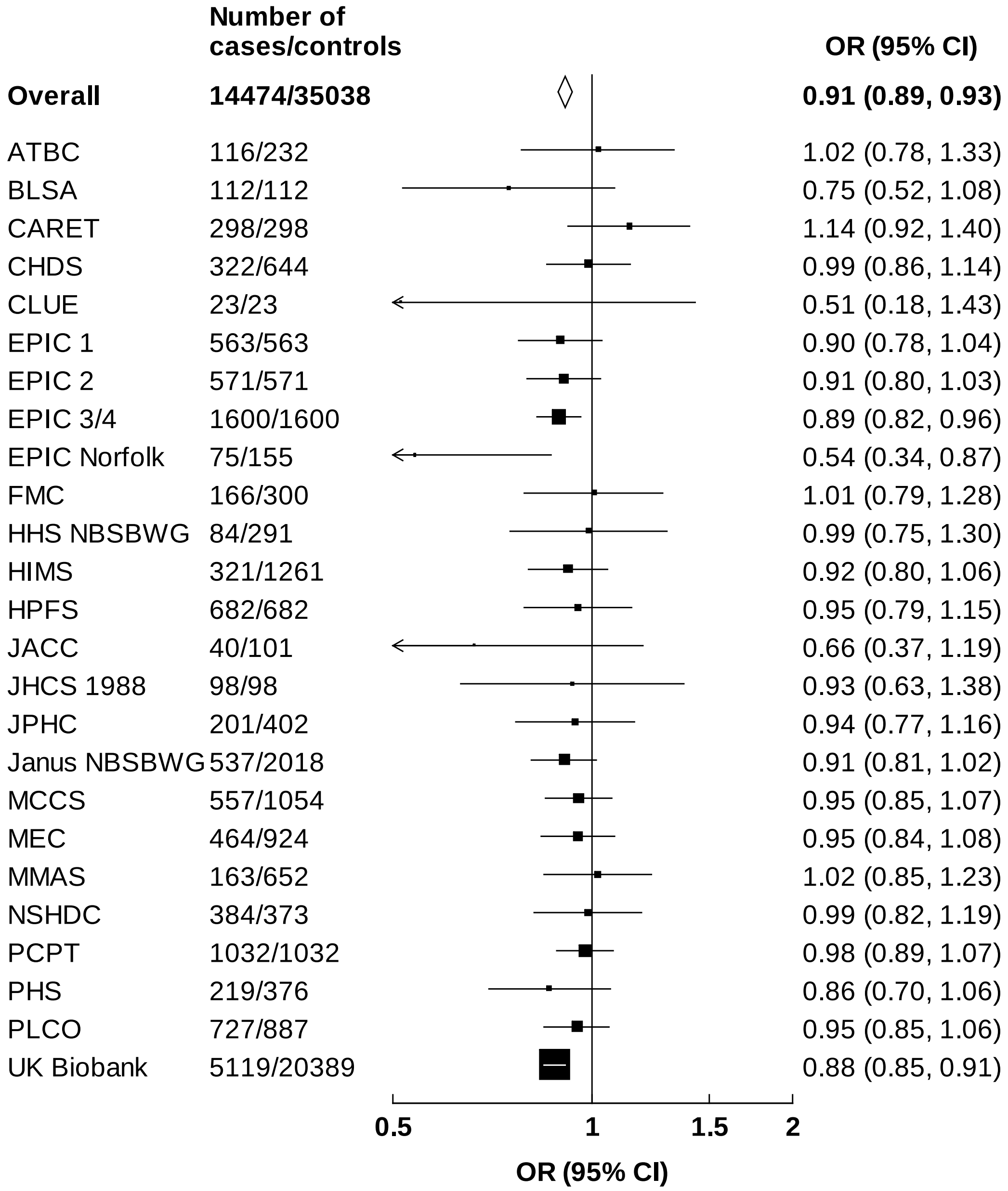

Supplementary Figure 10: Odds ratios (95% confidence intervals) for prostate cancer associated with a 1 SD increment in sex hormone-binding globulin by study

Estimates are from logistic regression conditioned on the matching variables and adjusted for age, BMI, height, alcohol intake, smoking status, marital status, education status, racial/ethnic group, and diabetes status. Heterogeneity in linear trends between studies was tested by comparing the χ^2 values for models with and without a (studies) × (linear trend) interaction term.

Test of significance (overall): $p < 0.0001$.
Test of heterogeneity (overall): χ^2_{24}=17; p=0.83.
Test of significance (studies without organised screening): $p < 0.0001$.
Test of heterogeneity between studies without organised screening: χ^2_{22}= 13; p = 0.92.
Test of significance (studies with organised screening): p = 0.31.
Test of heterogeneity between studies with organised screening: χ^2_1 = 0.37; p = 0.54.
Test of heterogeneity between studies with and without organised screening: χ^2_1 = 3.4; p = 0.07.

Abbreviations: ATBC=The Alpha-Tocopherol, Beta-Carotene Cancer Prevention Study; BLSA=The Baltimore Longitudinal Study of Aging; CARET=The Carotene and Retinol Efficacy Trial; CHDS=Child Health and Development Studies; CI=confidence interval; CLUE=Give Us a Clue to Cancer and Heart Disease; EPIC=European Prospective Investigation into Cancer and Nutrition; FMC=Finnish Mobile Clinic Health Examination Survey; HHS= Helsinki Heart Study; HIMS=Health In Men Study; HPFS= Health Professionals Follow-up Study; JACC=Japan Collaborative Cohort Study; JPHC= Japan Public Health Center-based Prospective Study; JHCS= Japan-Hawaii Cancer Study; MCCS=Melbourne Collaborative Cohort Study; MEC=Multiethnic Cohort Study of Diet and Cancer; MMAS=Massachusetts Male Aging Study; NBSBWG=Nordic Biological Specimen Biobank Working Group; NSHDC=Northern Sweden Health and Disease Cohort; OR=odds ratio; PCPT= Prostate Cancer Prevention Trial; PHS=Physicians' Health Study; PLCO= Prostate, Lung, Colorectal and Ovarian Cancer Screening Trial; SD=standard deviation.

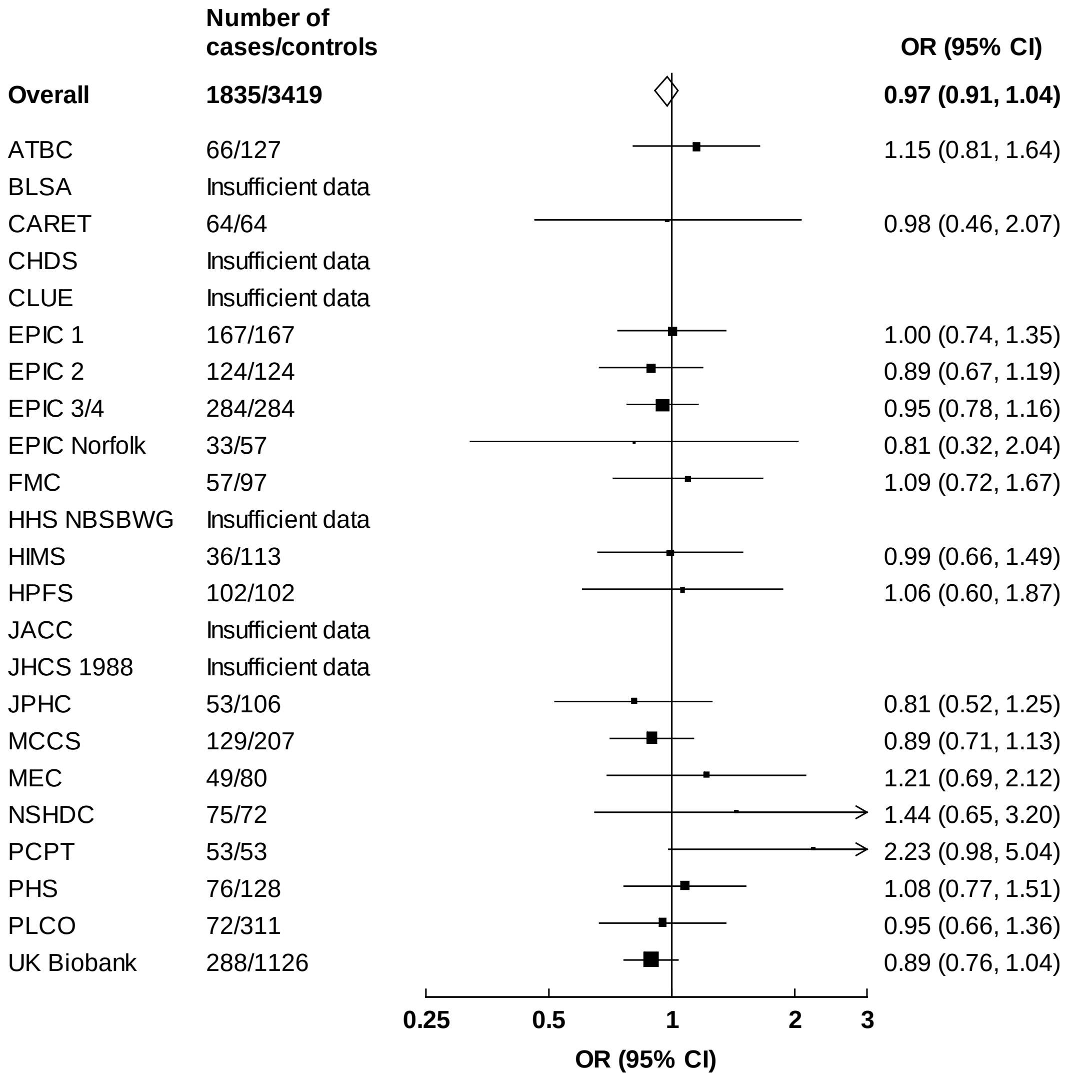

Supplementary Figure 11: Odds ratios (95% confidence intervals) for aggressive* prostate cancer associated with a 1 SD increment in sex hormone-binding globulin by study
Estimates are from logistic regression conditioned on the matching variables and adjusted for age, BMI, height, alcohol intake, smoking status, marital status, education status, racial/ethnic group, and diabetes status. Heterogeneity in linear trends between studies was tested by comparing the χ^2 values for models with and without a (studies) × (linear trend) interaction term.

Test of significance (overall): p = 0.40.
Test of heterogeneity (overall): χ^2_{22}=17; p=0.79.
Test of significance (studies without organised screening): p=0.29.
Test of heterogeneity between studies without organised screening: χ^2_{20}= 18; p = 0.62.
Test of significance (studies with organised screening): p = 0.30.
Test of heterogeneity between studies with organised screening: χ^2_1 = 1.1; p = 0.30.
Test of heterogeneity between studies with and without organised screening: χ^2_1 = 0.5; p = 0.49.

*Aggressive cancer defined as Gleason grade 8+, or prostate cancer death, or metastases or PSA >100 ng/mL.

Abbreviations: ATBC=The Alpha-Tocopherol, Beta-Carotene Cancer Prevention Study; BLSA=The Baltimore Longitudinal Study of Aging; CARET=The Carotene and Retinol Efficacy Trial; CHDS=Child Health and Development Studies; CI=confidence interval; CLUE=Give Us a Clue to Cancer and Heart Disease; EPIC=European Prospective Investigation into Cancer and Nutrition; FMC=Finnish Mobile Clinic Health Examination Survey*;* HHS= Helsinki Heart Study; HIMS=Health In Men Study; HPFS= Health Professionals Follow-up Study; JACC=Japan Collaborative Cohort Study; JPHC= Japan Public Health Center-based Prospective Study; JHCS= Japan-Hawaii Cancer Study; MCCS=Melbourne Collaborative Cohort Study; MEC=Multiethnic Cohort Study of Diet and Cancer; NBSBWG=Nordic Biological Specimen Biobank Working Group; NSHDC=Northern Sweden Health and Disease Cohort; OR=odds ratio; PCPT= Prostate Cancer Prevention Trial; PHS=Physicians' Health Study; PLCO= Prostate, Lung, Colorectal and Ovarian Cancer Screening Trial; SD=standard deviation.

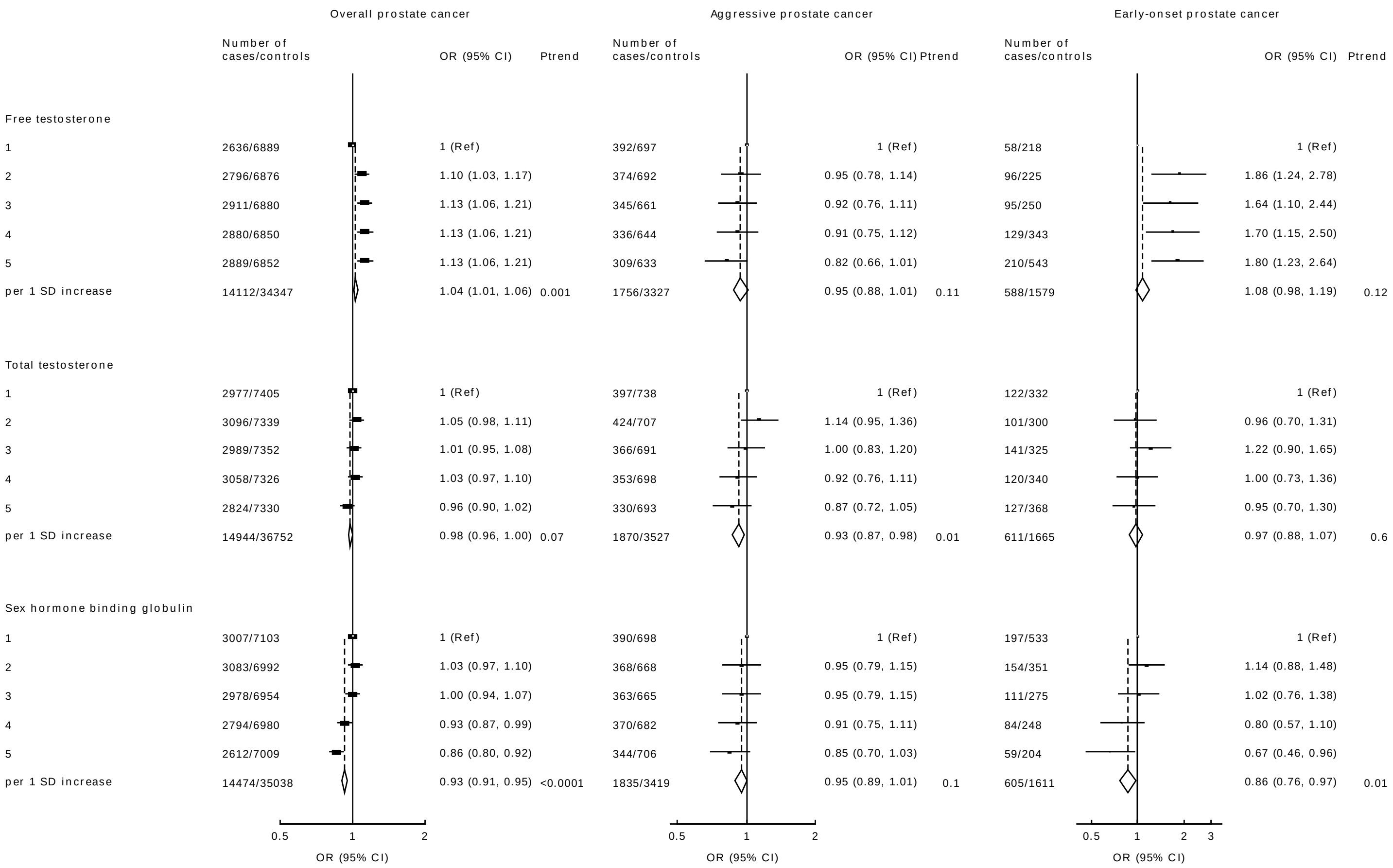

	Overall prostate cancer			Aggressive prostate cancer			Early-onset prostate cancer		
	Number of cases/controls	OR (95% CI)	Ptrend	Number of cases/controls	OR (95% CI)	Ptrend	Number of cases/controls	OR (95% CI)	Ptrend
**Free testosterone**									
1	2636/6889	1 (Ref)		392/697	1 (Ref)		58/218	1 (Ref)	
2	2796/6876	1.10 (1.03, 1.17)		374/692	0.95 (0.78, 1.14)		96/225	1.86 (1.24, 2.78)	
3	2911/6880	1.13 (1.06, 1.21)		345/661	0.92 (0.76, 1.11)		95/250	1.64 (1.10, 2.44)	
4	2880/6850	1.13 (1.06, 1.21)		336/644	0.91 (0.75, 1.12)		129/343	1.70 (1.15, 2.50)	
5	2889/6852	1.13 (1.06, 1.21)		309/633	0.82 (0.66, 1.01)		210/543	1.80 (1.23, 2.64)	
per 1 SD increase	14112/34347	1.04 (1.01, 1.06)	0.001	1756/3327	0.95 (0.88, 1.01)	0.11	588/1579	1.08 (0.98, 1.19)	0.12
**Total testosterone**									
1	2977/7405	1 (Ref)		397/738	1 (Ref)		122/332	1 (Ref)	
2	3096/7339	1.05 (0.98, 1.11)		424/707	1.14 (0.95, 1.36)		101/300	0.96 (0.70, 1.31)	
3	2989/7352	1.01 (0.95, 1.08)		366/691	1.00 (0.83, 1.20)		141/325	1.22 (0.90, 1.65)	
4	3058/7326	1.03 (0.97, 1.10)		353/698	0.92 (0.76, 1.11)		120/340	1.00 (0.73, 1.36)	
5	2824/7330	0.96 (0.90, 1.02)		330/693	0.87 (0.72, 1.05)		127/368	0.95 (0.70, 1.30)	
per 1 SD increase	14944/36752	0.98 (0.96, 1.00)	0.07	1870/3527	0.93 (0.87, 0.98)	0.01	611/1665	0.97 (0.88, 1.07)	0.6
**Sex hormone binding globulin**									
1	3007/7103	1 (Ref)		390/698	1 (Ref)		197/533	1 (Ref)	
2	3083/6992	1.03 (0.97, 1.10)		368/668	0.95 (0.79, 1.15)		154/351	1.14 (0.88, 1.48)	
3	2978/6954	1.00 (0.94, 1.07)		363/665	0.95 (0.79, 1.15)		111/275	1.02 (0.76, 1.38)	
4	2794/6980	0.93 (0.87, 0.99)		370/682	0.91 (0.75, 1.11)		84/248	0.80 (0.57, 1.10)	
5	2612/7009	0.86 (0.80, 0.92)		344/706	0.85 (0.70, 1.03)		59/204	0.67 (0.46, 0.96)	
per 1 SD increase	14474/35038	0.93 (0.91, 0.95)	<0.0001	1835/3419	0.95 (0.89, 1.01)	0.1	605/1611	0.86 (0.76, 0.97)	0.01

Supplementary Figure 12: Risks of overall, aggressive and early-onset prostate cancer* by study-specific fifths of biomarker concentrations and 1 SD increment in the unadjusted model.

Estimates are from logistic regression conditioned on the matching variables. The position of each square indicates the magnitude of the OR, and the area of the square is proportional to the inverse of the variance of the logarithm of the OR. The length of the horizontal line through the square indicates the 95% confidence interval.

*Aggressive cancer defined as Gleason grade 8+, or prostate cancer death, or metastases or PSA >100 ng/mL. Early-onset defined as diagnosed ≤55 years

Abbreviations: BMI=body mass index; CI=confidence interval; OR=odds ratio; PSA=prostate-specific antigen; SD=standard deviation.

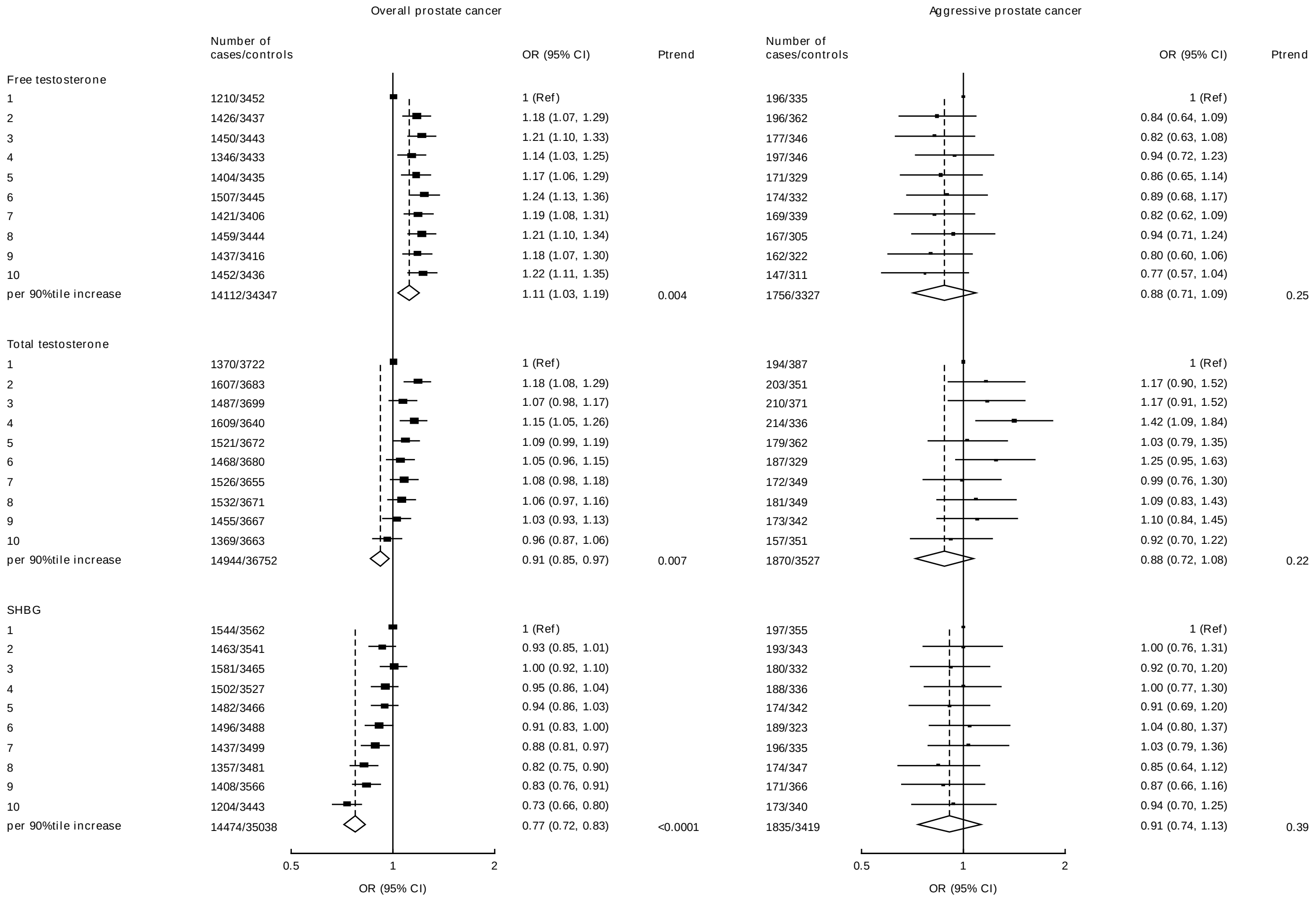

Supplementary Figure 13: Risks of overall and aggressive* prostate cancer by study-specific tenths of biomarker concentrations and 90%tile increment.

Estimates are from logistic regression conditioned on the matching variables and adjusted for age, BMI, height, alcohol intake, smoking status, marital status, education status, racial/ethnic group, and diabetes status. The categorical variables representing the study-specific tenths of the biomarker concentrations was replaced with a continuous variable that was scored as 0, 0.11, 0.22, 0.33, 0.44, 0.56 0.67, 0.78, 0.89 and 1; because the mid-points of the lowest and highest tenths are the 10th and 90th percentiles of the study-specific biomarker concentrations, a unit increase in this variable can be taken to represent an 90 percentile increase in the biomarker study-specific concentration. The position of each square indicates the magnitude of the odds ratio, and the area of the square is proportional to the inverse of the variance of the logarithm of the OR. The length of the horizontal line through the square indicates the 95% CI.

*Aggressive cancer defined as Gleason grade 8+, or prostate cancer death, or metastases or PSA >100 ng/mL.

Abbreviations: BMI=body mass index; CI=confidence interval; OR=odds ratio; PSA=prostate specific antigen; SHBG=sex hormone–binding globulin.

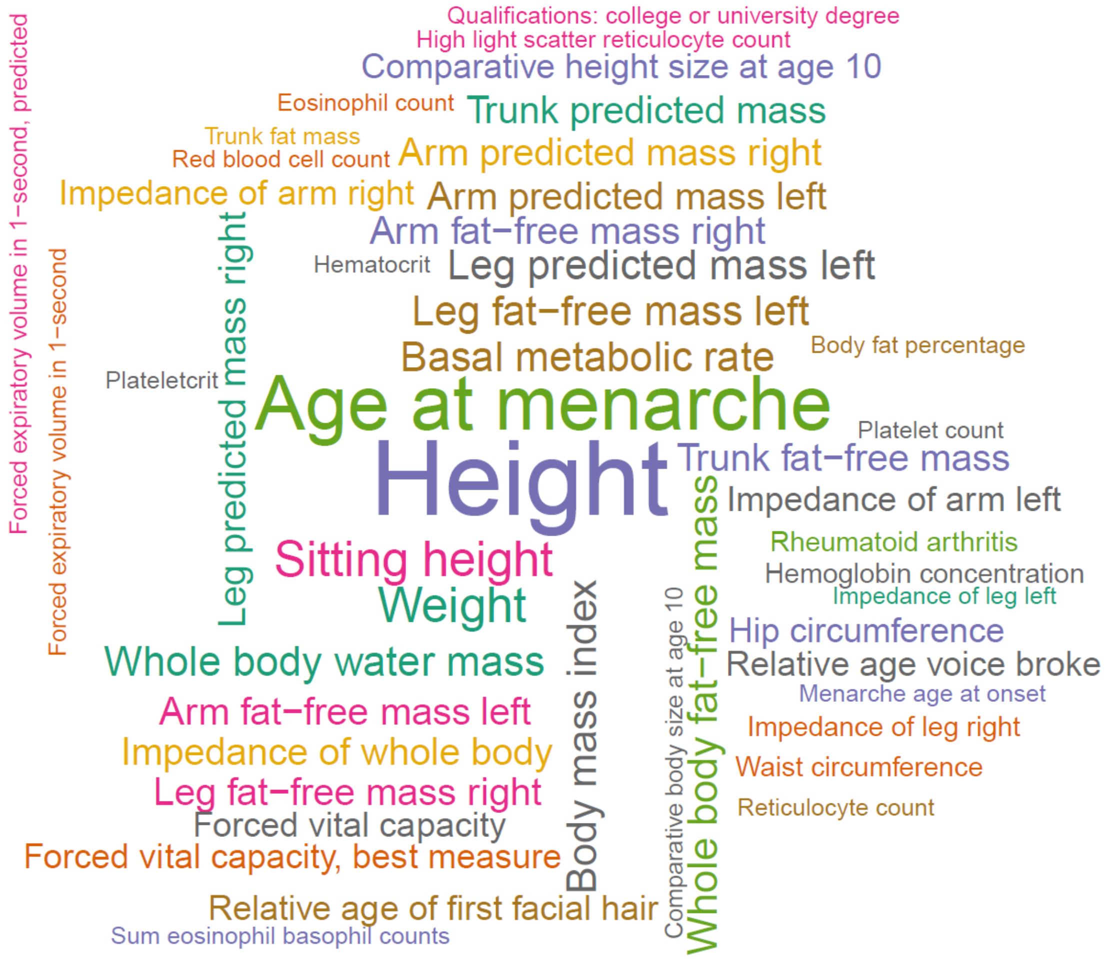

Supplementary Figure 14: Traits associated with each free testosterone associated SNP used in the Mendelian randomization analyses

Traits were identified using PhenoScanner resource [1, 2]. Larger words represent a greater frequency of the traits being associated with the SNPs (P threshold=5 x 10^{-8}). This figure was created using the wordcloud package in R.

Abbreviations: SNP=single nucleotide polymorphism.

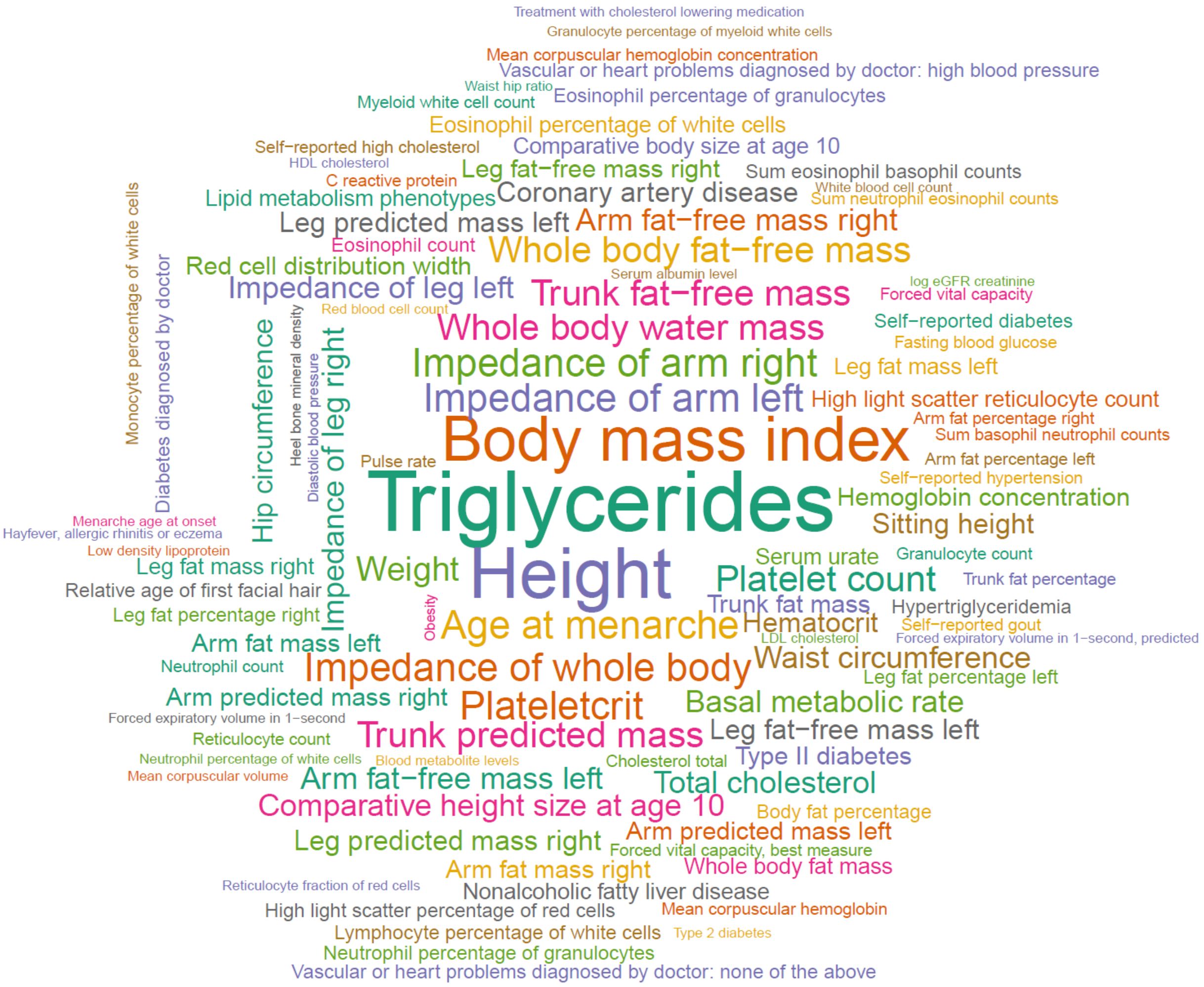

Supplementary Figure 15: Traits associated with each total testosterone associated SNP used in the Mendelian randomization analyses

Traits were identified using PhenoScanner resource [1, 2]. Larger words represent a greater frequency of the traits being associated with the SNPs (P threshold=5 x 10^{-8}). This figure was created using the wordcloud package in R.

Abbreviations: SNP=single nucleotide polymorphism.

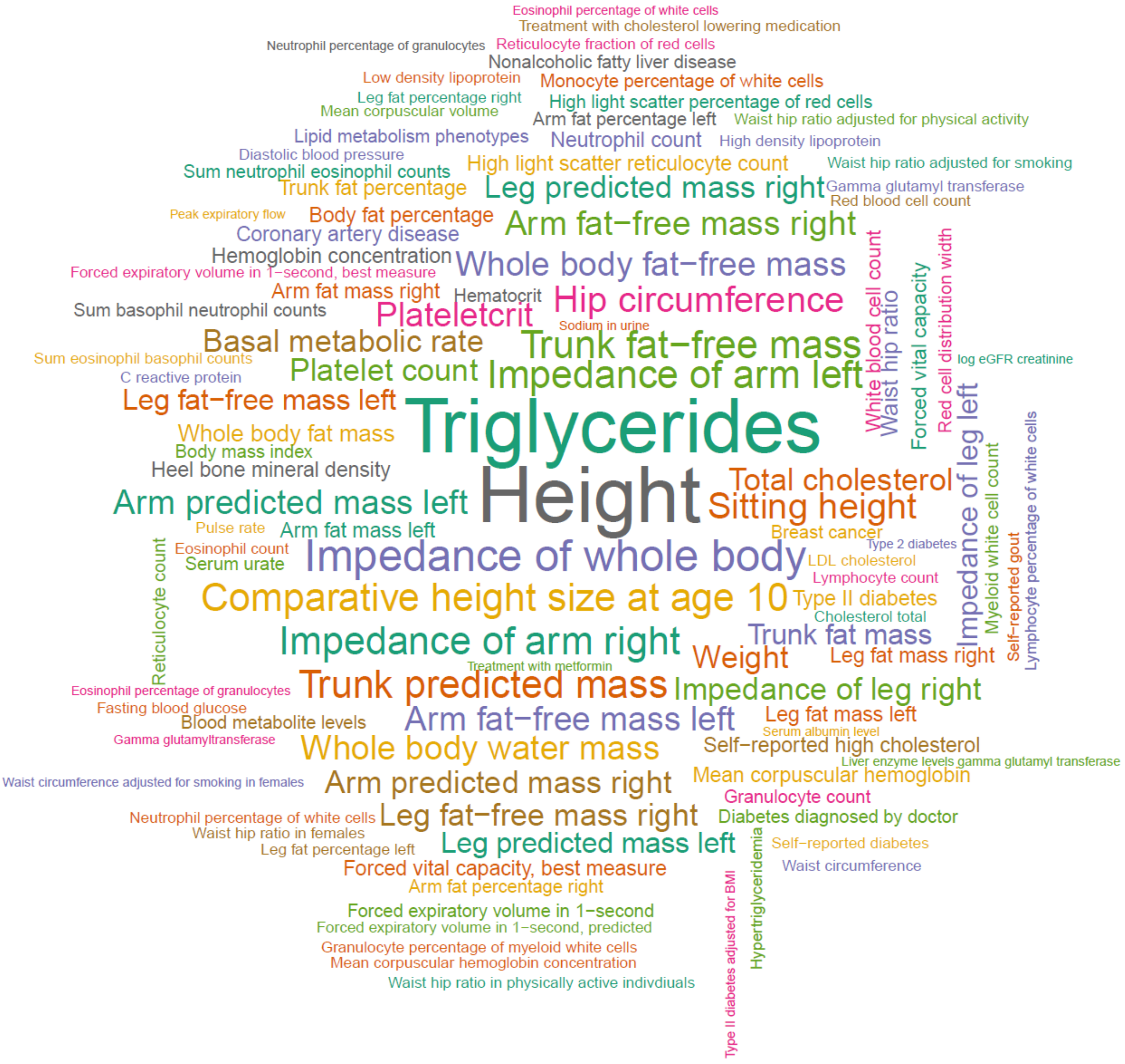

Supplementary Figure 16: Traits associated with each SHBG associated SNP used in the Mendelian randomization analyses

Traits were identified using PhenoScanner resource [1, 2]. Larger words represent a greater frequency of the traits being associated with the SNPs (P threshold=5 x 10^{-8}). This figure was created using the wordcloud package in R.

Abbreviations: Sex hormone-binding globulin; SNP=single nucleotide polymorphism.

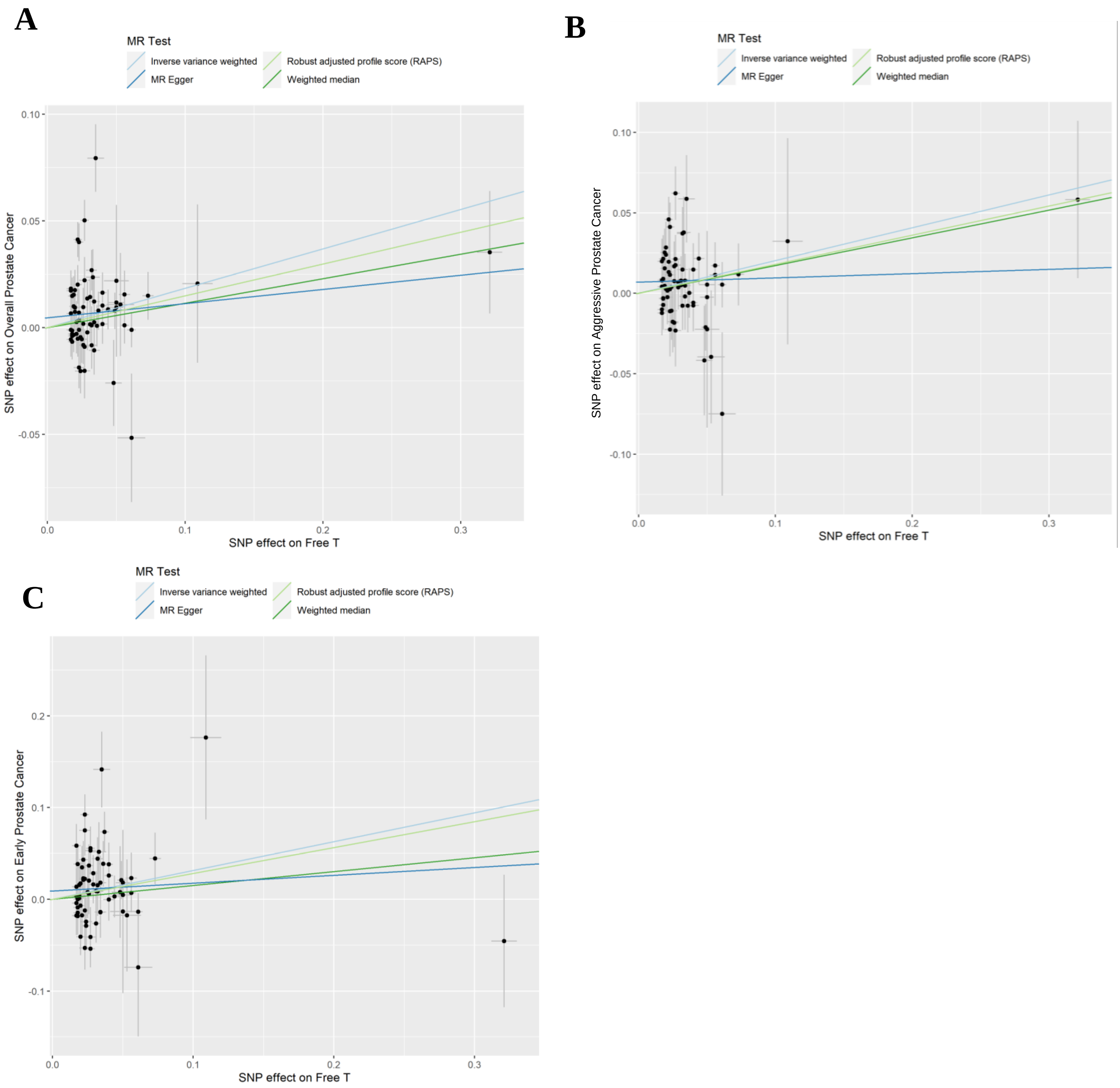

Supplementary Figure 17: Scatterplot of genetic associations with free testosterone against genetic associations with:
A) Overall prostate cancer
B) Aggressive prostate cancer
C) Early-onset prostate cancer
Aggressive cancer defined as Gleason grade 8+, or prostate cancer death, or metastases or PSA >100 ng/mL, early-onset defined as diagnosed ≤55 years. Point estimates represent log odds ratios. Error bars represent 95% confidence intervals.

Abbreviations: PSA=prostate-specific antigen; SNP=single nucleotide repeat polymorphism.

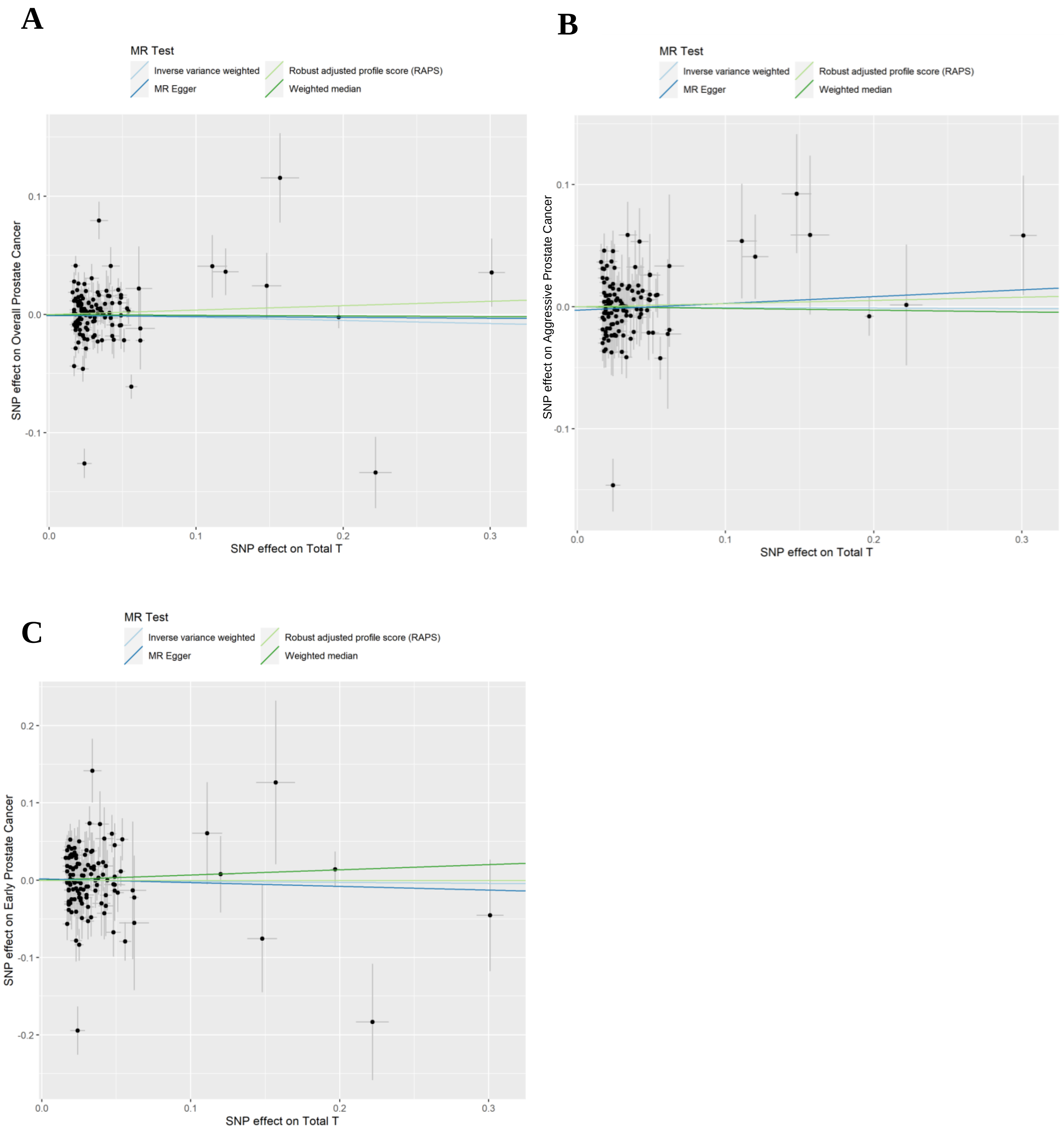

Supplementary Figure 18: Scatterplot of genetic associations with total testosterone against genetic associations with:
A) Overall prostate cancer
B) Aggressive prostate cancer
C) Early-onset prostate cancer
Aggressive cancer defined as Gleason grade 8+, or prostate cancer death, or metastases or PSA >100 ng/mL, early-onset defined as diagnosed ≤55 years. Point estimates represent log odds ratios. Error bars represent 95% confidence intervals.

Abbreviations: PSA=prostate-specific antigen; SNP=single nucleotide repeat polymorphism.

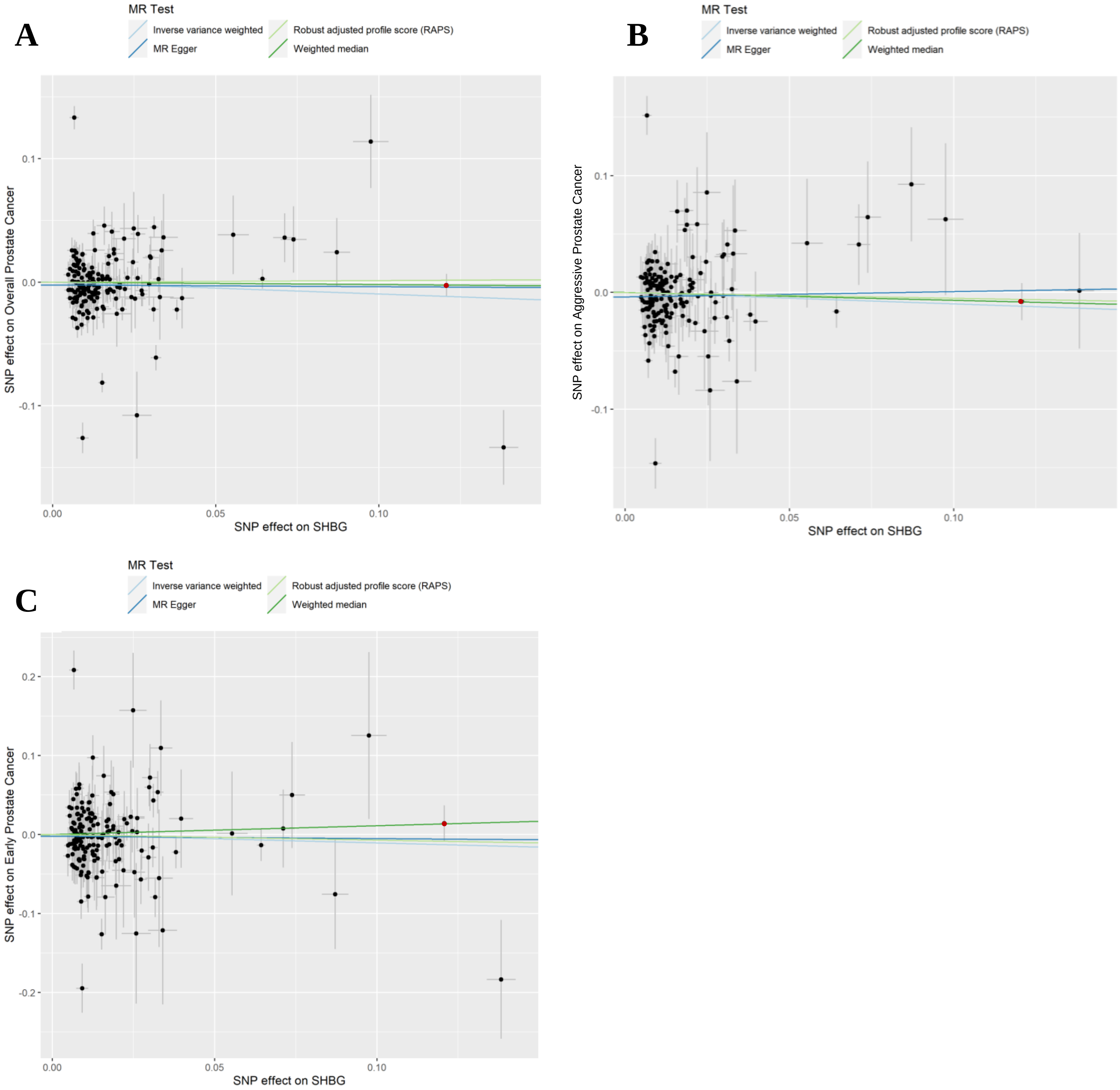

Supplementary Figure 19: Scatterplot of genetic associations with SHBG against genetic associations with:
A) Overall prostate cancer
B) Aggressive prostate cancer
C) Early-onset prostate cancer
Aggressive cancer defined as Gleason grade 8+, or prostate cancer death, or metastases or PSA >100 ng/mL, early-onset defined as diagnosed ≤55 years. Point estimates represent log odds ratios. Error bars represent 95% confidence intervals, *cis*-SNP coloured red.

Abbreviations: PSA=prostate-specific antigen; SHBG=sex hormone-binding globulin; SNP=single nucleotide repeat polymorphism.

References

1. Kamat MA, Blackshaw JA, Young R, Surendran P, Burgess S, Danesh J, Butterworth AS, Staley JR. PhenoScanner V2: an expanded tool for searching human genotype-phenotype associations. *Bioinformatics* 2019;**35**: 4851-3.
2. Staley JR, Blackshaw J, Kamat MA, Ellis S, Surendran P, Sun BB, Paul DS, Freitag D, Burgess S, Danesh J, Young R, Butterworth AS. PhenoScanner: a database of human genotype-phenotype associations. *Bioinformatics (Oxford, England)* 2016;**32**: 3207-9.
3. Roddam AW, Allen NE, Appleby P, Key TJ. Endogenous sex hormones and prostate cancer: a collaborative analysis of 18 prospective studies. *Journal of the National Cancer Institute* 2008;**100**: 170-83.
4. Dorgan JF, Albanes D, Virtamo J, Heinonen OP, Chandler DW, Galmarini M, McShane LM, Barrett MJ, Tangrea J, Taylor PR. Relationships of serum androgens and estrogens to prostate cancer risk: results from a prospective study in Finland. *Cancer epidemiology, biomarkers & prevention : a publication of the American Association for Cancer Research, cosponsored by the American Society of Preventive Oncology* 1998;**7**: 1069-74.
5. Carter HB, Pearson JD, Metter EJ, Chan DW, Andres R, Fozard JL, Rosner W, Walsh PC. Longitudinal evaluation of serum androgen levels in men with and without prostate cancer. *Prostate* 1995;**27**: 25-31.
6. Parsons JK, Carter HB, Platz EA, Wright EJ, Landis P, Metter EJ. Serum testosterone and the risk of prostate cancer: potential implications for testosterone therapy. *Cancer epidemiology, biomarkers & prevention : a publication of the American Association for Cancer Research, cosponsored by the American Society of Preventive Oncology* 2005;**14**: 2257-60.
7. Chen C, Weiss NS, Stanczyk FZ, Lewis SK, DiTommaso D, Etzioni R, Barnett MJ, Goodman GE. Endogenous sex hormones and prostate cancer risk: a case-control study nested within the Carotene and Retinol Efficacy Trial. *Cancer Epidemiol Biomarkers Prev* 2003;**12**: 1410-6.
8. Tsai CJ, Cohn BA, Cirillo PM, Feldman D, Stanczyk FZ, Whittemore AS. Sex steroid hormones in young manhood and the risk of subsequent prostate cancer: a longitudinal study in African-Americans and Caucasians (United States). *Cancer Causes Control* 2006;**17**: 1237-44.
9. van den Berg BJ, Christianson RE, Oechsli FW. The California Child Health and Development Studies of the School of Public Health, University of California at Berkeley. *Paediatr Perinat Epidemiol* 1988;**2**: 265-82.
10. Hsing AW, Comstock GW. Serological precursors of cancer: serum hormones and risk of subsequent prostate cancer. *Cancer epidemiology, biomarkers & prevention : a publication of the American Association for Cancer Research, cosponsored by the American Society of Preventive Oncology* 1993;**2**: 27-32.
11. Travis RC, Key TJ, Allen NE, Appleby PN, Roddam AW, Rinaldi S, Egevad L, Gann PH, Rohrmann S, Linseisen J, Pischon T, Boeing H, et al. Serum androgens and prostate cancer among 643 cases and 643 controls in the European Prospective Investigation into Cancer and Nutrition. *Int J Cancer* 2007;**121**: 1331-8.
12. Low Y-L, Taylor JI, Grace PB, Dowsett M, Folkerd E, Doody D, Dunning AM, Scollen S, Mulligan AA, Welch AA, Luben RN, Khaw K-T, et al. Polymorphisms in the CYP19 Gene May Affect the Positive Correlations between Serum and Urine Phytoestrogen Metabolites and Plasma Androgen Concentrations in Men. *J Nutr* 2005;**135**: 2680-6.
13. Heikkila R, Aho K, Heliovaara M, Hakama M, Marniemi J, Reunanen A, Knekt P. Serum testosterone and sex hormone-binding globulin concentrations and the risk of prostate carcinoma: a longitudinal study. *Cancer* 1999;**86**: 312-5.
14. Stattin P, Lumme S, Tenkanen L, Alfthan H, Jellum E, Hallmans G, Thoresen S, Hakulinen T, Luostarinen T, Lehtinen M, Dillner J, Stenman UH, et al. High levels of circulating testosterone are not associated with increased prostate cancer risk: a pooled prospective study. *International journal of cancer* 2004;**108**: 418-24.
15. Manttari M, Elo O, Frick MH, Haapa K, Heinonen OP, Heinsalmi P, Helo P, Huttunen JK, Kaitaniemi P, Koskinen P, et al. The Helsinki Heart Study: basic design and randomization procedure. *Eur Heart J* 1987;**8 Suppl I**: 1-29.
16. Hyde Z, Flicker L, McCaul KA, Almeida OP, Hankey GJ, Chubb SAP, Yeap BB. Associations between testosterone levels and incident prostate, lung, and colorectal cancer. A population-based study. *Cancer Epidemiol Biomarkers Prev* 2012;**21**: 1319-29.
17. Chan YX, Alfonso H, Chubb SA, Handelsman DJ, Fegan PG, Hankey GJ, Golledge J, Flicker L, Yeap BB. Higher dihydrotestosterone is associated with the incidence of lung cancer in older men. *Horm Cancer* 2017;**8**: 119-26.
18. Norman PE, Flicker L, Almeida OP, Hankey GJ, Hyde Z, Jamrozik K. Cohort Profile: The Health In Men Study (HIMS). *Int J Epidemiol* 2009;**38**: 48-52.
19. Platz EA, Leitzmann MF, Rifai N, Kantoff PW, Chen YC, Stampfer MJ, Willett WC, Giovannucci E. Sex steroid hormones and the androgen receptor gene CAG repeat and subsequent risk of prostate cancer in the prostate-specific antigen era. *Cancer Epidemiol Biomarkers Prev* 2005;**14**: 1262-9.

20. Ozasa K, Nakao M, Watanabe Y, Hayashi K, Miki T, Mikami K, Mori M, Sakauchi F, Washio M, Ito Y, Suzuki K, Wakai K, et al. Serum phytoestrogens and prostate cancer risk in a nested case-control study among Japanese men. *Cancer Sci* 2004;**95**: 65-71.

21. Nomura A, Heilbrun LK, Stemmermann GN, Judd HL. Prediagnostic serum hormones and the risk of prostate cancer. *Cancer research* 1988;**48**: 3515-7.

22. Nomura AM, Stemmermann GN, Chyou PH, Henderson BE, Stanczyk FZ. Serum androgens and prostate cancer. *Cancer epidemiology, biomarkers & prevention : a publication of the American Association for Cancer Research, cosponsored by the American Society of Preventive Oncology* 1996;**5**: 621-5.

23. Sawada N, Iwasaki M, Inoue M, Sasazuki S, Yamaji T, Shimazu T, Tsugane S. Plasma testosterone and sex hormone-binding globulin concentrations and the risk of prostate cancer among Japanese men: a nested case-control study. *Cancer Sci* 2010;**101**: 2652-7.

24. Vatten LJ, Ursin G, Ross RK, Stanczyk FZ, Lobo RA, Harvei S, Jellum E. Androgens in serum and the risk of prostate cancer: a nested case-control study from the Janus serum bank in Norway. *Cancer epidemiology, biomarkers & prevention : a publication of the American Association for Cancer Research, cosponsored by the American Society of Preventive Oncology* 1997;**6**: 967-9.

25. Severi G, Morris HA, MacInnis RJ, English DR, Tilley W, Hopper JL, Boyle P, Giles GG. Circulating steroid hormones and the risk of prostate cancer. *Cancer Epidemiol Biomarkers Prev* 2006;**15**: 86-91.

26. Gill JK, Wilkens LR, Pollak MN, Stanczyk FZ, Kolonel LN. Androgens, growth factors and risk of prostate cancer: the Multiethnic Cohort. *Prostate* 2010;**70**: 906-15.

27. Mohr BA, Feldman HA, Kalish LA, Longcope C, McKinlay JB. Are serum hormones associated with the risk of prostate cancer? Prospective results from the Massachusetts Male Aging Study. *Urology* 2001;**57**: 930-5.

28. Thompson IM, Goodman PJ, Tangen CM, Lucia MS, Miller GJ, Ford LG, Lieber MM, Cespedes RD, Atkins JN, Lippman SM, Carlin SM, Ryan A, et al. The influence of finasteride on the development of prostate cancer. *N Engl J Med* 2003;**349**: 215-24.

29. Gann PH, Hennekens CH, Ma J, Longcope C, Stampfer MJ. Prospective study of sex hormone levels and risk of prostate cancer. *J Natl Cancer Inst* 1996;**88**: 1118-26.

30. Andriole GL, Crawford ED, Grubb RL, 3rd, Buys SS, Chia D, Church TR, Fouad MN, Isaacs C, Kvale PA, Reding DJ, Weissfeld JL, Yokochi LA, et al. Prostate cancer screening in the randomized Prostate, Lung, Colorectal, and Ovarian Cancer Screening Trial: mortality results after 13 years of follow-up. *J Natl Cancer Inst* 2012;**104**: 125-32.

31. Hayes RB, Reding D, Kopp W, Subar AF, Bhat N, Rothman N, Caporaso N, Ziegler RG, Johnson CC, Weissfeld JL, Hoover RN, Hartge P, et al. Etiologic and early marker studies in the prostate, lung, colorectal and ovarian (PLCO) cancer screening trial. *Control Clin Trials* 2000;**21**: 349s-55s.

32. Barrett-Connor E, Garland C, McPhillips JB, Khaw K-T, Wingard DL. A Prospective, Population-based Study of Androstenedione, Estrogens, and Prostatic Cancer. *Cancer research* 1990;**50**: 169-73.

33. Bycroft C, Freeman C, Petkova D, Band G, Elliott LT, Sharp K, Motyer A, Vukcevic D, Delaneau O, O'Connell J, Cortes A, Welsh S, et al. The UK Biobank resource with deep phenotyping and genomic data. *Nature* 2018;**562**: 203-9.

34. Watts EL, Fensom GK, Smith Byrne K, Perez-Cornago A, Allen NE, Knuppel A, Gunter MJ, Holmes MV, Martin RM, Murphy N, Tsilidis KK, Yeap BB, et al. Circulating insulin-like growth factor-I, total and free testosterone concentrations and prostate cancer risk in 200 000 men in UK Biobank. *Int J Cancer* 2020.

35. UK Biobank. Biomarker assay quality procedures: approaches used to minimise systematic and random errors (and the wider epidemiological implications), 2019.

36. Neuhouser ML, Platz EA, Till C, Tangen CM, Goodman PJ, Kristal A, Parnes HL, Tao Y, Figg WD, Lucia MS, Hoque A, Hsing AW, et al. Insulin-like growth factors and insulin-like growth factor-binding proteins and prostate cancer risk: results from the prostate cancer prevention trial. *Cancer Prev Res (Phila)* 2013;**6**: 91-9.

37. Key TJ, Appleby PN, Allen NE, Reeves GK. Pooling biomarker data from different studies of disease risk, with a focus on endogenous hormones. *Cancer epidemiology, biomarkers & prevention : a publication of the American Association for Cancer Research, cosponsored by the American Society of Preventive Oncology* 2010;**19**: 960-5.

38. Watts EL, Appleby PN, Perez-Cornago A, Bueno-de-Mesquita HB, Chan JM, Chen C, Cohn BA, Cook MB, Flicker L, Freedman ND, Giles GG, Giovannucci E, et al. Low free testosterone and prostate cancer risk: a collaborative analysis of 20 prospective studies. *Eur Urol* 2018;**74**: 585-94.

39. Travis RC, Appleby PN, Martin RM, Holly JM, Albanes D, Black A, Bueno-de-Mesquita HB, Chan JM, Chen C, Chirlaque MD, Cook MB, Deschasaux M, et al. A meta-analysis of individual participant data reveals an association between circulating levels of IGF-I and prostate cancer risk. *Cancer research* 2016;**76**: 2288-300.

40. Ruth KS, Day FR, Tyrrell J, Thompson DJ, Wood AR, Mahajan A, Beaumont RN, Wittemans L, Martin S, Busch AS, Erzurumluoglu AM, Hollis B, et al. Using human genetics to understand the disease impacts of testosterone in men and women. *Nature Medicine* 2020;**26**: 252-8.
41. Day FR, Loh P-R, Scott RA, Ong KK, Perry JRB. A Robust Example of Collider Bias in a Genetic Association Study. *Am J Hum Genet* 2016;**98**: 392-3.
42. Bycroft Cea. Genome-wide genetic data on ~500,000 UK Biobank participants 2017.
43. Schumacher FR, Al Olama AA, Berndt SI, Benlloch S, Ahmed M, Saunders EJ, Dadaev T, Leongamornlert D, Anokian E, Cieza-Borrella C, Goh C, Brook MN, et al. Association analyses of more than 140,000 men identify 63 new prostate cancer susceptibility loci. *Nature Genetics* 2018;**50**: 928-36.
44. Wu L, Wang J, Cai Q, Cavazos TB, Emami NC, Long J, Shu XO, Lu Y, Guo X, Bauer JA, Pasaniuc B, Penney KL, et al. Identification of novel susceptibility loci and genes for prostate cancer risk: a transcriptome-wide association study in over 140,000 European descendants. *Cancer research* 2019;**79**: 3192-204.
45. Burgess S, Bowden J. Integrating summarized data from multiple genetic variants in Mendelian randomization: bias and coverage properties of inverse-variance weighted methods. *arXiv preprint arXiv:151204486* 2015.
46. Bowden J, Del Greco M F, Minelli C, Davey Smith G, Sheehan NA, Thompson JR. Assessing the suitability of summary data for two-sample Mendelian randomization analyses using MR-Egger regression: the role of the I2 statistic. *International Journal of Epidemiology* 2016;**45**: 1961-74.
47. Burgess S, Bowden J, Fall T, Ingelsson E, Thompson SG. Sensitivity Analyses for Robust Causal Inference from Mendelian Randomization Analyses with Multiple Genetic Variants. *Epidemiology* 2017;**28**: 30-42.
48. Verbanck M, Chen C-Y, Neale B, Do R. Detection of widespread horizontal pleiotropy in causal relationships inferred from Mendelian randomization between complex traits and diseases. *Nature Genetics* 2018;**50**: 693-8.
49. Burgess S, Thompson SG. Interpreting findings from Mendelian randomization using the MR-Egger method. *Eur J Epidemiol* 2017;**32**: 377-89.
50. Bowden J, Davey Smith G, Haycock PC, Burgess S. Consistent Estimation in Mendelian Randomization with Some Invalid Instruments Using a Weighted Median Estimator. *Genet Epidemiol* 2016;**40**: 304-14.
51. Burgess S, Foley CN, Allara E, Staley JR, Howson JMM. A robust and efficient method for Mendelian randomization with hundreds of genetic variants. *Nature Communications* 2020;**11**: 376.
52. Holmes MV, Richardson TG, Ference BA, Davies NM, Davey Smith G. Integrating genomics with biomarkers and therapeutic targets to invigorate cardiovascular drug development. *Nat Rev Cardiol* 2021.
53. Hemani G, Zheng J, Elsworth B, Wade KH, Haberland V, Baird D, Laurin C, Burgess S, Bowden J, Langdon R, Tan VY, Yarmolinsky J, et al. The MR-Base platform supports systematic causal inference across the human phenome. *eLife* 2018;**7**: e34408.
